# Multicomponent Stapling of Glucagon‐Like Peptide‐1 Enables Receptor‐Guided PROTAC Delivery

**DOI:** 10.1002/anie.9595531

**Published:** 2026-06-12

**Authors:** Jan L. Venne, Sona Krajcovicova, Graeme Davies, Hannah Bolt, Jefferson Revell, David R. Spring

**Affiliations:** ^1^ Yusuf Hamied Department of Chemistry University of Cambridge Cambridge UK; ^2^ AstraZeneca Plc Cambridge Biomedical Campus 1 Francis Crick Avenue Cambridge UK; ^3^ Department of Organic Chemistry Palacky University Olomouc Olomouc Czech Republic

**Keywords:** brd4, glp‐1, peptide stapling, protac, tryptophan modification

## Abstract

Achieving cell‐selective targeted protein degradation remains a major challenge for translating proteolysis‐targeting chimeras (PROTACs) into therapeutics. Although pancreatic β‐cells are well vascularised and readily accessible to circulating peptides, selective receptor‐mediated drug delivery remains challenging. Here, we exploit the glucagon‐like peptide‐1 receptor (GLP‐1R) as a β‐cell‐specific entry route and report, for the first time, a multicomponent stapled glucagon‐like peptide‐1 (GLP‐1) analogue constructed by tryptophan‐mediated multicomponent Petasis reaction (TMPR). This modular stapling strategy affords a conformationally stabilised GLP‐1 peptide bearing a chemically orthogonal handle for late‐stage conjugation, displaying markedly enhanced α‐helicity and improved receptor potency, compared with the wild‐type peptide. Linking this improved analogue to a bromodomain‐containing protein 4 (BRD4)‐directed degrader furnishes the first GLP‐1‐guided PROTAC, which retains GLP‐1R agonism and induces selective BRD4 degradation in GLP‐1R‐positive cells, consistent with receptor‐guided uptake and intracellular activation of the degrader payload. Together, these results provide strong proof‐of‐concept evidence that a TMPR‐stapled GLP‐1 peptide can serve as a β‐cell‐directed delivery platform for receptor‐defined protein degradation.

## Graphical abstract

We report a stapled glucagon‐like peptide‐1 (GLP‐1) analogue created via multicomponent tryptophan‐mediated Petasis reaction (TMPR). This strategy yields a stabilised peptide with superior helicity and improved potency. Conjugation to a bromodomain‐containing protein 4 (BRD4) degrader creates the first GLP‐1‐guided targeted protein degrader (PROTAC), supporting receptor‐guided uptake and selective degradation in GLP‐1 receptor‐positive cells. TMPR‐stapled peptides thus provide a platform for targeted protein degradation.

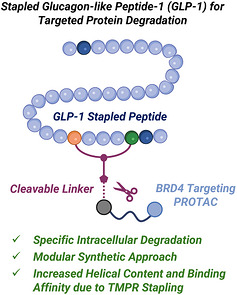

## 1 Introduction

Targeted protein degradation using proteolysis‐targeting chimeras (PROTACs) has transformed chemical biology by enabling catalytic elimination of disease‐relevant proteins rather than stoichiometric inhibition [1]. Yet, their therapeutic potential remains limited by a lack of cell‐type selectivity. Pancreatic β‐cells are an especially appealing but challenging target. They are central to diabetes pathogenesis through the orchestration of insulin secretion, but they are also increasingly implicated in metabolic and inflammatory signalling pathways linked to cancer progression [2], including rare endocrine malignancies like insulinoma [3, 4, 5]. Despite their clinical importance, the inaccessibility of β‐cells and their limited uptake of macromolecules pose significant hurdles for targeted drug or probe delivery.

The glucagon‐like peptide‐1 receptor (GLP‐1R) addresses this challenge by providing an attractive molecular handle for β‐cell‐selective entry, as insulinomas have been shown to overexpress GLP‐1R [4]. This G‐protein‐coupled receptor (GPCR) undergoes efficient ligand‐mediated endocytosis, facilitating the intracellular delivery of receptor‐bound cargos. Although GLP‐1 analogues have been widely explored as incretin mimetics [6, 7], their potential as delivery vehicles for small molecules or bifunctional degraders remains largely unexplored, with a few isolated examples of GLP‐1R–directed in vivo delivery of antisense oligonucleotides [8, 9] and oestrogen molecules [10] reported to date.

**FIGURE 1:**
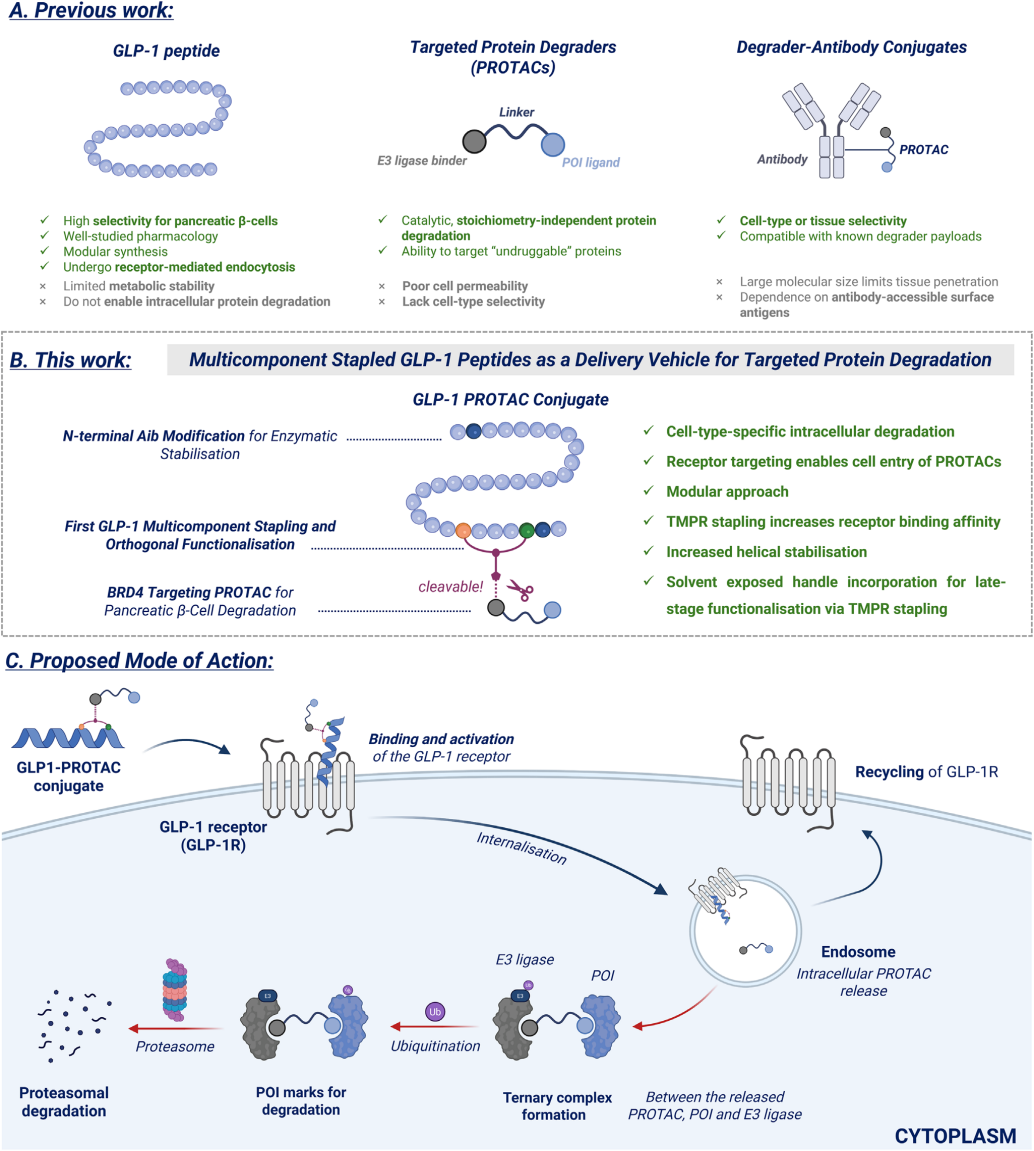
(A,B) Schematic representation of previous versus this work. A 2D projection of the helical GLP‐1 peptides provides a clearer representation of the stapling site. (C) Proposed mode of action of the conjugates: receptor‐guided uptake followed by intracellular cleavage of the masked PROTAC and subsequent BRD4 degradation. 3D helical model of the GLP‐1 peptide for enhanced structural realism (Cartoons created in BioRender. https://BioRender.com/gpf4cer). Abbreviations: Aib = 2‐aminoisobutyric acid; BRD4 = bromodomain‐containing protein 4; GLP‐1R = glucagon‐like peptide‐1 receptor; POI = protein of interest; PROTAC = proteolysis targeting chimeras; TMPR = tryptophan‐mediated Petasis reaction; Ub = ubiquitin.

In parallel, recent efforts to impart selectivity to PROTACs have focused on antibody‐guided degrader constructs, in which monoclonal antibodies are used to deliver degraders to specific cell types [11, 12] (Figure 1A; *Previous work*). While conceptually powerful, they are constrained for GLP‐1R engagement required for internalisation and cargo delivery, as productive agonism of class B GPCRs has proven difficult to achieve with antibodies. Moreover, the size and structural complexity of antibodies impose constraints on tissue penetration, intracellular delivery, and often synthetic ambiguity. These limitations highlight the potential of peptide‐based targeting ligands as a general strategy for receptor‐mediated, cell‐selective protein degradation.

Peptide hormones like GLP‐1 offer high specificity, efficient internalisation, and synthetic tractability, making them ideal β‐cell‐targeting vectors for PROTAC delivery. A key challenge in realising this strategy lies in stabilising the α‐helical structure of GLP‐1, crucial for the receptor activation [13], while simultaneously enabling late‐stage functionalisation for payload attachment. However, existing one‐component hydrocarbon GLP‐1 stapling methods [14] lack the modularity required for such multifunctional conjugates.

To overcome these constraints, we decided to harness our recently developed tryptophan‐mediated multicomponent Petasis reaction (TMPR), between tryptophan, aryl boronic acid and glyoxylic acid, as a versatile multicomponent stapling method for late‐stage peptide macrocyclisation and orthogonal handle installation [15]. TMPR proceeds under mild conditions and generates conformationally constrained analogues amenable to downstream conjugation.

Bearing this in mind, we merge GLP‐1R‐mediated targeting with TMPR stapling to generate, for the first time, a conformationally constrained GLP‐1 analogue suitable for PROTAC conjugation, delivery and targeted protein degradation (Figure 1B; *This work*). In this work, GLP‐1R is used as a selective uptake receptor for intracellular delivery of a masked PROTAC precursor, rather than to induce lysosomal degradation of the target itself. Its role in our system is therefore to enable receptor‐guided internalisation and intracellular activation of a masked degrader payload. To our knowledge, this represents the first example of a peptide hormone‐guided PROTAC that preserves receptor agonism and induces receptor‐dependent protein degradation.

As a proof‐of‐concept, we selected bromodomain‐containing protein 4 (BRD4) as a tractable model for intracellular degradation [11, 16]. A member of the Bromodomain‐and‐Extra‐Terminal Domain (BET) family, BRD4 regulates transcriptional programmes linked to proliferation, inflammation, and stress responses, and its dysregulation is implicated in multiple cancers, including pancreatic neuroendocrine tumours, of which insulinomas are a subtype [17]. Notably, BRD4 is also expressed in pancreatic β‐cells [18], where it modulates transcriptional and inflammatory stress pathways, making it a mechanistically relevant model for testing receptor‐mediated degrader delivery beyond oncogenic systems. With this in mind, we demonstrated GLP‐1R‐mediated protein degradation of BRD4 using a conformationally stabilised GLP‐1 scaffold generated via TMPR‐enabled multicomponent stapling.

## 2 Results and Discussion

### 2.1 Identification of TMPR‐Compatible Stapling Sites in GLP‐1 Peptides

**FIGURE 2:**
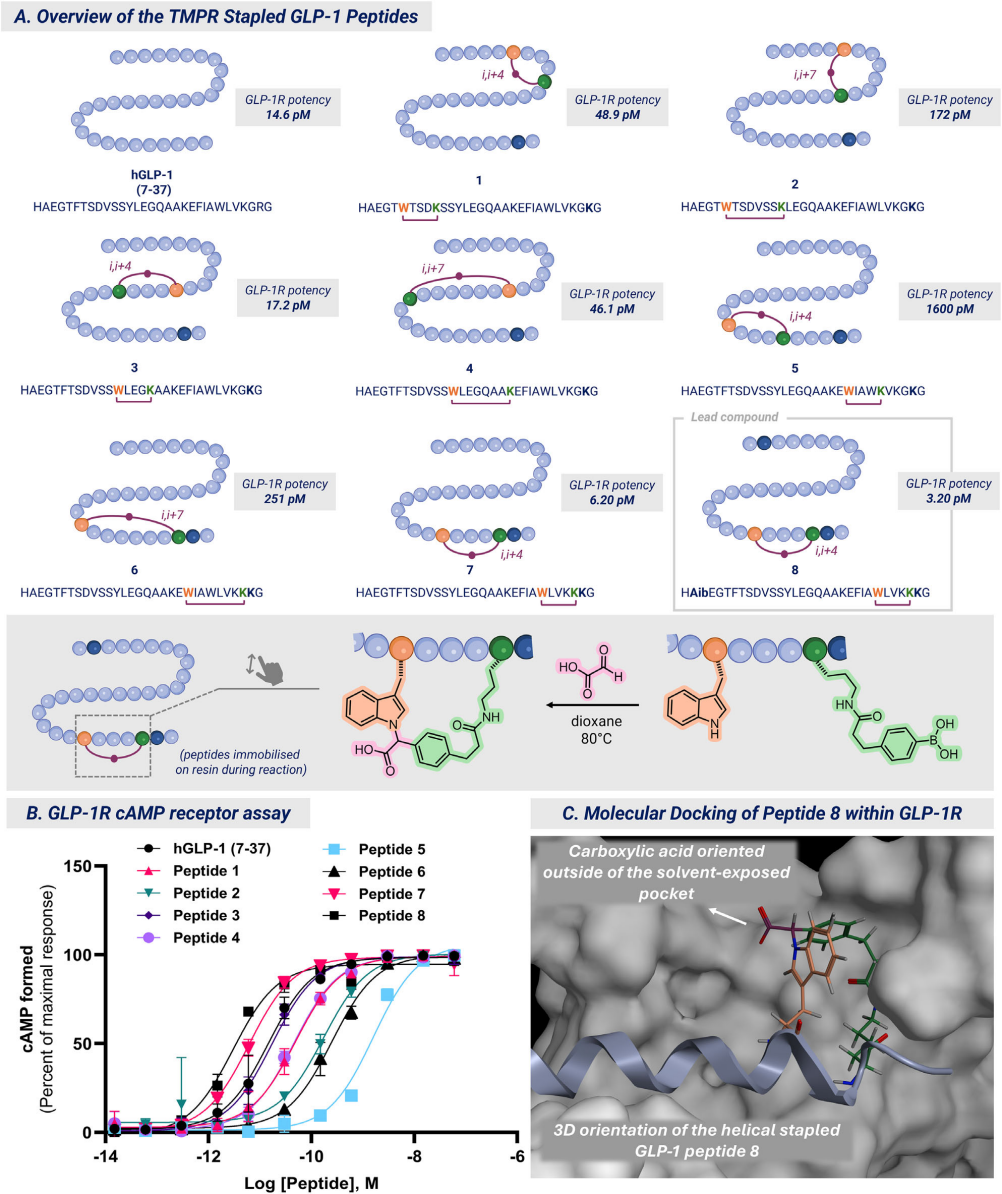
(A) Overview of the TMPR stapled GLP‐1 peptides. A 2D projection of the helical GLP‐1 peptides provides a clearer representation of the stapling sites. Coloured residues indicate the amino acids involved in staple formation (orange = tryptophan, green = lysine), and purple underlining indicates the corresponding stapled sequence region by glyoxylic acid. Peptide **8** was selected as the lead compound. GLP‐1R potency values are shown with the corresponding 95% confidence intervals: **hGLP‐1** = 11.5–18.5 pM; **1** = 40.5–59.2 pM; **2** = 118–250 pM; **3** = 14.6–20.4 pM; **4** = 40–53 pM; **5** = 1300–1960 pM; **6** = 212–297 pM; **7** = 5.40–7.13 pM; **8** = 2.09–5.70 pM; (Cartoons created in BioRender. https://BioRender.com/gpf4cer) (B) GLP‐1R cAMP receptor assay; wild‐type hGLP‐1(7‐37) served as a positive control. Data points represent mean cAMP response normalised to the maximal **hGLP‐1** **(7–37)** response. Data are presented as mean ± SD from three independent experiments (*n* = 3), each performed in technical triplicate. Error bars may be smaller than the symbols. Curves were fitted using a four‐parameter logistic nonlinear regression model. No formal statistical comparisons were performed. (C) Molecular docking of peptide **8** within the GLP‐1R binding pocket, showing orientation of the carboxylic acid towards the solvent and remaining readily accessible for conjugation. Abbreviations: Aib = 2‐aminoisobutyric acid; cAMP = 3′,5′‐cyclic adenosine monophosphate.

To identify positions within GLP‐1 peptides compatible with TMPR‐based stapling, a first series of eight analogues was designed and synthesised (Figure 2A). This step was necessary to establish which stapling geometries could be accommodated without compromising receptor activity, while also generating a solvent‐exposed handle suitable for subsequent functionalisation. Peptides were assembled by automated solid‐phase peptide synthesis and subsequently subjected to on‐resin TMPR stapling [15] to afford analogues **1–7,** alongside wild‐type GLP1 (**hGLP‐1;** Figure 2A). Staple placement was guided by the presence of aromatic residues within the GLP‐1 sequence, which were hypothesised to tolerate tryptophan substitution while preserving receptor activity. For each selected site, both *i,i*+4 and *i,i*+7 stapling patterns were explored. To prevent undesired reactivity of glyoxylic acid during TMPR [15], arginine at position 20 (Arg20) was conservatively replaced with lysine (Lys); this substitution had no measurable impact on potency, selectivity, or cytotoxicity (Figures S1, S2 and S5).

Stapled peptides **1–7** were then evaluated via a homogeneous time‐resolved fluorescence (HTRF) 3′,5′‐cyclic adenosine monophosphate (cAMP) accumulation assay in Chinese hamster ovary (CHO) cells overexpressing GLP‐1R, glucagon receptor (GCGR), or gastric inhibitory polypeptide receptor (GIPR), demonstrating selective agonism for GLP‐1R alone (Figures 2B and S2). Interestingly, peptide **7** exhibited a positive improvement in potency relative to wild‐type GLP‐1, consistent with preservation of tryptophan at position 31 (Trp31) and in line with previous reports indicating that substitutions of glycine at position 35 (Gly35) are well tolerated [19]. Peptide **3** also retained wild‐type activity despite the replacement of tyrosine at position 19 (Tyr19) with tryptophan, whereas alanine (Ala) scanning at this position had previously resulted in a 55‐fold loss of potency, supporting the rationale for targeting aromatic residues for TMPR incorporation. In contrast, analogues **5** and **6**, in which phenylalanine at position 28 (Phe28) and leucine at position 32 (Leu32), respectively, were replaced with TMPR‐stapling residues, showed pronounced reductions in potency (>100‐fold and 17‐fold), in agreement with established structure–activity relationships indicating the importance of these residues for GLP‐1R activity [20]. Importantly, all analogues retained high selectivity for GLP‐1R over GCGR and GIPR and displayed no detectable cytotoxicity (Figures S2 and S5). Peptide **7**, exhibiting higher receptor potency, was then selected as the most promising scaffold for further optimisation. Subsequent substitution of alanine at position 8 (Ala8) with the dipeptidyl peptidase‐4 (DPP‐IV)‐resistant residue 2‐aminoisobutyric acid (Aib) [21] afforded peptide **8**, which, pleasingly, maintained even higher receptor potency than **7** (3.20 vs. 6.20 pM). This analogue was therefore advanced as the primary lead scaffold for subsequent PROTAC conjugation and degradation studies.

### 2.2 Solvent‐Exposed TMPR‐Staple Functionalisation Enables PROTAC Conjugation

**FIGURE 3:**
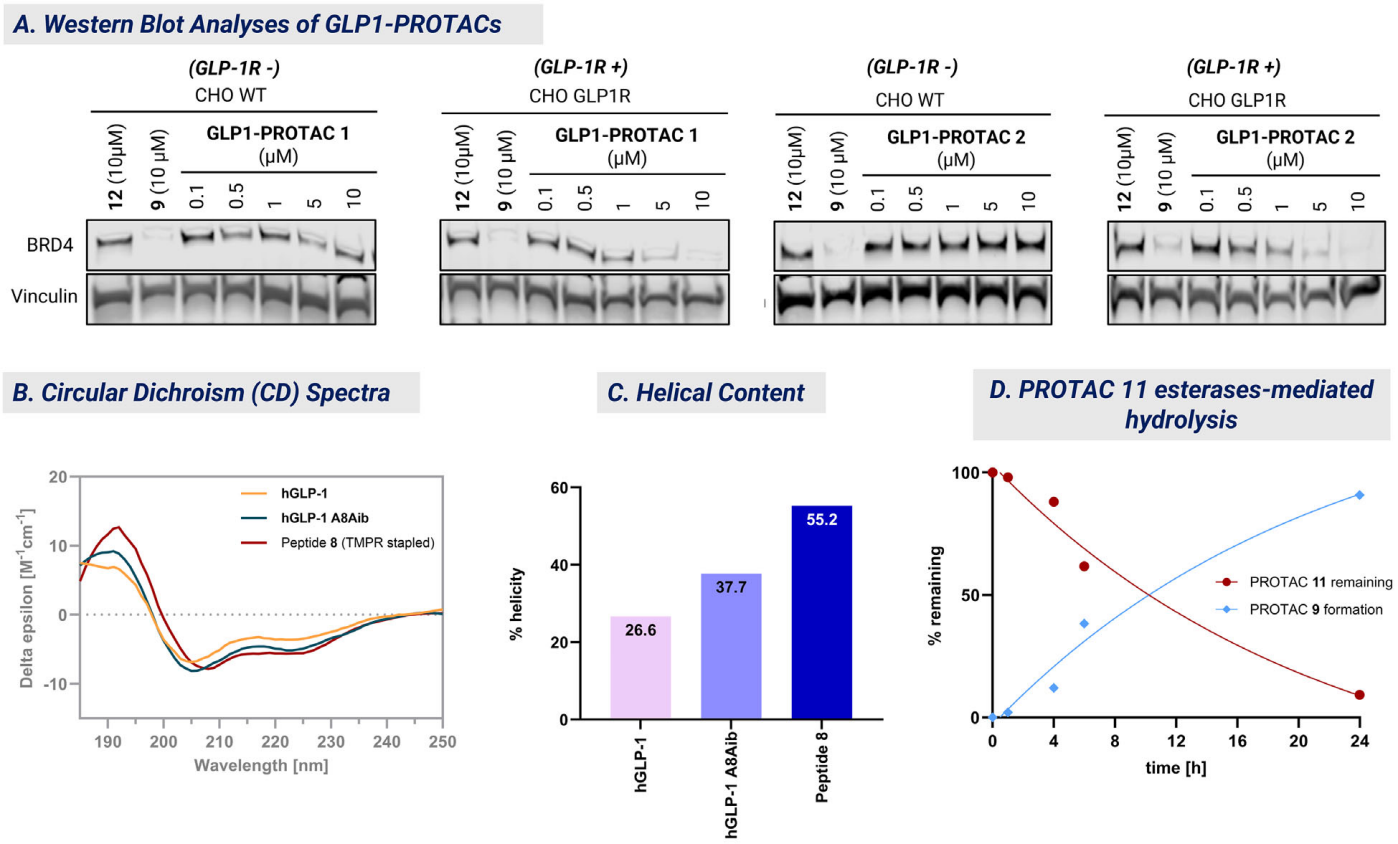
(A) Western Blot analyses of GLP1‐PROTACs 1 and 2 over 24 h. GLP1‐TMPR‐azide **12** serves as a negative control. VHL‐JQ1 **9** serves as a positive control. (For uncropped Western Blots see Section S4.3). (B) Circular dichroism (CD) analysis of wild‐type vs stapled GLP‐1 peptide **8**. (C) Helical content analysis. (See Section S3 for the calculation of the helical content). (D) Esterase‐mediated cleavage of PROTAC **11** serves as a plausible mechanism of the intracellular PROTAC release. Data points represent two individual analytical measurements from a single experiment. Lines are included to guide the eye. No statistical comparisons were performed. *Abbreviations*: A8Aib = alanine‐to‐2‐aminoisobutyric acid substitution at position 8; CHO = Chinese hamster ovary; hGLP‐1 = human glucagon‐like peptide‐1; WT = wild‐type.

Molecular docking studies revealed that the installed staple projects into a solvent‐exposed region of the GLP‐1–GLP‐1R complex, suggesting that this position is well suited for further optimisation and functionalisation without perturbing receptor engagement or signalling (Figure 2C). To assess the impact of TMPR stapling on the secondary structure of GLP‐1, we analysed wild‐type GLP‐1 (**hGLP‐1**), its Aib‐substituted analogue (**hGLP‐1 A8Aib**), and stapled peptide **8** by circular dichroism (CD) spectroscopy (Figure 3B). Pleasingly, all peptides adopted α‐helical conformations, and notably, stapled analogue **8** exhibited a pronounced increase in helical content compared with **hGLP‐1** (55.2% vs. 26.6%; Figure 3C), consistent with conformational preorganisation imposed by the TMPR staple.

GLP‐1 analogues like Exenatide rely on a tryptophan cage motif, in which intramolecular interactions involving a conserved Trp residue stabilise the bioactive helical conformation required for GLP‐1R engagement [22]. We anticipate that TMPR‐mediated backbone stapling will reinforce this cage by restricting conformational flexibility. This favours productive intramolecular contacts, ultimately enhancing both receptor affinity and proteolytic resistance in GLP‐1 analogues [23].

**SCHEME 1:**
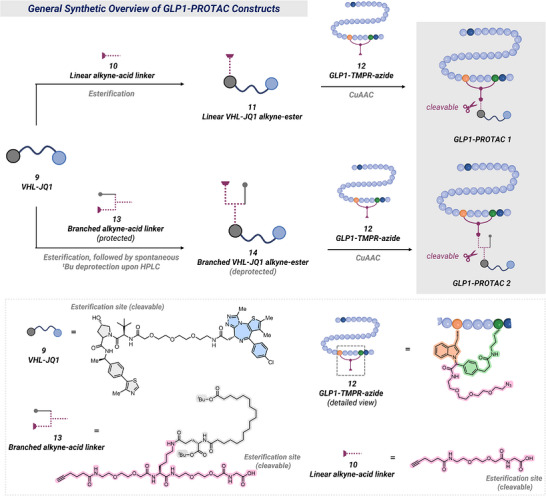
General synthetic overview of GLP1‐ PROTACs. For full synthetic details, see Section S1.3. Esterification conditions: **9**, **10** or **13**, HOBt, DIC, DMAP, DMF, r.t., 16 h; CuAAC conditions: **12**, **11** or **14**, CuSO_4_.5H_2_O, THPTA, sodium ascorbate, dioxane/H_2_O, r.t., 16 h. (Cartoons created in BioRender. https://BioRender.com/gpf4cer) Abbreviations: CuAAC = copper‐catalysed azide–alkyne cycloaddition; DIC = *N,N′*‐diisopropylcarbodiimide; DMAP = 4‐dimethylaminopyridine; DMF = *N,N*‐dimethylformamide; GLP‐1 = glucagon‐like peptide‐1; HOBt = 1‐hydroxybenzotriazole; JQ1 = BRD4 inhibitor named after Jun Qi; Me = methyl; PROTAC = proteolysis‐targeting chimera; r.t. = room temperature; tBu = tert‐butyl; THPTA = tris(3‐hydroxypropyltriazolylmethyl)amine; TMPR = tryptophan‐mediated Petasis reaction; VHL = von Hippel–Lindau.

On the basis of this structural insight, an azide handle was introduced at the solvent‐accessible staple position to generate GLP1‐TMPR‐azide **12** (Scheme 1; see Section S1.3.13 for full synthetic details). Incorporation of this handle created a chemically orthogonal site for late‐stage derivatisation, enabling efficient bioorthogonal conjugation to diverse functional payloads, including PROTACs, while preserving the integrity of the GLP‐1 scaffold.

The chosen BRD4‐degrading PROTAC degrader, as a proof‐of‐concept payload, comprises a JQ1‐derived BET bromodomain ligand linked to a von Hippel–Lindau (VHL)‐recruiting motif, a well‐established architecture exemplified by the BRD4 degrader MZ1 (refers to a scientist M. Zengerle [24]). The chosen construct is a close analogue of MZ1, which has been reported to induce near‐complete BRD4 degradation over 24 h [24, 25]. For its synthesis, the VHL ligand was prepared following established protocols [26], followed by *tert*‐butoxycarbonyl (Boc) deprotection and installation of a polyethyleneglycol (PEG) linker.

JQ1 was obtained as its ester derivative and coupled to the Boc‐deprotected VHL intermediate via hexafluorophosphate azabenzotriazole tetramethyl uronium (HATU)‐mediated amide bond formation to afford the VHL–JQ1 PROTAC **9** (Scheme 1; see Section S1.3.8 for full synthetic details). Importantly, the hydroxyl group of the VHL ligand is critical for productive engagement of the VHL E3 ligase [16, 27]; consequently, installation of an alkyne linker at this position through an ester linkage effectively prevents PROTAC activity. This prodrug‐like design ensures that the degrader remains inactive until intracellular cleavage occurs following targeted delivery [16].

To implement this strategy, **9** was esterified with linear alkyne‐acid linker **10** to afford the linear VHL–JQ1 ester **11**. Subsequent conjugation of **12** to **11** using copper‐catalysed azide‐alkyne cycloaddition (CuAAC) chemistry furnished the final **GLP1‐PROTAC 1** (Scheme 1). For the mechanism of action (Figure 1C), we hypothesised that GLP‐1R engagement would drive receptor‐mediated internalisation of the conjugate, followed by esterase‐mediated cleavage of the ester linker to release the active VHL–JQ1 PROTAC **9** and enable BRD4 degradation.

To probe this activation mechanism and exclude premature release, we evaluated the stability of the PROTAC ester **11** under conditions mimicking extracellular and intracellular environments. Incubation of **11** in phosphate buffer saline (PBS) at 37°C showed no major detectable hydrolysis over 48 h, indicating that the ester linkage is stable under physiological buffer conditions (see Section S3). In contrast, treatment with pancreatin, a mixture of pancreatic esterases, resulted in time‐dependent ester **11** cleavage over 24 h, consistent with enzymatic activation in esterase‐rich cellular environments (Figure 3D). Together, these results are consistent with a model in which the conjugate remains intact during circulation and, following cellular uptake, undergoes intracellular release, likely through esterase‐triggered activation [11, 16]. Having established the structural integrity, stability, and conformational preorganisation of the stapled **GLP1‐PROTAC 1** construct, we next evaluated its functional activity in cellular systems.

### 2.3 GLP‐1‐PROTAC Conjugates Induce GLP‐1R‐Dependent BRD4 Degradation

Biological assessment was performed in BRD4‐expressing CHO cells engineered to express the GLP‐1 receptor, which serves as a well‐established model for pancreatic β‐cell‐like receptor‐mediated uptake [28, 29]. Treatment with **GLP1–PROTAC 1** resulted in time‐dependent degradation of BRD4, as confirmed by Western Blot analysis over a 24 h period (Figure 3A). PROTAC **9** has been used as a positive control, whereas GLP1‐TMPR‐azide **12** as a negative control, where no degradation is expected. Pleasingly, BRD4 depletion by **GLP1‐PROTAC 1** was observed only in CHO GLP‐1R–positive cells, consistent with receptor‐guided uptake of the conjugate followed by intracellular release. In contrast, no BRD4 degradation was detected in CHO GLP‐1R‐negative cells treated under identical conditions (Figure 3A), supporting the conclusion that the observed activity depends on GLP‐1R expression and is not consistent with nonspecific passive uptake alone.

These findings demonstrate that the GLP‐1 moiety functions as an effective targeting vector, enabling selective intracellular delivery of the PROTAC payload while preserving its degradation activity. Treatment of GLP‐1R‐positive cells with the corresponding GLP1–TMPR–azide **12** construct, lacking the PROTAC payload, resulted in no observable changes in BRD4 protein levels. These results show that receptor engagement and internalisation alone are insufficient to elicit the observed biological response, and instead indicate that BRD4 degradation depends on intracellular release of the masked PROTAC payload, consistent with the behavior of reported degrader–antibody conjugates [11].

Collectively, these data provide proof‐of‐concept evidence that conjugation to a GLP‐1 targeting ligand preserves PROTAC function while enabling spatially controlled, cell‐selective protein degradation. Assessment of the binding ability of **GLP1‐PROTAC 1** showed that, despite the presence of the bulky PROTAC moiety, the construct retained excellent GLP‐1R activity, with only a minor reduction in binding potency (Figure S3).

To further extend the platform and address pharmacokinetic considerations, we next prepared a second‐generation construct, **GLP1–PROTAC 2**, via esterification of **9** with branched alkyne‐acid linker **13** bearing a C18 lipid modification (Scheme 1), analogous to the fatty‐acid side chains used in long‐acting GLP‐1 analogues such as Semaglutide [30, 31, 32]. Introduction of the C18 tag was designed to promote reversible albumin binding, thereby increasing circulatory residence time and improving overall conjugate stability. In vitro albumin‐binding assays confirmed strong and sustained association of **GLP1–PROTAC 2** with serum albumin, supporting the effectiveness of the lipidation strategy, while retaining full biological activity of the conjugate (Figure S4). Western blot analysis demonstrated efficient BRD4 degradation in CHO GLP‐1R–positive cells treated with **GLP‐1–PROTAC 2**, comparable to that observed with the non‐lipidated construct (Figure 3A). The results indicate that lipidation does not impair receptor recognition, internalisation, or intracellular release of the active PROTAC degrader, but rather provides an orthogonal handle for pharmacokinetic optimisation while preserving core targeting and degradation mechanisms. The translational potential of this strategy is supported by prior in vivo GLP‐1R‐directed delivery of other cargoes, including antisense oligonucleotides to pancreatic β‐cells [8, 9]. Together with the lipidation strategy introduced here, this provides a basis for future evaluation in animal models.

## 3 Conclusion

In summary, we established a modular GLP‐1–directed PROTAC platform for cell‐selective protein degradation. By integrating peptide‐based targeting, TMPR‐enabled conformational stabilisation, and controlled intracellular release, this strategy supports GLP‐1R‐dependent BRD4 degradation. Conceptually, this distinguishes the present strategy from receptor‐directed lysosomal degradation approaches, as the receptor serves here as a cell‐selective entry route for intracellular release of an active PROTAC, rather than to traffic the target for lysosomal disposal. Central to this advance is the multicomponent tryptophan‐mediated Petasis reaction, which enables multicomponent stapling to enhance helicity and receptor engagement while providing a chemically orthogonal handle for functionalisation. Systematic scanning identified staple positions that tolerate modification, yielding an optimised lead analogue (**8**) that not only retained high receptor activity but showed a 4.6‐fold improvement in GLP‐1R potency relative to the wild‐type peptide. Ligation of caged VHL‐JQ1 PROTACs **11** and **13** to this solvent‐exposed staple afforded **GLP‐1–PROTAC** constructs **1** and **2**, respectively, which preserved GLP‐1R agonism and remained consistent with receptor‐mediated internalisation to induce efficient BRD4 degradation. Collectively, this work demonstrates for the first time that multicomponent stapled GLP‐1 can generate delivery‐competent degraders. More broadly, TMPR chemistry bridges small‐molecule PROTACs and antibody–degrader conjugates, offering a versatile framework for tissue‐selective chemical biology. Given the diversity of internalising peptide hormone receptors, the strategy should be broadly applicable to tissue‐selective protein degradation beyond β‐cells.

## Author Contributions

**Jan L. Venne**: methodology, validation, investigation, formal analysis, writing – original draft, writing – review and editing, conceptualisation. **Sona Krajcovicova**: supervision, investigation, conceptualisation, funding acquisition, writing – original draft, writing – review and editing, visualisation, validation, methodology, formal analysis. **Graeme Davies**: supervision, resources. **Hannah Bolt**: supervision, resources. **Jefferson Revell**: resources, supervision, conceptualisation, data curation. **David R. Spring**: conceptualization, funding acquisition, writing – review and editing, supervision, resources.

## Conflicts of Interest

The authors declare no conflicts of interest.

## Supporting information




**Supporting File**: anie73150‐sup‐0001‐SuppMat.pdf.

## Data Availability

The data that support the findings of this study are available in the Supporting Information of this article
