# Supplementary material for: Multicomponent Stapling of Glucagon‐Like Peptide‐1 Enables Receptor‐Guided PROTAC Delivery
Source: Angew Chem Int Ed Engl. 2026 Jun 12;65(34):e9595531. doi: 10.1002/anie.9595531 (PMC13480816; doi:10.1002/anie.9595531)
Supplement: Supplementary file 1 [file ANIE-65-e9595531-s001.pdf]

Supporting Information

Multicomponent Stapling of Glucagon-like Peptide-1 Enables Receptor-Guided PROTAC Delivery

Jan L. Venne, Sona Krajcovicova*, Graeme Davies, Hannah Bolt, Jefferson Revell, and David R. Spring*

Table of Contents

1. EXPERIMENTAL PROCEDURES 3

1.1. General Information 3

1.2. Instrumentation 4

1.3. Experimental Procedures 5

1.3.1. General Procedure A: Deprotection of Alloc protecting group 5
1.3.2. General Procedure B: Amide coupling with 4-(2 carboxyethyl)benzene boronic acid 5
1.3.3. General Procedure C: Tryptophan-mediated Petasis reaction (TMPR) with peptides on resin 5
1.3.4. General Procedure D: Manual Fmoc deprotection on resin 5
1.3.5. General Procedure E: Manual amide coupling on resin 5
1.3.6. General Schematic Representation of the Stapling Modification via TMPR 6
1.3.7. Synthesis of TMPR stapled GLP-1 peptides 7
1.3.8. Synthesis of VHL-JQ1 PROTAC **9** 18
1.3.9. Synthesis of linear alkyne-acid linker **10** 20
1.3.10. Synthesis of branched alkyne-acid linker **13** 21
1.3.11. Synthesis of linear VHL-JQ1 alkyne-ester **11** 22
1.3.12. Synthesis of branched VHL-JQ1 alkyne-ester **14** 23
1.3.13. Synthesis of GLP1-TMPR-azide **12** 24
1.3.14. Synthesis of **GLP1-PROTAC 1** 26
1.3.15. Synthesis of **GLP1-PROTAC 2** 27

2. ANALYTICAL AND UV LCMS SPECTRA 28

3. BIOPHYSICAL AND STABILITY DATA 45

4. BIOLOGICAL EVALUATION OF COMPOUNDS 1–8 AND GLP1-PROTACS 47

4.1. Cell Culture 47

4.2. cAMP accumulation 47

4.3. Western Blot 51

4.4. CellTiter-Glo® 2.0 Cell Viability Assay 53

5. NMR SPECTRA 55

1. Experimental Procedures

1.1. General Information

All reagents were of reagent grade and used without further purification. Solvents and chemicals were purchased from Sigma-Aldrich (US), Acros Organics (BE), Fluorochem (UK) or Iris Biotech (DE). Anhydrous solvents were stored as received from commercial suppliers. Software used: chemical structures (Chemdraw), NMR (MestreNova), UV LCMS (MassLynx), analytical HPLC (Prism), figures (created in BioRender https://BioRender.com/5jhvcvq or MS Powerpoint), helical content (Bestsel), citations (Zotero).

Automated peptide synthesis was carried out on a CEM Liberty Automated Microwave Peptide Synthesiser using Rink Amide MBHA resin LL (100-200 mesh, loading 0.42 mmol/g). The scale for resin-bound compounds was 0.1 mmol. All automated amide couplings were performed with Fmoc-protected amino acids (0.2 M), HOBt (1 M) and DIC (1 M) as scoupling reagents in DMF. After automated synthesis all resin-bound compounds were transferred to plastic syringes each equipped with a porous disk, washed with CH_2Cl_2 (5 × 10 mL), DMF (2 × 10 mL) and CH_2Cl_2 again (2 × 10 mL) and stored dried in a freezer. Fmoc deprotection was performed with 20% piperidine in DMF. Protecting groups used during solid-phase peptide synthesis: *t*-Bu (Asp, Glu, Tyr, Ser, Thr); Boc (Lys, His, Trp); Pbf (Arg); Trt (Gln, Asn).

For manual solid-phase synthesis reactions were carried out in plastic reaction vessels (syringes, each equipped with a porous disk from Torviq, US, www.torviq.com), using 2-chlorotrityl chloride resin pre-modified with H-Gly-OH (loading 0.84 mmol/g) or Rink Amide MBHA resin (0.4 mmol/g). All external reagents were dissolved in a glass beaker in an appropriate solvent and added to the resin at once, unless stated otherwise. The volume of used solvent was 10 mL per 1 g of resin, unless stated otherwise. All manual solid-phase reactions were performed at ambient temperature, unless stated otherwise. For manual washing, resin slurry was shaken with the fresh solvent for at least 1 min before changing the solvent. UV-LCMS analysis was performed after each reaction step (except for Fmoc cleavage) to ensure completeness of the synthesis, unless stated otherwise.

Upon peptide synthesis, analytical UV-LCMS analysis was performed as follows: analytical sample of a resin (~5 mg) was transferred into a plastic Eppendorf tube, treated with a cleavage cocktail (**Rink amide:** CH_2Cl_2/TFA 1:1, 1 mL, v/v; **2-Cl-Trt:** CH_2Cl_2/HFIP 2:1, 1.5 mL, v/v) and shaken for 1–3 hours at ambient temperature. For peptides containing trityl protecting group, 100 µL of TIPS was added to a cleavage cocktail with TFA as a scavenger. The cleavage cocktails were then evaporated under a stream of nitrogen, cleaved compounds were crashed out with Et_2O, dried and extracted into MeCN/H_2O (1:1 v/v, 1.5 mL), filtered, and submitted to UV-LCMS analysis to determine purity of the prepared compounds.

For full cleavage of peptides from Rink amide resin prior to HPLC purification a cleavage cocktail of TFA/TIPS/H_2O (18:1:1, 20 mL, v/v; unless stated otherwise) was used. Cleavage cocktail was evaporated under reduced pressure. Crude was then crashed out with Et_2O (10mL), dried, and solubilised in MeCN/H_2O/TFA (6:4:0.1, 5 mL) before purification. Overall yields after peptide synthesis were calculated from initial resin loadings.

For full cleavage of compounds from the 2-chlorotrityl resin a cleavage cocktail of CH_2Cl_2/HFIP (4:1, 12.5 mL, v/v) was used and the cleavage time was prolonged to 4 hours (usually 2 × 2-hour cycles were performed). Cleavage cocktails were evaporated under reduced pressure, and crude product was carried on without further purification.

1.2. Instrumentation

UV LCMS analysis was carried out using Waters ACQUITY H-Class UPLC with an ESCi Multi-Mode Ionisation Waters SQ Detector 2 spectrometer using ACQUITY UPLC® CSH C18 (2.1 mm × 50 mm, 1.7 μM, 130 Å) column at 40 °C and flow rate 0.6 mL/min. Mobile phase was (A) 2 mM ammonium acetate in water/MeCN (95:5), (B) MeCN and (C) 2% aq. formic acid, linearly programmed from 5–95% B over 5 min with constant 5% C over 1 min. The ESI source operated at a discharge current of 5 μA, vaporiser temperature of 350 °C and capillary temperature of 200 °C. Chromatographs were monitored by UV absorbance using a photodiode array detector at a wavelength range of 210–800 nm, interval 1.2 nm.

Preparative HPLCs were carried out on Knaur preparative HPLC using a SunFire® Prep C18 OBD™ (30 mm x 250 mm, 5 μM) eluting with a linear gradient system with mobile phases (A) 0.1% TFA in water (v/v) and (B) 0.05% TFA in MeCN (v/v) over 30 min at a flow rate of 60 mL/min, or with mobile phases (A) 100mM, pH 6.6 $(NH_4)_2C_6H_6O_7$ in water and (B) MeCN over 30 min at a flow rate of 60 mL/min, and on Agilent 1260 Infinity using a Supelcosil ABZ+PLUS column (250 mm × 21.2 mm, 5 μM) eluting with a linear gradient system with mobile phases (A) 0.1% TFA in water (v/v) and (B) 0.05% TFA in MeCN (v/v) over 20 min at a flow rate of 20 mL/min.

Analytical HPLCs were carried out using Agilent 1260 with a reversed-phase Polaris 5 C8-A (100 mm x 4.6 mm, 3 μM) column eluting with a linear gradient system (solvent A: 0.05% (v/v) TFA in water, solvent B: 0.05% (v/v) TFA in MeCN) over 15 minutes, at a flow rate of 1 mL/min and Agilent 1260 or 1200 Infinity systems with a reversed-phase Supelcosil™ ABZ+PLUS column (150 mm × 4.6 mm, 3 μM), Agilent Eclipse XDB C18, (5 μm, 4.6 x 150 mm) column, or Agilent AdvanceBio Peptide Mapping (120Å pore size, 4.6 x 150 mm, 2.7 μm particle size) column eluting with a linear gradient system (solvent A: 0.05% (v/v) TFA in water, solvent B: 0.05% (v/v) TFA in MeCN) over 15 minutes, at a flow rate of 1 mL/min. Analytical HPLC was monitored by UV absorbance at 220, 254 and 280 nm.

Mass-directed automated HPLC purification (MDAP HPLC) was carried out on Waters AutoPurification mass-directed HPLC system with photodiode array detector, SQ Detector 2 and at-column dilution. Analytical runs used an X-Select CSH C18 column (4.6 × 150mm, 5 μM particle size) and flow rate of 1.2 mL/min. Preparative runs used an X-Select CSH Prep C18 OBD column (19 × 150mm, 5 μM particle size) and flow rate of 20 mL/min. The machine used a linear gradient of solvent B in solvent A run over 15 min with 1 min isocratic hold before the gradient (analytical) or over 15 min with 1.4 min isocratic hold (preparative). For acidic runs, solvent A was water with 0.1% (v/v) formic acid and solvent B was MeCN with 0.1% (v/v) formic acid. For neutral runs solvent A was water (no additives) and solvent B was MeCN (no additives).

NMR spectra were recorded on Bruker Avance III 500 MHz HD Smart Probe and Bruker TXO 700 MHz Cryo Probe spectrometers at magnetic field strengths of 11.75 T (500 MHz) and 16.44 T (700 MHz) with operating frequencies 500.16 MHz and 700.13 MHz (for ^{1}H), 125.77 MHz and 176.04 MHz (for ^{13}C) at 23°C. Chemical shifts (δ) are reported in parts per million (ppm) and coupling constants (*J*) are reported in Hertz (Hz). The 1H, 13C NMR and DEPT-135 chemical shifts (δ in ppm) were referenced to the residual signals of DMSO-*d6* [2.50 (^{1}H) and 39.52 (^{13}C)] and $CDCl_3$ [7.26 (^{1}H) and 77.16 (^{13}C)]. Abbreviations in NMR spectra: app – apparent, br – broad, d – doublet, dd – doublet of doublets, m – multiplet, p – pentet, s – singlet, t – triplet.

1.3. Experimental Procedures

1.3.1. General Procedure A: Deprotection of Alloc protecting group

Corresponding resin has been washed with CH_2Cl_2 (3 × 10 mL). $Pd(PPh_3)_4$ (0.02M) has been dissolved in CH_2Cl_2 (4 mL per 300 mg of resin) and $PhSiH_3$ (0.2M) has been added subsequently. The reaction mixture was added to a plastic syringe with the resin at once and the syringe has been stabbed with a needle to release pressure. The slurry has been shaken for 4 hours at ambient temperature after which it changed colour to dark brown that indicated full deprotection of Alloc. Resin has been washed with CH_2Cl_2 (3 × 10 mL), DMF (3 × 15 mL) and CH_2Cl_2 (5 × 10 mL), small portion has been cleaved and analysed by UV-LCMS.

1.3.2. General Procedure B: Amide coupling with 4-(2 carboxyethyl)benzene boronic acid

Corresponding Alloc deprotected resin has been washed with CH_2Cl_2 (3 × 10 mL). Commercially available 4-(2-carboxyethyl)benzene boronic acid (0.3M) and HATU (0.3M) have been dissolved in DMF (2.5 mL per 250 mg of resin), followed by addition of DIPEA (0.6M). Reaction mixture has been added to a plastic syringe with the resin at once and slurry has been shaken for 16 hours. Resin has been washed with CH_2Cl_2 (3 × 10 mL), DMF (3 × 15 mL) and CH_2Cl_2 (5 × 10 mL), small portion has been cleaved and analysed by UV-LCMS.

1.3.3. General Procedure C: Tryptophan-mediated Petasis reaction (TMPR) with peptides on resin

Corresponding resin has been washed with CH_2Cl_2 (3 × 10 mL) and transferred to a conical vial. Glyoxylic acid monohydrate (0.6M) has been dissolved in 1,4-dioxane (2 mL per 100 mg of resin) and added to the conical vial with a resin. Reaction mixture has been stirred at 80 °C for 16 hours. Upon reaction, peptides have been cleaved off the resin and purified by preparative HPLC.

1.3.4. General Procedure D: Manual Fmoc deprotection on resin

Corresponding resin was washed with CH_2Cl_2 (3 × 10 mL). Manual Fmoc deprotection was performed with 5% DBU in CH_2Cl_2 in a plastic syringe at ambient temperature for 15 minutes. The resin was washed with CH_2Cl_2 (3 ×10 mL), DMF (3 ×15 mL) and CH_2Cl_2 (5 ×10 mL) and used in the next step without further analysis.

1.3.5. General Procedure E: Manual amide coupling on resin

Corresponding Fmoc deprotected resin was washed with CH_2Cl_2 (3 × 10 mL). Corresponding carboxylic acid (0.2 M) and HOBt (0.2 M) were dissolved in DMF, followed by addition of DIC (0.2 M) prior to the addition to the resin. The reaction mixture was added to a plastic syringe with the resin at once and the slurry was shaken for 1–3 hours. The resin was washed with CH_2Cl_2 (3 × 10 mL), DMF (3 × 15 mL) and CH_2Cl_2 (5 × 10 mL) and small portions were cleaved and analysed by UV-LCMS.

1.3.6. General Schematic Representation of the Stapling Modification via TMPR

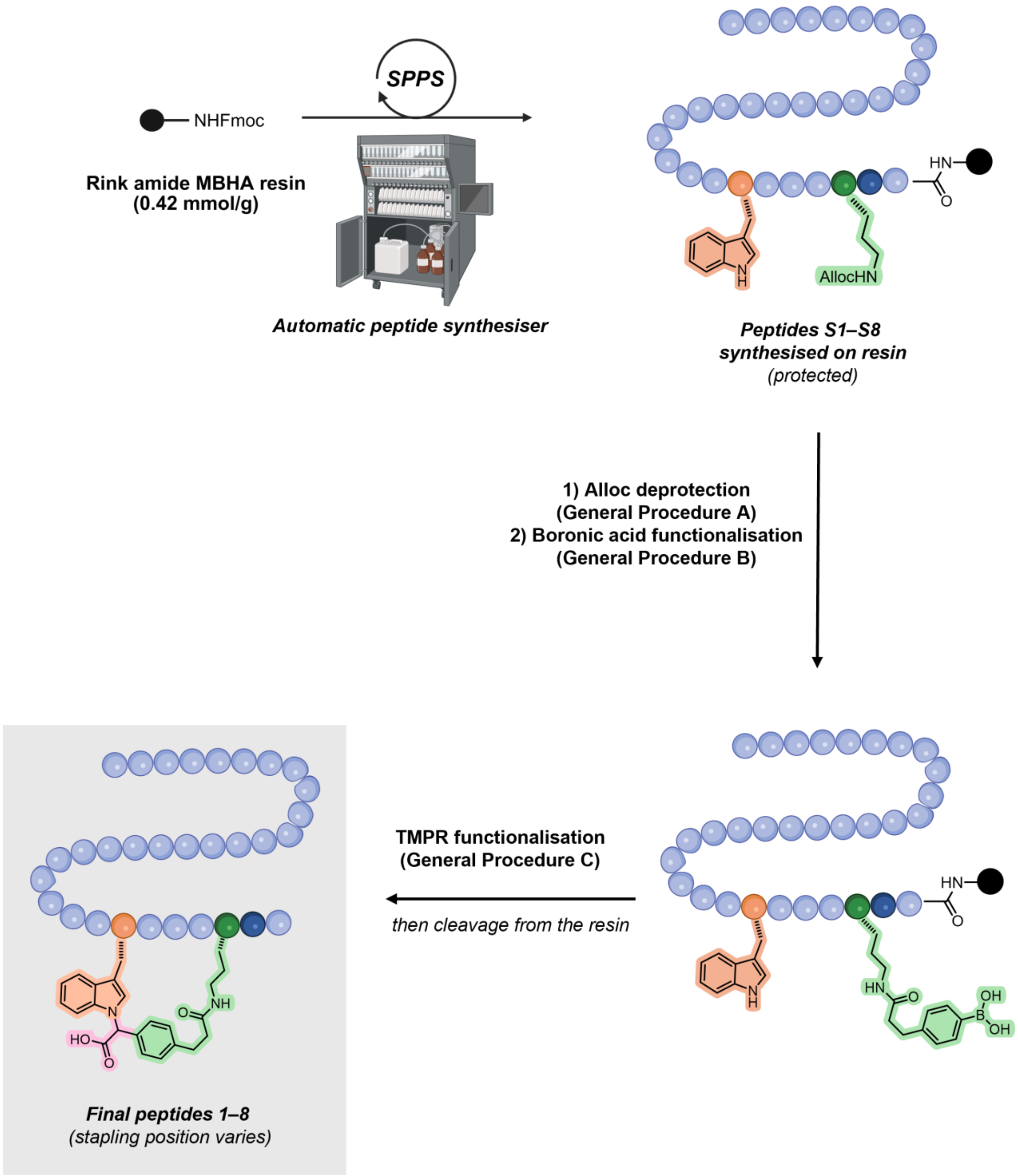

1.3.7. Synthesis of TMPR stapled GLP-1 peptides

- ***Peptide hGLP-1***

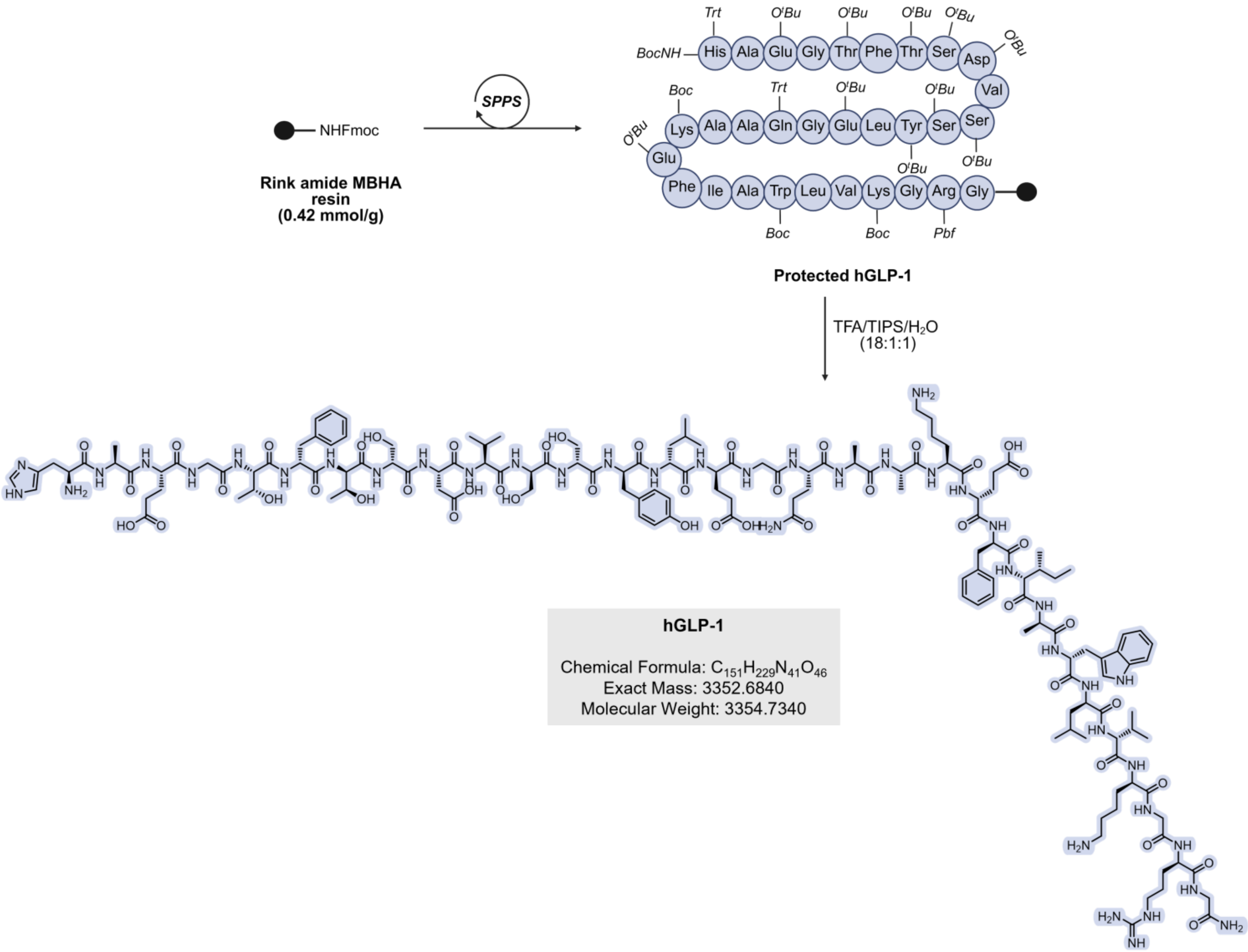

Protected **hGLP-1** peptide has been obtained by automated peptide synthesis, upon which it has been cleaved from the Rink amide resin and purified via preparative HPLC to obtain compound **hGLP-1** as a white solid (65 mg, overall yield 19%).

Analytical HPLC: R_t = 5.88 min (10–90% B over 15 min)
Purity: >99%
LRMS calcd. for $C_{151}H_{231}N_{41}O_{46}^{2+}$ *m/z* 3354.6986 $[M+2H]^{2+}$, found 1119.66 $[M/3+2H]^{2+}$.

- ***Peptide hGLP-1 R36K***

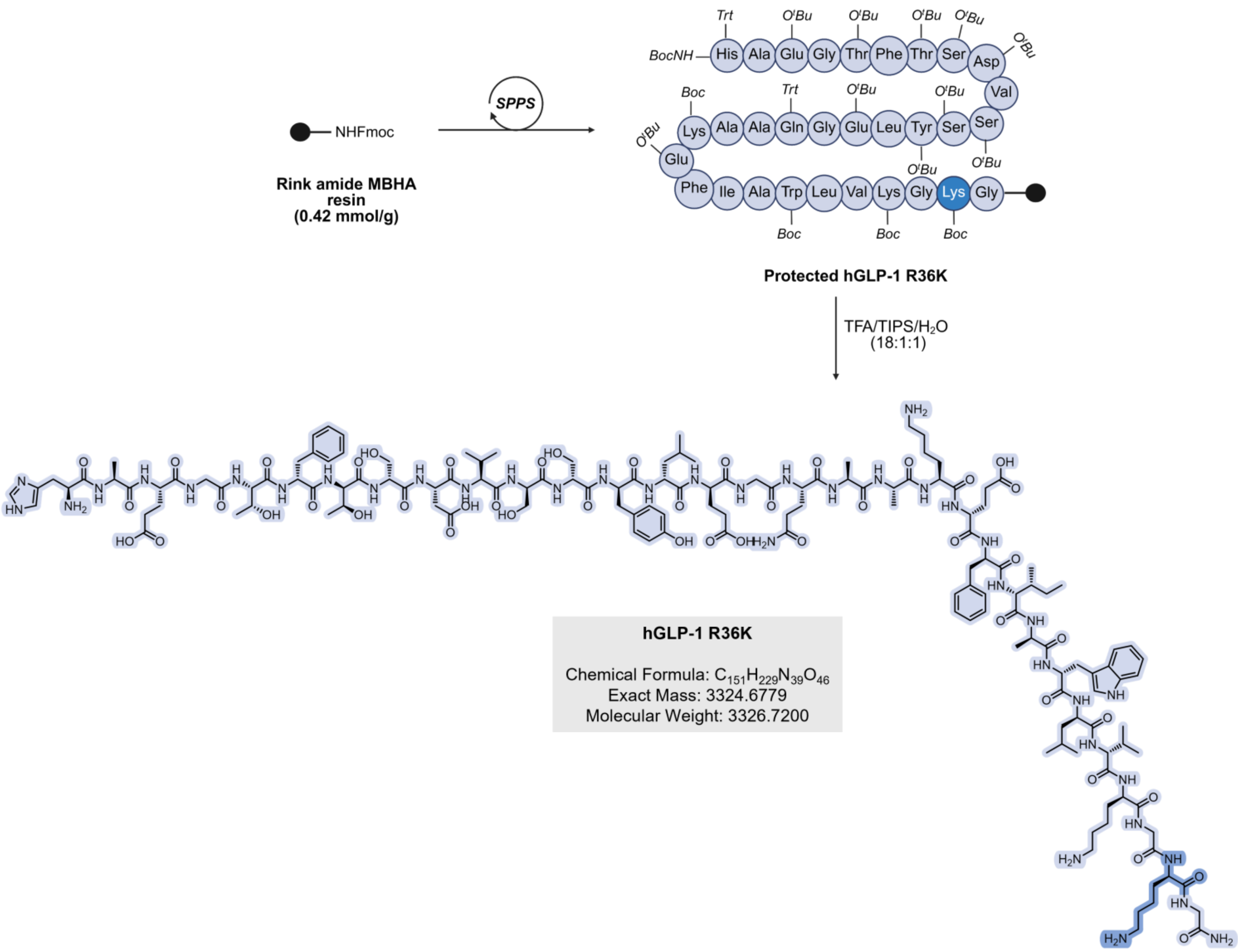

Protected **hGLP-1 R36K** peptide has been obtained by automated peptide synthesis, upon which it has been cleaved from the Rink amide resin and purified via preparative HPLC to obtain compound **hGLP-1 R36K** as a white solid (72 mg, overall yield 21%).

Analytical HPLC: R_t = 5.89 min (10–90% B over 15 min)
Purity: >99%
LRMS calcd. for $C_{151}H_{231}N_{39}O_{46}^{2+}$ *m/z* 3326.6924 $[M+2H]^{2+}$, found 1110.30 $[M/3+2H]^{2+}$.

- ***Peptide hGLP-1 A8Aib***

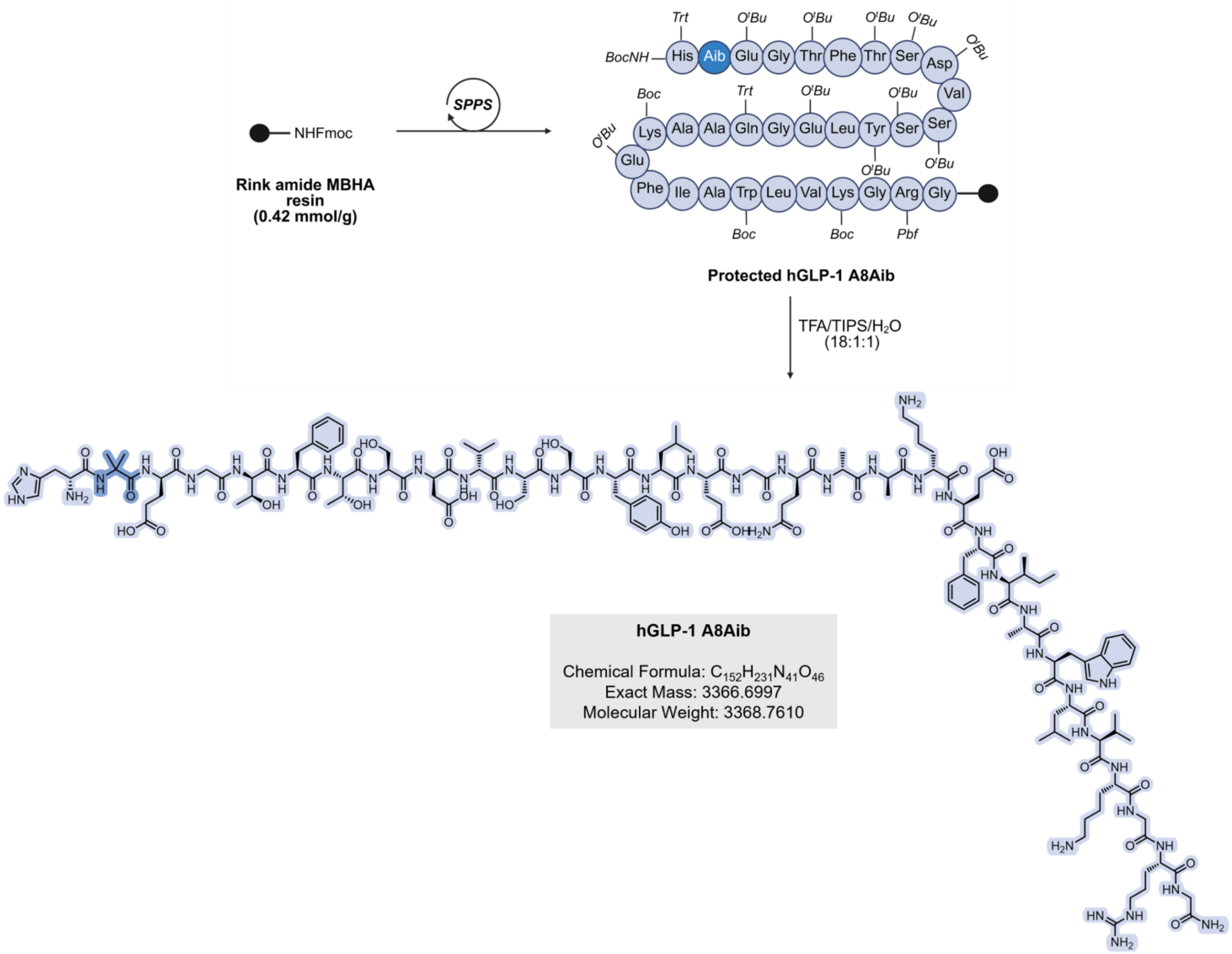

Protected **hGLP-1 A8Aib** peptide has been obtained by automated peptide synthesis, upon which it has been cleaved from the Rink amide resin and purified via preparative HPLC to obtain compound **hGLP-1 A8Aib** as a white solid (11 mg, overall yield 4%).

Analytical HPLC: R_t = 5.91 min (10–90% B over 15 min)
Purity: >99%
LRMS calcd. for $C_{152}H_{234}N_{41}O_{46}^{3+}$ *m/z* 3369.7215 $[M+3H]^{3+}$, found 1123.75 $[M/3+3H]^{3+}$.

- ***Peptide 1***

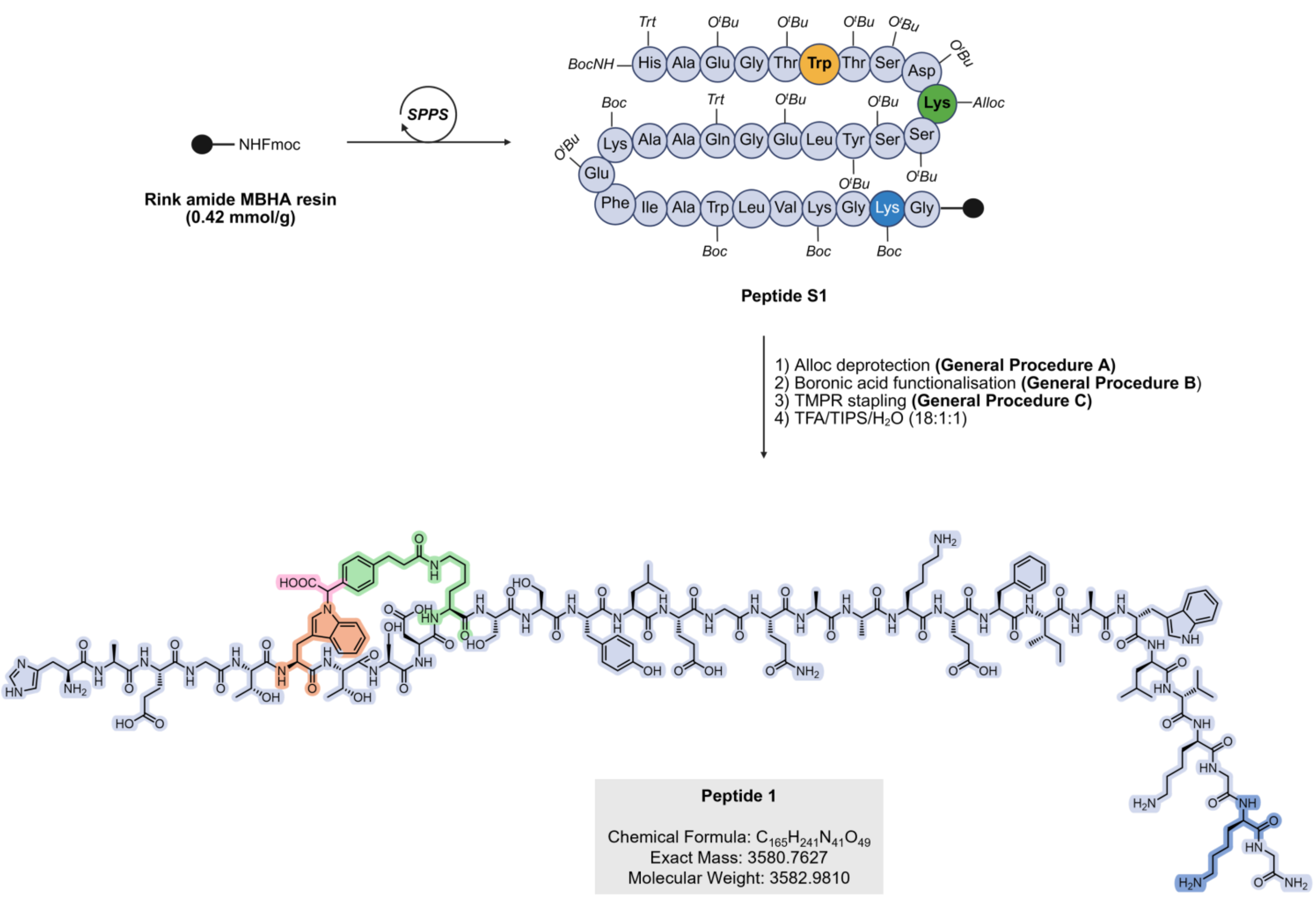

Peptide **S1** has been obtained by automated peptide synthesis, followed by deprotection of Alloc (*1.3.1. General Procedure A*), amide coupling with 4-(2 carboxyethyl)benzene boronic acid (*1.3.2. General Procedure B*) and stapled via TMPR (*1.3.3. General Procedure C*). Upon stapling, it has been cleaved from the Rink amide resin and purified via preparative HPLC to obtain compound **1** as a white solid (0.4 mg, overall yield 2%).

Analytical HPLC R_t = 5.15 min (10–90% B over 15 min)
Purity: 96%
LRMS calcd. for $C_{165}H_{244}N_{41}O_{49}^{3+}$ *m/z* 3583.7845 $[M+3H]^{3+}$, found 1210.49 $[M/3+H+2Na]^{3+}$.

- ***Peptide 2***

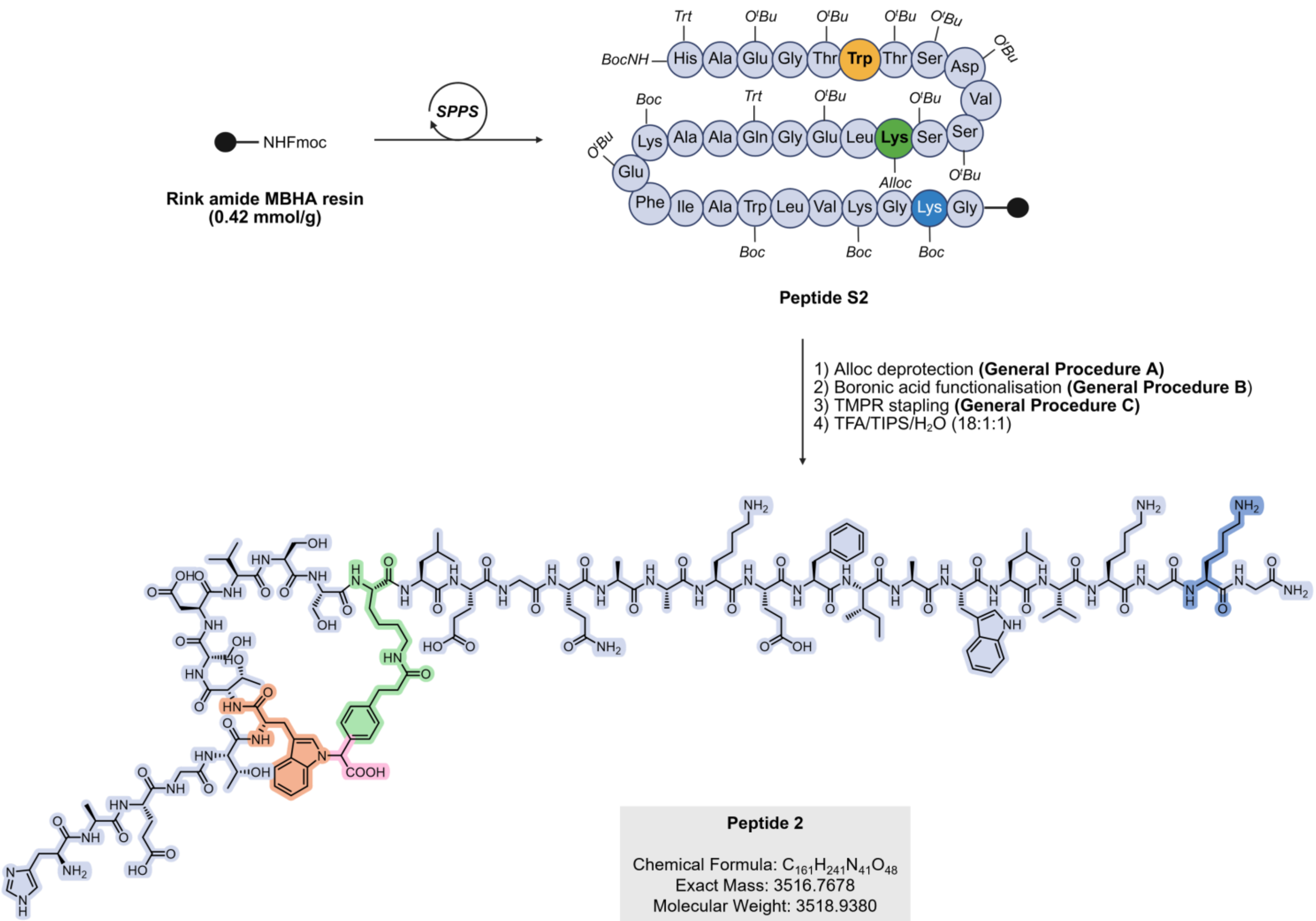

Peptide **S2** has been obtained by automated peptide synthesis, followed by deprotection of Alloc (*1.3.1. General Procedure A*), amide coupling with 4-(2 carboxyethyl)benzene boronic acid (*1.3.2. General Procedure B*) and stapled via TMPR (*1.3.3. General Procedure C*). Upon stapling, it has been cleaved from the Rink amide resin and purified via preparative HPLC to obtain compound **2** as a white solid (1.2 mg, overall yield 4%).

Analytical HPLC R_t = 5.20 min (10–90% B over 15 min)

Purity: 97%

LRMS calcd. for $C_{161}H_{244}N_{41}O_{48}^{3+}$ *m/z* 3519.7896 $[M+3H]^{3+}$, found 1188.79 $[M/3+H+2Na]^{3+}$.

- ***Peptide 3***

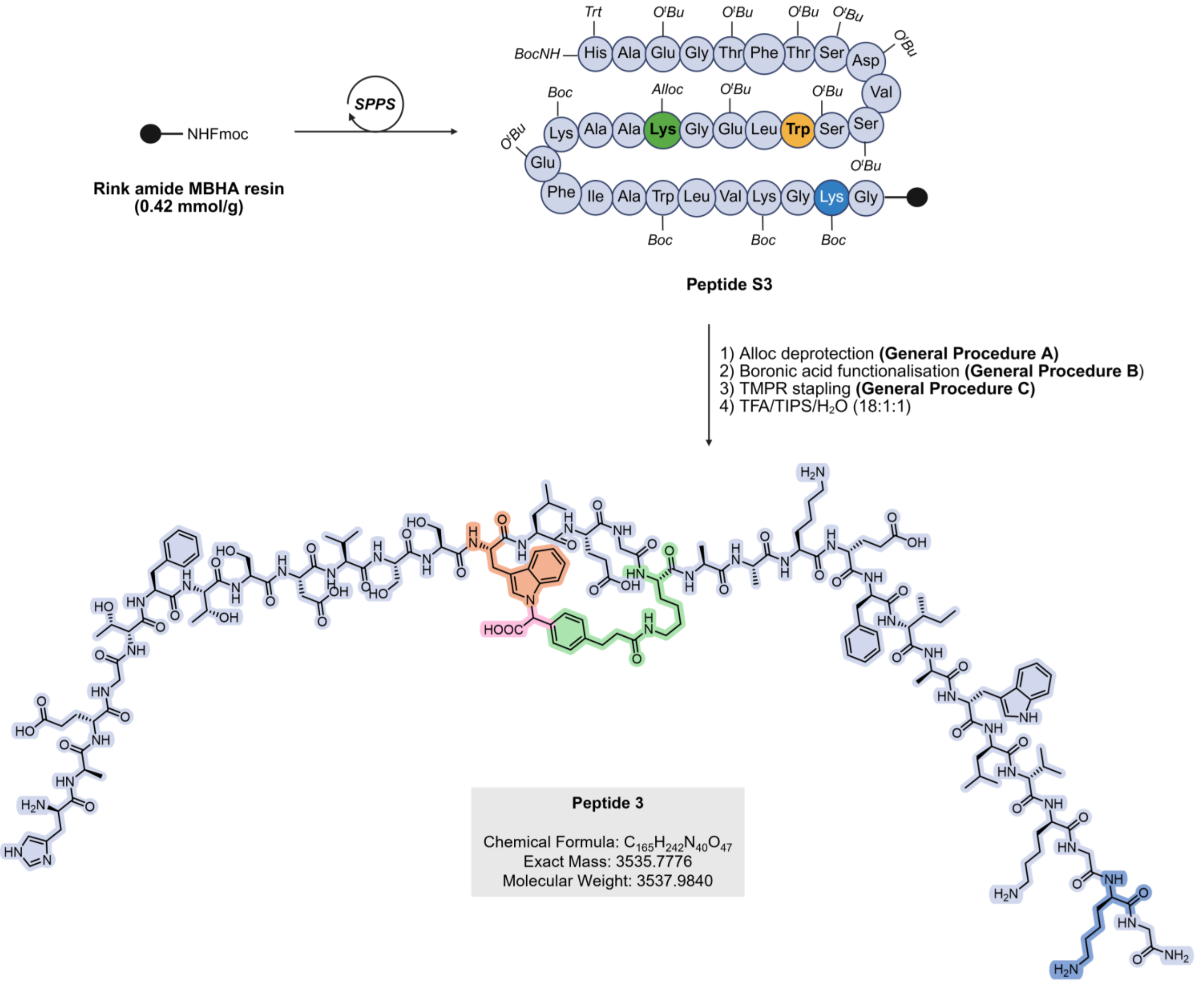

Peptide **S3** has been obtained by automated peptide synthesis, followed by deprotection of Alloc (*1.3.1. General Procedure A*), amide coupling with 4-(2 carboxyethyl)benzene boronic acid (*1.3.2. General Procedure B*) and stapled via TMPR (*1.3.3. General Procedure C*). Upon stapling, it has been cleaved from the Rink amide resin and purified via preparative HPLC to obtain compound **3** as a white solid (2.9 mg, overall yield 8%).

Analytical HPLC: R_t = 5.64 min (10–90% B over 15 min)

Purity: >99%

LRMS calcd. for $C_{165}H_{245}N_{40}O_{47}^{3+}$ *m/z* 3538.7994 $[M+3H]^{3+}$, found 1195.45 $[M/3+H+2Na]^{3+}$.

- ***Peptide 4***

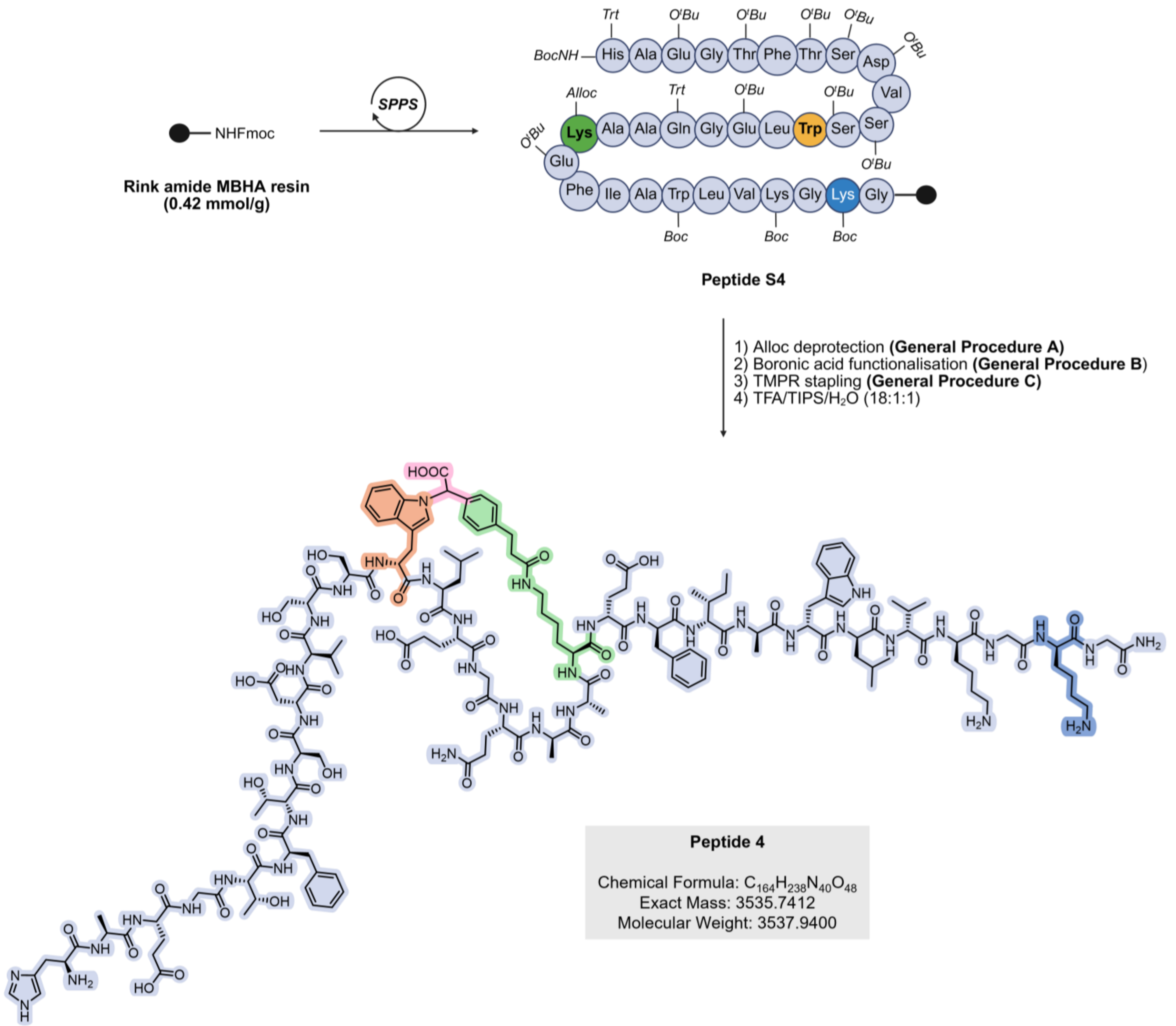

Peptide 4

Chemical Formula: $C_{164}H_{238}N_{40}O_{48}$
Exact Mass: 3535.7412
Molecular Weight: 3537.9400

Peptide **S4** has been obtained by automated peptide synthesis, followed by deprotection of Alloc (*1.3.1. General Procedure A*), amide coupling with 4-(2 carboxyethyl)benzene boronic acid (*1.3.2. General Procedure B*) and stapled via TMPR (*1.3.3. General Procedure C*). Upon stapling, it has been cleaved from the Rink amide resin and purified via preparative HPLC to obtain compound **4** as a white solid (0.3 mg, overall yield 2%).

Analytical HPLC: R_t = 5.83 min (10–90% B over 15 min)
Purity: 97%
LRMS calcd. for $C_{164}H_{241}N_{40}O_{48}^{3+}$ *m/z* 3538.7630 $[M+3H]^{3+}$, found 1195.74 $[M/3+H+2Na]^{3+}$.

- ***Peptide 5***

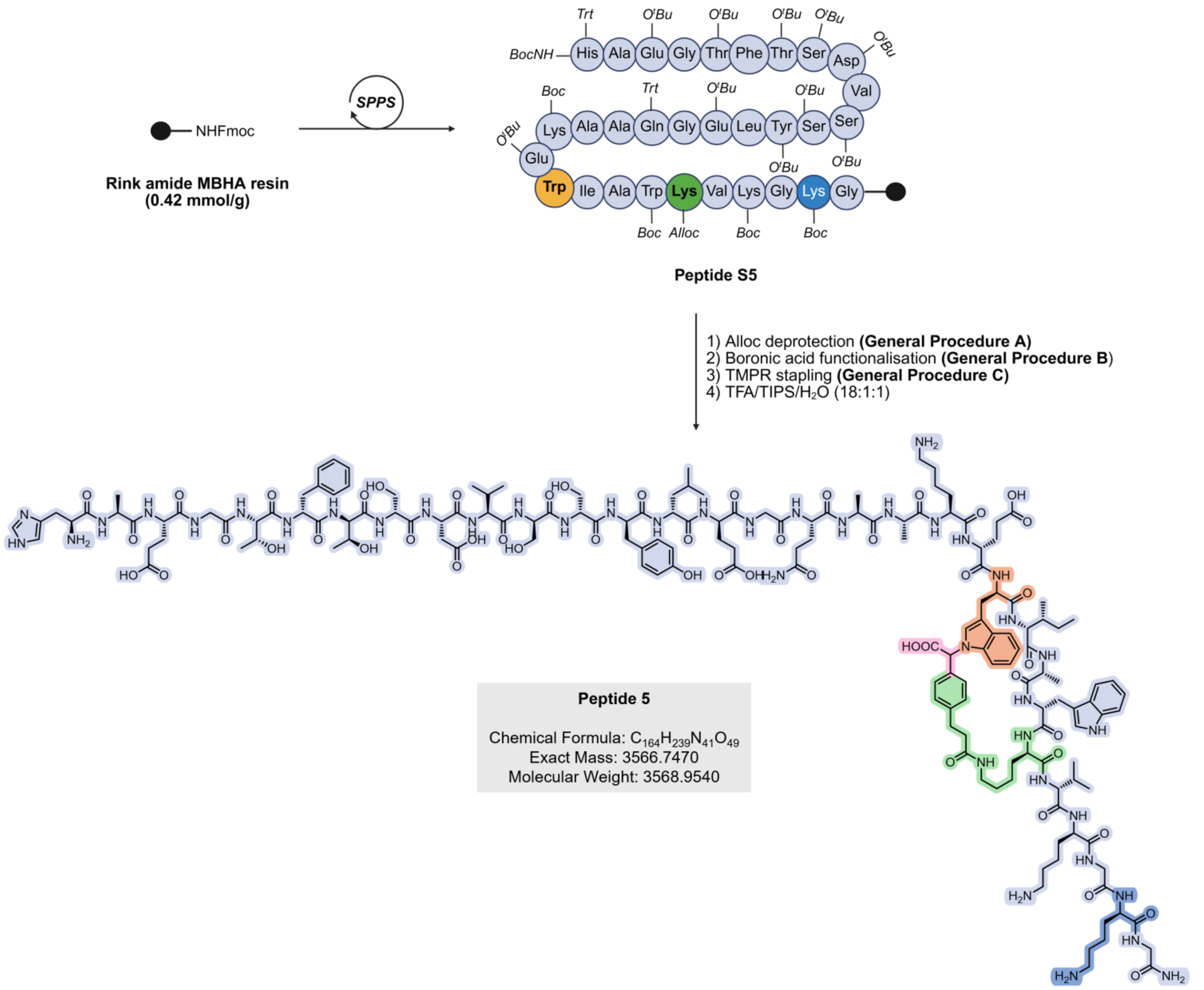

Peptide **S6** has been obtained by automated peptide synthesis, followed by deprotection of Alloc (*1.3.1. General Procedure A*), amide coupling with 4-(2 carboxyethyl)benzene boronic acid (*1.3.2. General Procedure B*) and stapled via TMPR (*1.3.3. General Procedure C*). Upon stapling, it has been cleaved from the Rink amide resin and purified via preparative HPLC to obtain compound **5** as a white solid (2.9 mg, overall yield 8%).

Analytical HPLC: R_t = 4.68 min (10–90% B over 15 min)

Purity: 98%

LRMS calcd. for $C_{164}H_{242}N_{41}O_{49}^{3+}$ *m/z* 3569.7689 $[M+3H]^{3+}$, found 1205.71 $[M/3+H+2Na]^{3+}$

- ***Peptide 6***

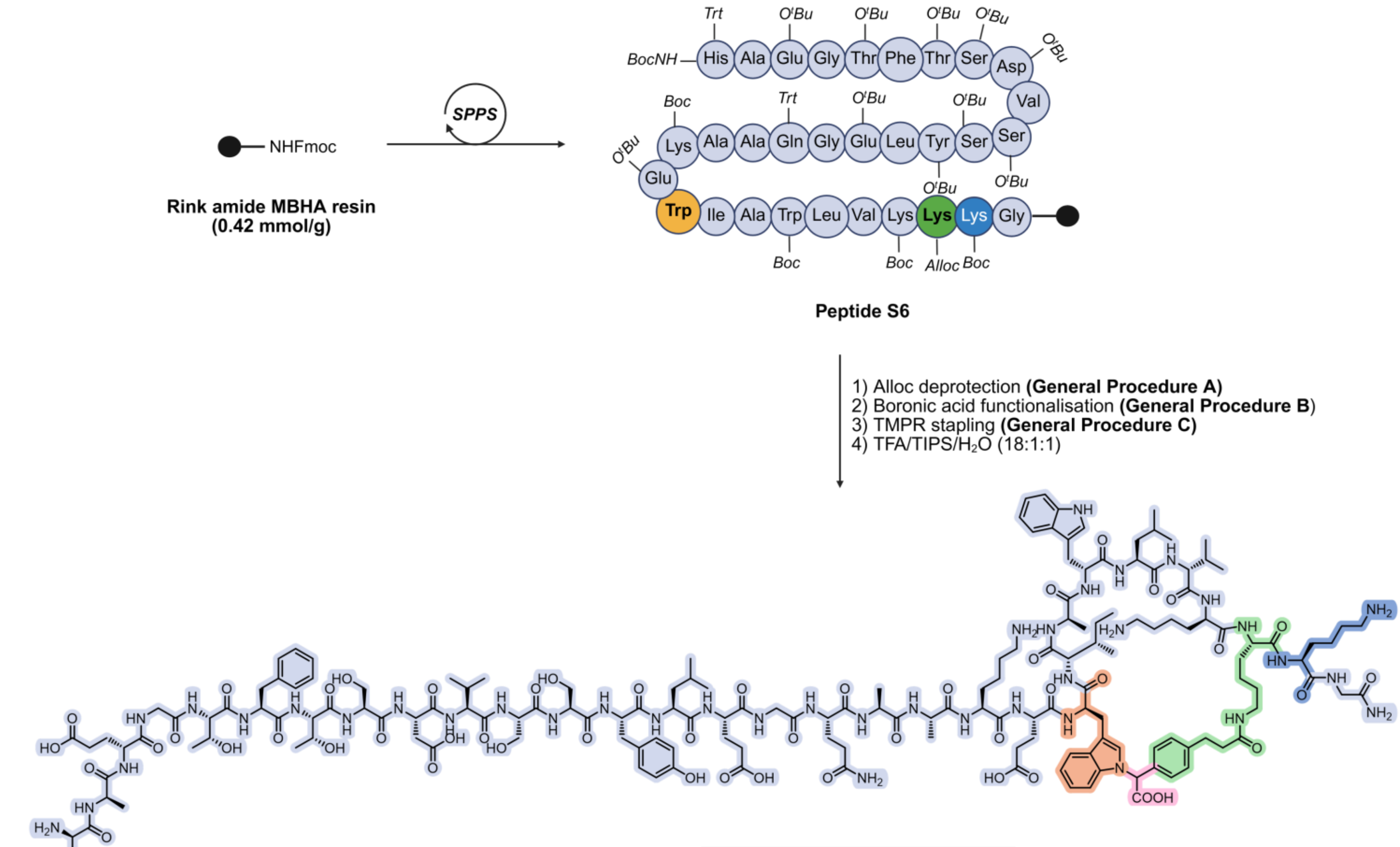

Peptide 6

Chemical Formula: $C_{168}H_{247}N_{41}O_{49}$
Exact Mass: 3622.8096
Molecular Weight: 3625.0620

Peptide **S6** has been obtained by automated peptide synthesis, followed by deprotection of Alloc (*1.3.1. General Procedure A*), amide coupling with 4-(2 carboxyethyl)benzene boronic acid (*1.3.2. General Procedure B*) and stapled via TMPR (*1.3.3. General Procedure C*). Upon stapling, it has been cleaved from the Rink amide resin and purified via preparative HPLC to obtain compound **6** as a white solid (1.26 mg, overall yield 5%).

Analytical HPLC: R_t = 5.17 min (10–90% B over 15 min)
Purity: >95%
LRMS calcd. for $C_{168}H_{250}N_{41}O_{49}^{3+}$ *m/z* 3625.8315 $[M+3H]^{3+}$, found 1224.53 $[M/3+H+2Na]^{3+}$.

▪ ***Peptide 7***

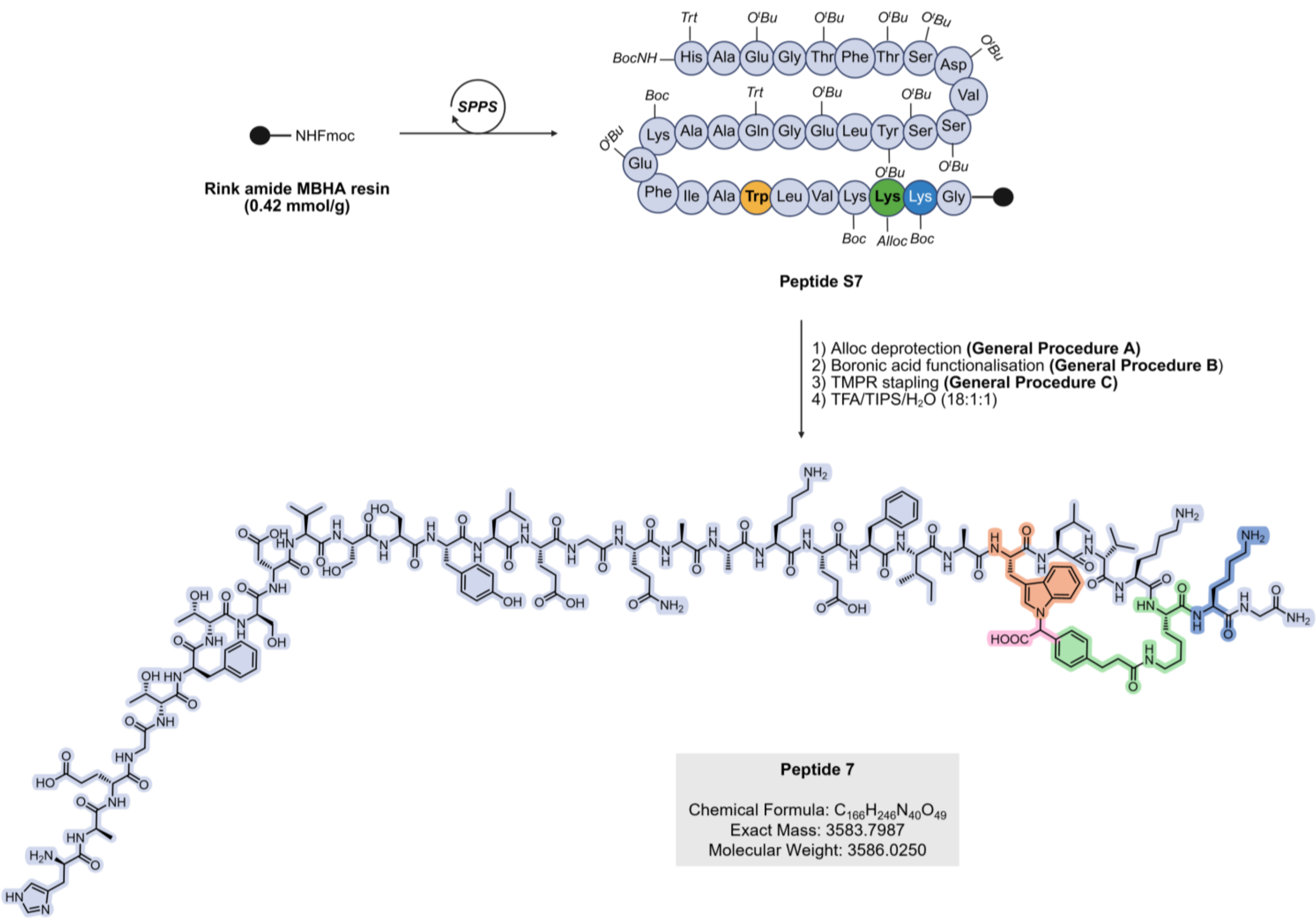

Peptide **S7** has been obtained by automated peptide synthesis, followed by deprotection of Alloc (*1.3.1. General Procedure A*), amide coupling with 4-(2 carboxyethyl)benzene boronic acid (*1.3.2. General Procedure B*) and stapled via TMPR (*1.3.3. General Procedure C*). Upon stapling, it has been cleaved from the Rink amide resin and purified via preparative HPLC to obtain compound **7** as a white solid (4.9 mg, overall yield 13%).

Analytical HPLC: R_t = 5.29 min (10–90% B over 15 min)
Purity: 96%
LRMS calcd. for $C_{168}H_{249}N_{40}O_{49}{}^{3+}$ *m/z* 3586.8206 $[M+3H]^{3+}$, found 1211.62 $[M/3+H+2Na]^{3+}$.

- ***Peptide 8***

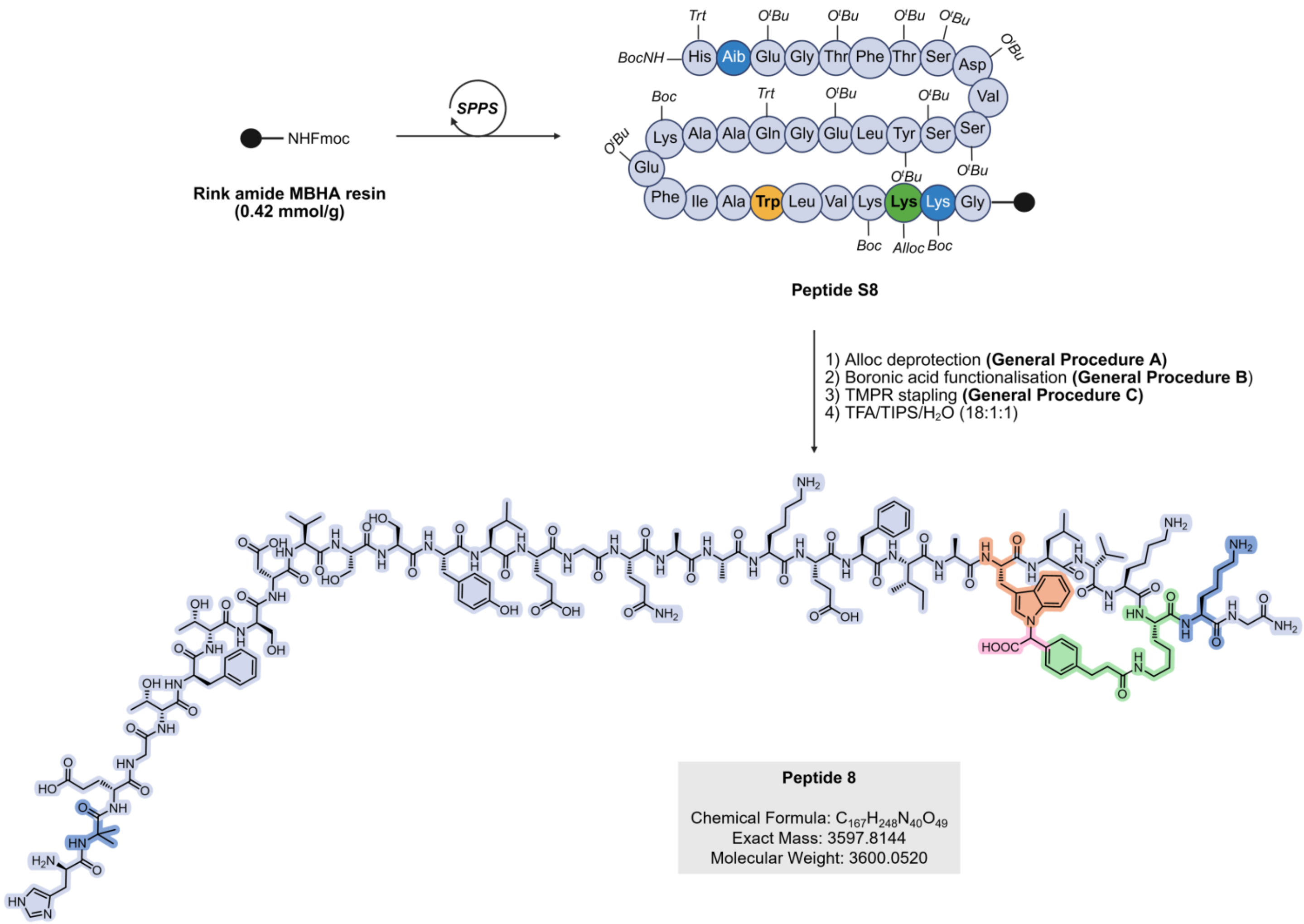

Peptide **S8** has been obtained by automated peptide synthesis, followed by deprotection of Alloc (*1.3.1. General Procedure A*), amide coupling with 4-(2 carboxyethyl)benzene boronic acid (*1.3.2. General Procedure B*) and stapled via TMPR (*1.3.3. General Procedure C*). Upon stapling, it has been cleaved from the Rink amide resin and purified via preparative HPLC to obtain compound **8** as a white solid (5.8 mg, overall yield 16%).

Analytical HPLC: R_t = 5.89 min (10–90% B over 15 min)
Purity: >95%
LRMS calcd. for $C_{167}H_{248}N_{40}O_{49}^{3+}$ *m/z* 3600.8362 $[M+3H]^{3+}$, found 1216.06 $[M/3+H+2Na]^{3+}$.

1.3.8. Synthesis of VHL-JQ1 PROTAC 9

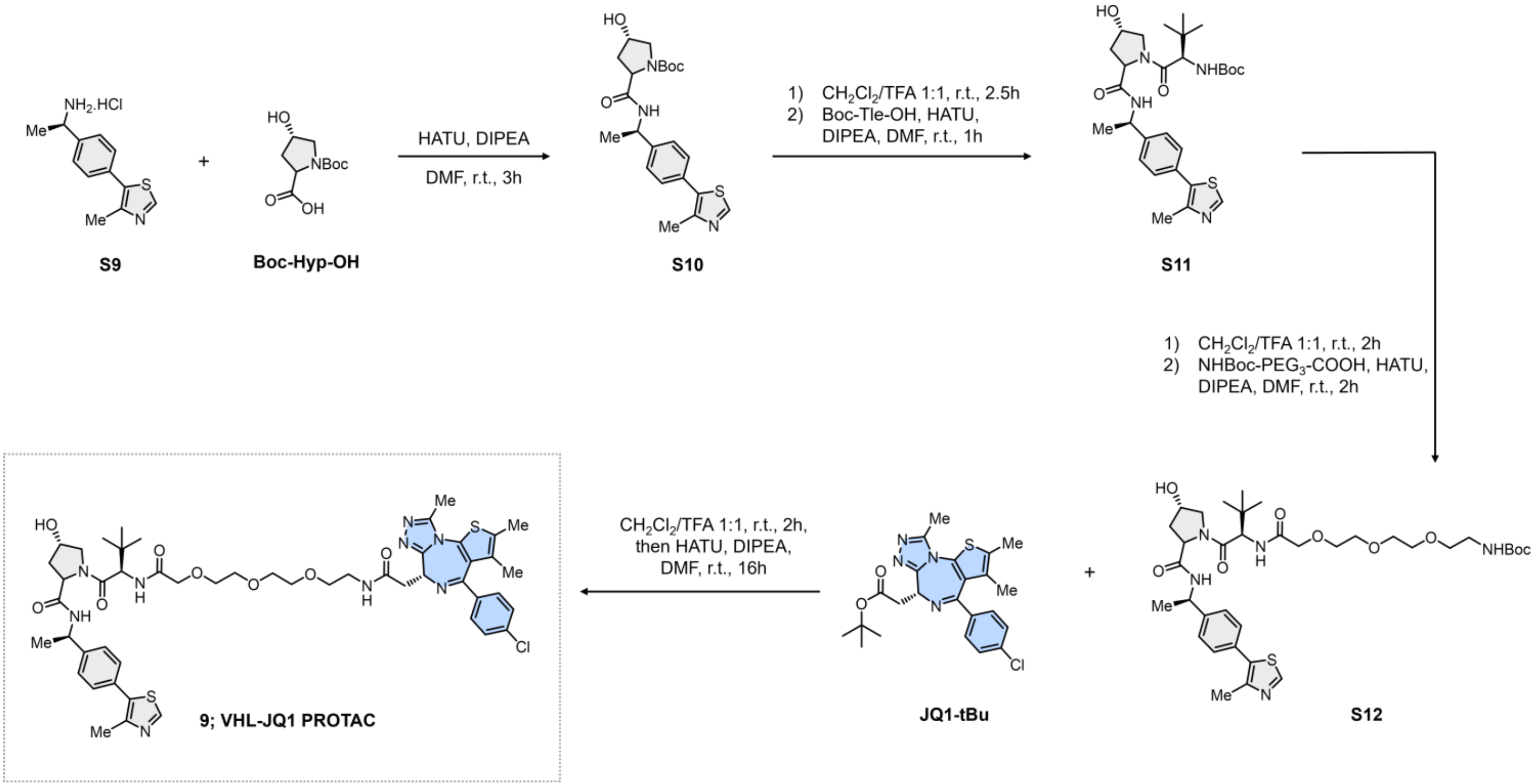

For **S10**: To a stirred solution of commercially available **S9** (1 equiv, 300 mg, 1.18 mmol) in DMF (10 mL) were added Boc-Hyp-OH (1.2 equiv, 325 mg, 1.41 mmol), HATU (1.2 equiv, 535 mg, 1.41 mmol), and DIPEA (2.4 equiv, 466 µL, 2.83 mmol). The reaction mixture was stirred at ambient temperature for 3 h, after which completion was confirmed by LC–MS analysis. The reaction was quenched by addition of EtOAc (400 mL) and extracted with brine (3 × 400 mL) and sat. aqueous NH_4Cl (3 × 400 mL). The organic layer was dried over $MgSO_4$, filtered, and concentrated under reduced pressure to afford crude **S10** as a yellow solid (500 mg, 1.16 mmol, 98% yield), which was used in the next step without further purification.

UV LCMS R_t = 1.99 min (5–95% B over 5 min)
Purity: 90%
LRMS calcd. for $C_{22}H_{30}N_3O_4S^+$ *m/z* 432.1952 $[M+H]^+$, found 432.40.

For **S11**: To a stirred solution of **S10** (1 equiv, 500 mg, 1.16 mmol) in CH_2Cl_2 (15 mL) was added trifluoroacetic acid (15 mL) and the reaction mixture was stirred at ambient temperature for 2.5 h, after which Boc deprotection was confirmed by LC–MS analysis. The reaction mixture was blowed down under a stream of nitrogen and re-dissolved in DMF (15 mL). To a stirred solution, DIPEA has been added until the pH of the reaction mixture has been adjusted to pH 7–8, using a pH paper (usually 10–12 mL). Then, Boc-Tle-OH (1.2 equiv, 321 mg, 1.39 mmol), HATU (1.2 equiv, 528 mg, 1.39 mmol), and additional DIPEA (2.4 equiv, 459 µL, 2.78 mmol) were added and the reaction mixture was stirred for 1 h, after which completion was confirmed by LC–MS analysis. The reaction was quenched by addition of EtOAc (400 mL) and extracted with brine (3 × 300 mL), sat. aqueous $NaHCO_3$ (1 × 300 mL) and sat. aqueous NH_4Cl (1 × 300 mL). The organic layer was dried over $MgSO_4$, filtered, and concentrated under reduced pressure to afford crude **S11** as an off-white fluffy solid (602 mg, 1.10 mmol, 95% yield), which was used in the next step without further purification.

UV LCMS R_t = 2.22 min (5–95% B over 5 min)

Purity: >95%

LRMS calcd. for $C_{28}H_{41}N_4O_5S^+$ *m/z* 545.2792 [M+2H]$^+$, found 545.41.

For **S12**: To a stirred solution of **S11** (1 equiv, 200 mg, 0.36 mmol) in CH_2Cl_2 (20 mL) was added trifluoroacetic acid (20 mL) and the reaction mixture was stirred at ambient temperature for 2 h, after which Boc deprotection was confirmed by LC–MS analysis. The reaction mixture was blowed down under a stream of nitrogen and re-dissolved in DMF (20 mL). To a stirred solution, DIPEA has been added until the pH of the reaction mixture has been adjusted to pH 7–8, using a pH paper (usually 10–12 mL). Then, BocNH-PEG$_3$-COOH (1.2 equiv, 135 mg, 0.44 mmol), HATU (1.2 equiv, 167 mg, 0.44 mmol), and additional DIPEA (2.4 equiv, 142 µL, 0.86 mmol) were added and the reaction mixture was stirred for 2 hours, after which completion was confirmed by LC–MS analysis. The reaction was quenched by addition of EtOAc (400 mL) and extracted with brine (2 × 300 mL), sat. aqueous $NaHCO_3$ (2 × 300 mL) and sat. aqueous NH_4Cl (3 × 300 mL). The organic layer was dried over $MgSO_4$, filtered, and concentrated under reduced pressure to afford crude **S12** as a brown oil (213 mg, 0.29 mmol, 81% yield), which was used in the next step without further purification.

UV LCMS R_t = 2.16 min (5–95% B over 5 min)

Purity: >95%

LRMS calcd. for $C_{36}H_{56}N_5O_9S^+$ *m/z* 734.3793 [M+H]$^+$, found 734.50.

For **9**: Boc deprotection of **S12** (1 equiv, 158 mg, 0.21 mmol) and *tert*-butyl deprotection of commercially available **JQ1-tBu** (1.5 equiv, 147 mg, 0.32 mmol) was carried out separately by treatment with trifluoroacetic acid in CH_2Cl_2 (1:1, 20 mL) at ambient temperature for 2 h; complete deprotections were confirmed by LC–MS analysis. Both reaction mixtures were blowed down under a stream of nitrogen and re-dissolved in DMF (3 mL, each). To these stirred solutions, DIPEA has been added until the pH has been adjusted to pH 7–8, using a pH paper (usually 3 mL, each). Then, using plastic Pasteur pipette, crude JQ1 has been added to a flask with deprotected **S12**, followed by addition of HATU (1.2 equiv, 98 mg, 0.26 mmol), and additional DIPEA (2.5 equiv, 90 µL, 0.54 mmol). The reaction mixture was stirred for 16 hours, after which completion was confirmed by LC–MS analysis. The reaction was quenched by addition of EtOAc (200 mL), extracted with brine (2 × 200 mL) and sat. aqueous NH_4Cl (2 × 200 mL). The organic layer was dried over $MgSO_4$, filtered, and concentrated under reduced pressure and the crude product was purified by preparative HPLC (5–95% B) to obtain **9** as a brown oil (113 mg, 0.11 mmol, 53% yield).

Analytical HPLC R_t = 10.39 min (5–95% B over 18 min)

Purity: >99%

HRMS calcd. for $C_{50}H_{64}ClN_9O_8S_2^{2+}$ *m/z* 1017.3997 [M+2H]$^{2+}$, found 1017.4004.

^{1}H NMR (500 MHz, DMSO-d_6): δ 8.97 (s, 1H), 8.42 (d, *J* = 7.7 Hz, 1H), 8.28 (t, *J* = 5.7 Hz, 1H), 7.49 – 7.33 (m, 10H), 4.88 (p, *J* = 7.1 Hz, 1H), 4.55 – 4.47 (m, 2H), 4.43 (t, *J* = 8.2 Hz, 1H), 4.28 – 4.25 (m, 1H), 3.95 (s, 2H),

3.62 – 3.54 (m, 12H, *overlap with solvent*), 3.31 – 3.17 (m, 6H, *overlap with solvent*), 2.58 (s, 3H), 2.43 (s, 3H), 2.39 (s, 3H), 1.60 (s, 3H), 1.35 (d, J = 7.0 Hz, 3H), 0.92 (s, 9H) ppm.
^{13}C NMR (126 MHz, DMSO-d_6): δ 170.9, 170.1, 169.4, 169.0, 163.5, 155.5, 151.9, 150.3, 148.1, 145.1, 137.1, 135.7, 132.6, 131.6, 131.2, 130.6, 130.3, 130.0, 130.0, 129.2, 128.9, 126.7, 70.8, 70.2, 70.0, 70.0, 69.6, 69.2, 60.2, 59.0, 56.9, 56.1, 55.3, 54.2, 48.2, 40.8, 39.1, 38.1, 37.8, 36.1, 26.6, 22.9, 16.4, 16.3, 14.4, 13.1, 11.7 ppm.

1.3.9. Synthesis of linear alkyne-acid linker 10

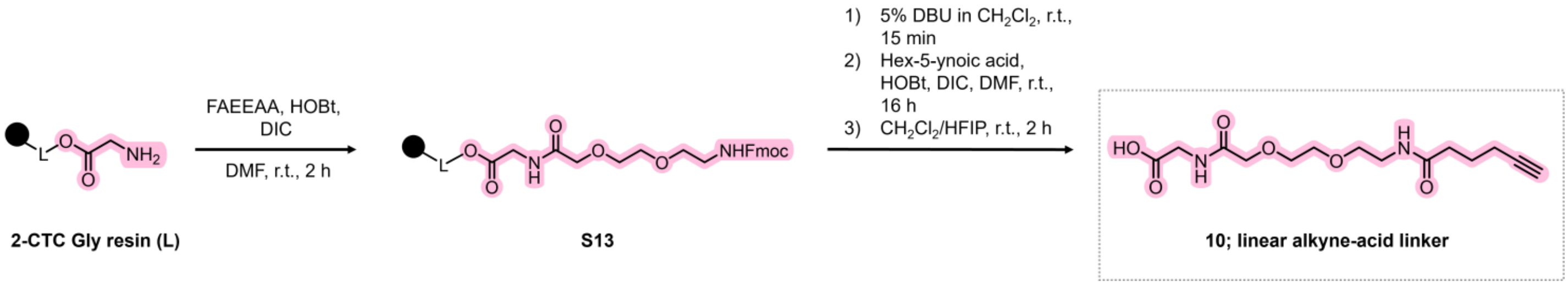

Cmpd.	Carboxylic acid used	CAS (carboxylic acid)	MW	Conc. [M]	n [mmol]	m [mg]	V [μL]
**S13**	{2-[2-(Fmoc-amino)ethoxy]ethoxy}acetic acid (FAEEAA)	166108-71-0	380	0.2	1	380	-
**10**	Hex-5-ynoic acid	53293-00-8	112	0.2	1	-	110

2-chlorotrityl (2-CTC) Gly resin scale 500 mg (H-Gly-OH 2-CTC, loading 0.84 mmol/g). Compounds have been prepared according to the *1.3.4. General Procedure D: Manual Fmoc deprotection on resin* and *1.3.5. General Procedure E: Manual amide coupling on resin.* For all amide couplings have been used HOBt (0.2 M; 1 mmol, 153 mg), DIC (0.2 M; 1 mmol, 153 μL), and DMF (5 mL). Final cleavage have been done using CH_2Cl_2/HFIP (4:1 v/v, 20 mL) for 2 hours, upon which the crude product **10** has been concentrated under reduced pressure and purified using preparative MDAP HPLC (5–95% B). Overall yield of **10** from H-Gly-OH 2-CTC: 134 mg, 0.43 mmol, quantitative overall yield.

^{1}H NMR (500 MHz, $CDCl_3$) δ 8.08 (br s, 1H), 7.48 (t, J = 5.2 Hz, 1H), 6.87 (t, J = 5.7 Hz, 1H), 4.11 – 4.07 (m, 2H), 4.04 (s, 2H), 3.72 – 3.67 (m, 2H), 3.65 – 3.61 (m, 2H), 3.56 (t, J = 5.0 Hz, 2H), 3.47 – 3.41 (m, 2H), 2.37 – 2.32 (m, 2H), 2.21 (tt, J = 6.5, 2.6 Hz, 2H), 1.99 (t, J = 2.6 Hz, 1H), 1.82 (p, J = 7.2 Hz, 2H) ppm.
^{13}C NMR (126 MHz, $CDCl_3$) δ 174.0, 171.6, 171.0, 170.9, 83.5, 71.2, 71.2, 70.2, 70.1, 69.4, 40.9, 39.5, 35.0, 24.5, 17.9 ppm.
HRMS calcd. for $C_{14}H_{23}N_2O_6^+$ m/z 315.1551 [M+H]$^+$, found 315.1566.

1.3.10. Synthesis of branched alkyne-acid linker 13

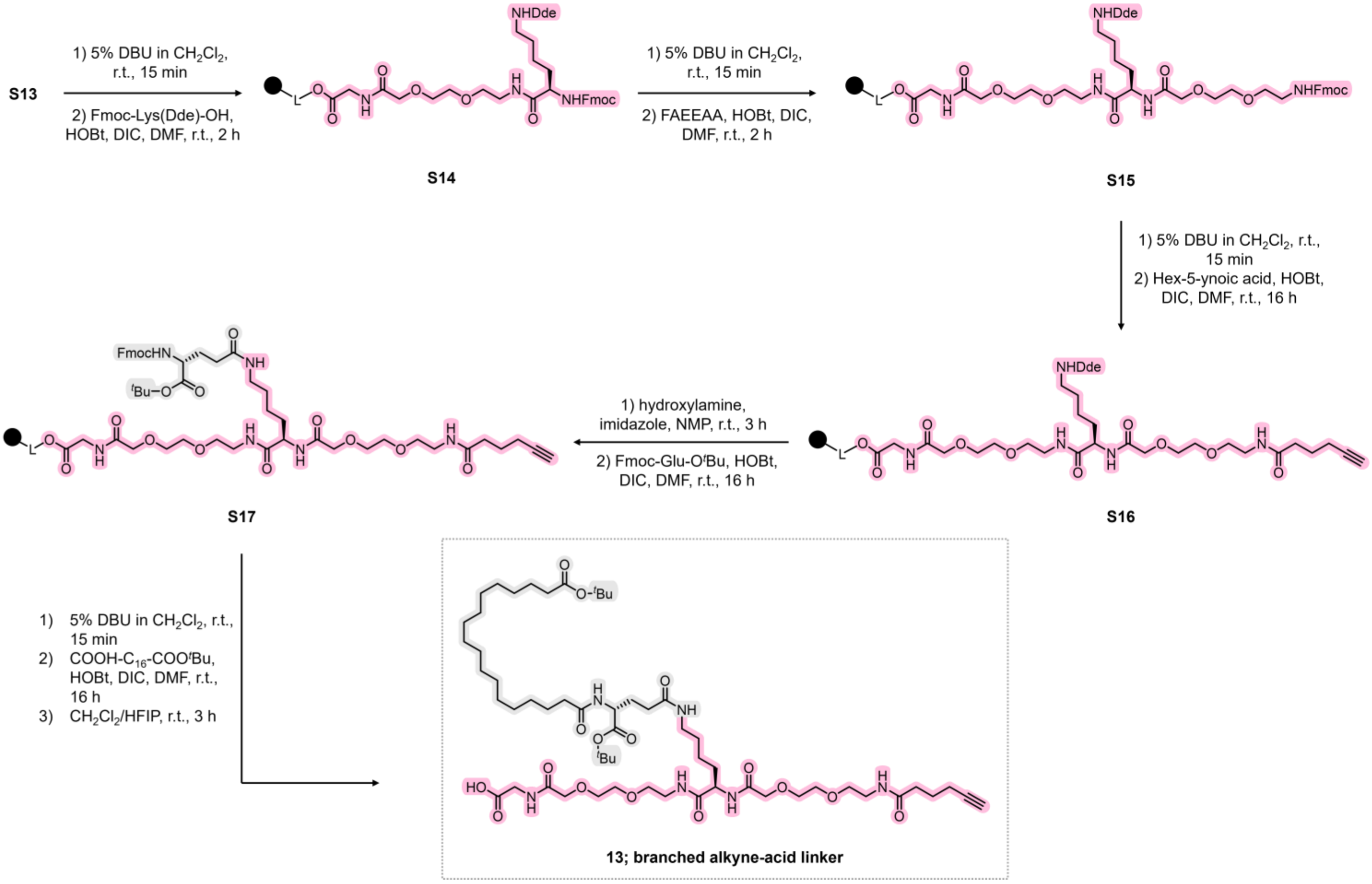

Cmpd.	Carboxylic acid used	CAS (carboxylic acid)	MW	Conc. [M]	n [mmol]	m [mg]	V [mL]
**S14**	Fmoc-Lys(Dde)-OH	150629-67-7	532	0.2	1	532	-
**S15**	{2-[2-(Fmoc-amino)ethoxy]ethoxy}acetic acid (FAEEAA)	166108-71-0	380	0.2	1	380	-
**S16**	Hex-5-ynoic acid	53293-00-8	112	0.2	1	-	110
**S17**	Fmoc-Glu-OtBu	84793-07-7	425	0.2	1	425	-
**13**	18-(tert-Butoxy)-18-oxooctadecanoic acid	843666-40-0	370	0.2	1	370	-

2-chlorotrityl (2-CTC) Gly resin scale 500 mg (H-Gly-OH 2-CTC, loading 0.84 mmol/g). Compounds have been prepared according to the *1.3.4. General Procedure D: Manual Fmoc deprotection on resin* and *1.3.5. General Procedure E: Manual amide coupling on resin.* For all amide couplings have been used HOBt (0.2 M; 1 mmol, 153 mg), DIC (0.2 M; 1 mmol, 153 µL), and DMF (5 mL). Dde protecting group has been deprotected using hydroxylamine (0.2 M; 1 mmol, 69 mg) and imidazole (0.2 M; 1 mmol, 68 mg) in NMP (5 mL) over 3 hours. Final cleavage have been done using CH_2Cl_2/HFIP (4:1 v/v, 20 mL) for 2 hours, upon which the crude product **13** has been concentrated under reduced pressure and purified using preparative HPLC (80–100% B). Overall yield of **13** from H-Gly-OH 2-CTC: 33 mg, 0.029 mmol, 7% overall yield.

^{1}H NMR (500 MHz, $CDCl_3$) δ 7.75 (d, *J* = 9.0 Hz, 1H), 7.59 – 7.48 (m, 3H), 7.00 (t, *J* = 5.6 Hz, 1H), 6.41 (d, *J* = 7.7 Hz, 1H), 4.75 – 4.73 (m, 1H), 4.40 – 4.35 (m, 1H), 4.23 – 4.20 (m, 1H), 4.07 – 3.99 (m, 5H), 3.76 – 3.14 (m, 19H), 2.39 – 2.32 (m, 2H), 2.28 – 2.09 (m, 9H), 1.94 (t, *J* = 2.7 Hz, 1H), 1.89 – 1.80 (m, 4H), 1.66 – 1.51 (m, 7H), 1.46 (s, 9H), 1.43 (s, 9H), 1.33 – 1.20 (m, 25H) ppm.
^{13}C NMR (126 MHz, $CDCl_3$) δ 174.2, 173.5, 173.1, 173.0, 171.7, 171.2, 170.6, 170.3, 84.0, 82.9, 80.0, 71.3, 70.7, 70.4, 70.3, 70.2, 70.0, 69.9, 68.9, 52.3, 52.2, 39.5, 39.3, 39.0, 36.8, 35.7, 35.0, 33.0, 32.8, 29.8, 29.7, 29.7, 29.6, 29.6, 29.4, 29.4, 29.4, 29.2, 29.0, 28.2, 28.1, 25.8, 25.2, 24.5, 22.7, 18.1 ppm.
HRMS calcd. for $C_{57}H_{101}N_6O_{16}^+$ *m/z* 1125.7269 $[M+H]^+$, found 1125.7274.

1.3.11. Synthesis of linear VHL-JQ1 alkyne-ester 11

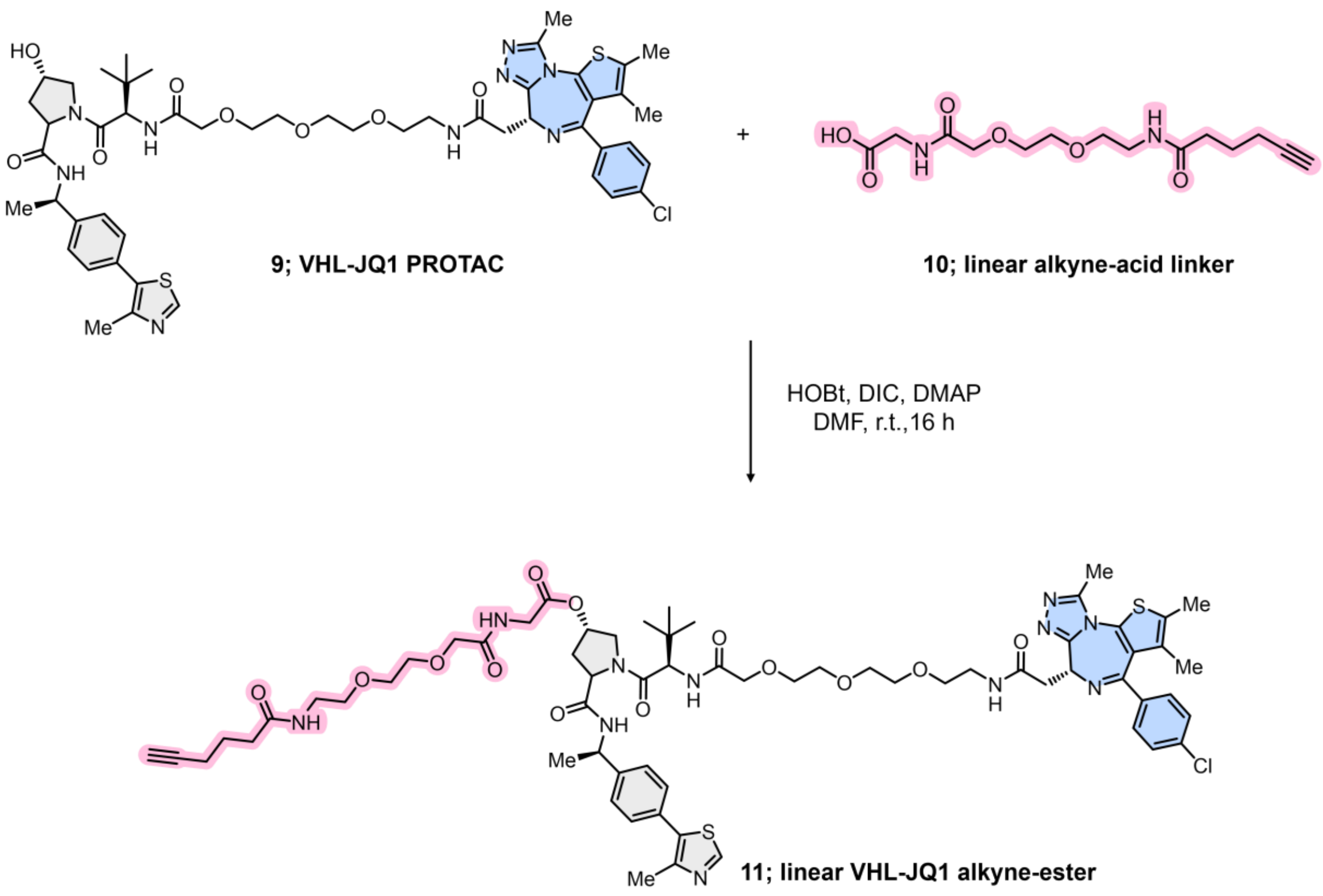

To a stirred solution of **9** (1 equiv, 20 mg, 0.019 mmol) in DMF (2 mL) were added **10** (5 equiv, 30 mg, 0.098 mmol), HOBt (5 equiv, 15 mg, 0.098 mmol) and DMAP (5 equiv, 12 mg, 0.098 mmol), followed by addition of DIC (5 equiv, 15 µL, 0.098 mmol). The reaction mixture was stirred at ambient temperature for 16 h, after which completion was confirmed by LC–MS analysis. The crude product was taken directly to the preparative HPLC and purified (20–95% B), affording compound **11** as an off-white solid (15 mg, 0.011 mmol, 60% yield).

Analytical HPLC R_t = 11.19 min (5–95% B over 18 min)

Purity: >99%

HRMS calcd. for $C_{64}H_{83}ClN_{11}O_{13}S_2^+$ *m/z* 1312.5296 $[M+H]^+$, found 1312.5294.

1.3.12. Synthesis of branched VHL-JQ1 alkyne-ester 14

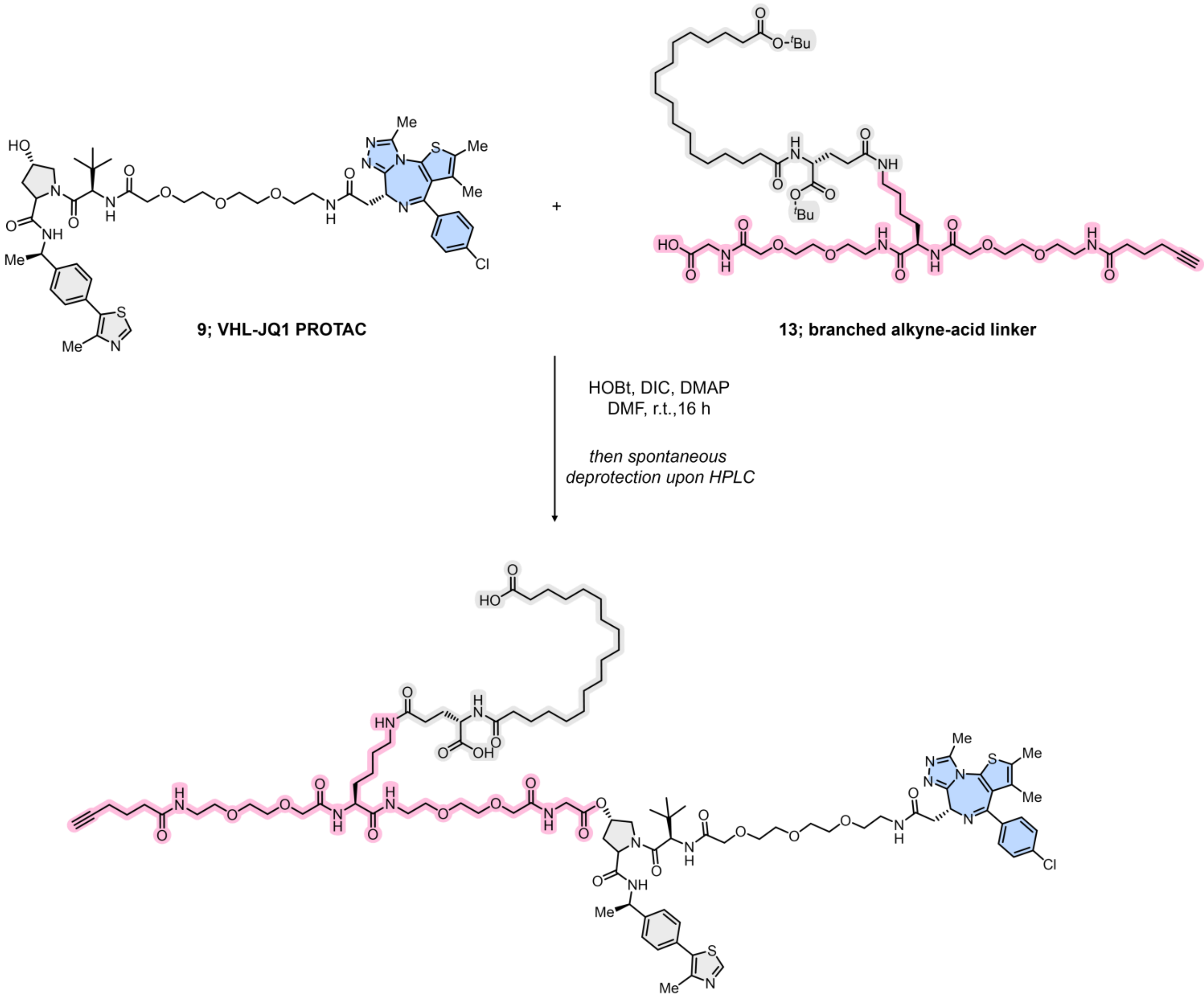

14; branched VHL-JQ1 alkyne-ester

To a stirred solution of **9** (1 equiv, 3 mg, 0.0026 mmol) in DMF (500 µL) were added **13** (5 equiv, 15 mg, 0.013 mmol), HOBt (25 equiv, 10 mg, 0.065 mmol) and DMAP (30 equiv, 10 mg, 0.081 mmol), followed by addition of DIC (25 equiv, 10 µL, 0.065 mmol). The reaction mixture was stirred at ambient temperature for 16 h, after which completion was confirmed by LC–MS analysis. The crude product was taken directly to the preparative HPLC (80–100% B) and purified, affording compound **11** as an off-white solid (5 mg, 0.0024 mmol, 96% yield). *Note: due to the residual acidic environment upon purification, spontaneous deprotection of tert-butyl protecting groups has been observed.*

Analytical HPLC R_t = 9.78 min (40–95% B over 18 min)

Purity: 93%

HRMS calcd. for $C_{99}H_{146}ClN_{15}O_{23}S_2^{2+}$ *m/z* 2011.9835 $[M+2H]^{2+}$, found 2011.9900.

1.3.13. Synthesis of GLP1-TMPR-azide 12

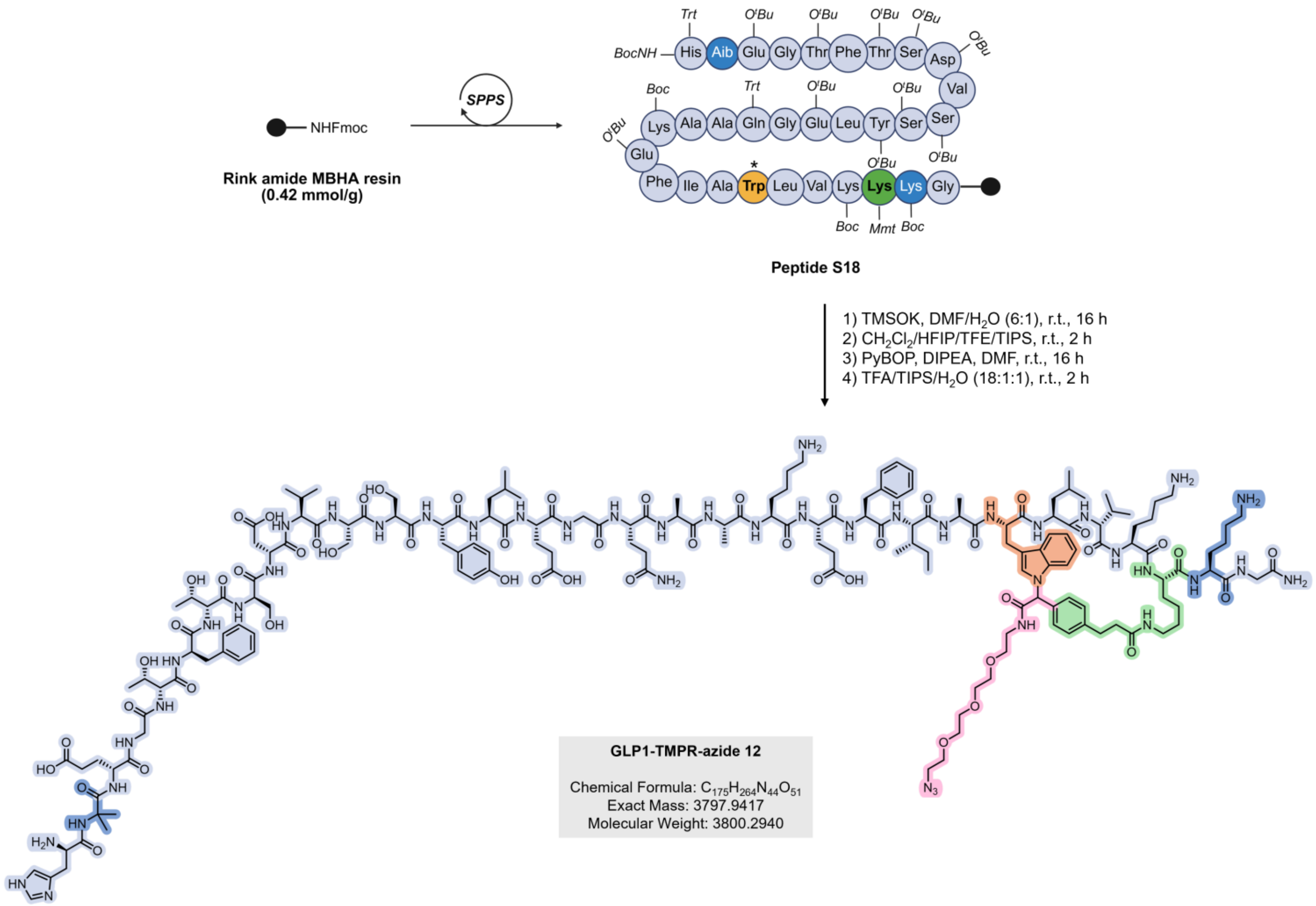

Peptide **S18** has been obtained by manual peptide synthesis, using Rink Amide MBHA resin (scale 300 mg, 0.42 mmol/g). Compounds have been prepared according to the *1.3.4. General Procedure D: Manual Fmoc deprotection on resin* and *1.3.5. General Procedure E: Manual amide coupling on resin.* For all amide couplings have been used HOBt (0.2 M; 1 mmol, 153 mg), DIC (0.2 M; 1 mmol, 153 µL), and DMF (5 mL). Upon peptide synthesis, methoxy group on modified tryptophan **S20** (synthesis below) has been hydrolysed (16 h) on resin using TMSOK (0.2 M; 1.2 mmol, 153 mg) and DMF/H_2O (6:1 v/v, 7 mL), followed by Mmt deprotection (2 h) using CH_2Cl_2/HFIP/TFE/TIPS 13:4:2:1 (10 mL) and intramolecular cyclisation (16 h) using PyBOP (0.2 M; 1 mmol, 520 mg) and DIPEA (0.4 M; 2 mmol, 330 µL) in DMF (5 mL). Final cleavage of he peptide was done using TFA/TIPS/H_2O (18:1:1) for 2 h, upon which the crude product **12** has been purified using preparative MDAP HPLC (5–95% B). Overall yield of **12**: 21.15 mg, 0.005 mmol, 4.5% overall yield. <u>Note:</u> *Amide coupling upon TMPR stapling on resin with NH_2–PEG$_3$–N_3 gave reduced yields on larger resin scales, most likely owing to instability of the NHS ester on the resin, therefore an alternative approach was pursued using pre-modified **S20**.*

Analytical HPLC R_t = 9.03 min (5–95% B over 18 min)

Purity: >95%

HRMS calcd. for $C_{175}H_{267}N_{44}O_{51}^{3+}$ *m/z* 3800.9635 $[M+3H]^{3+}$, found 950.9947 $[M/4+3H]^{3+}$

Synthesis of Trp* S20

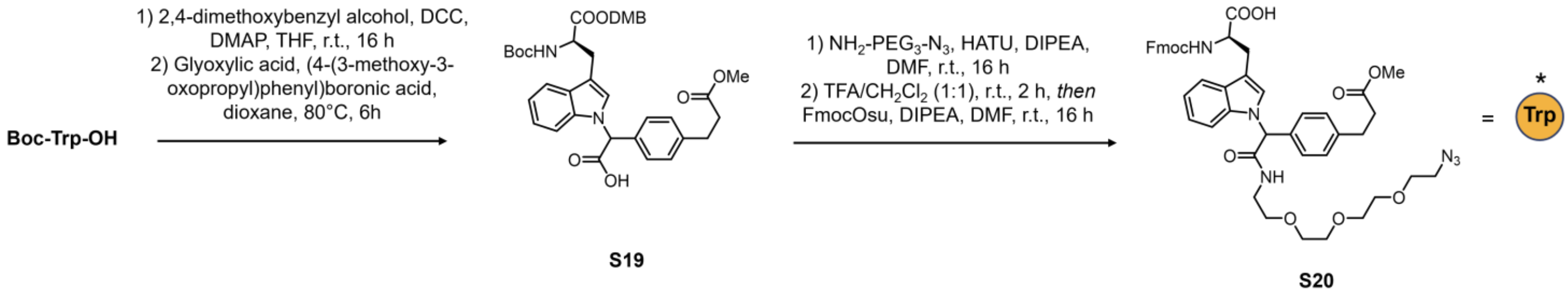

For **S19**: To a stirred solution of commercially available Boc-Trp-OH (1 equiv, 1 g, 3.28 mmol) in THF (15 mL) was added 2,4-dimethoxybenzyl alcohol (DBMOH) (1.3 equiv, 715 mg, 4.27 mmol), DCC (1.1 equiv, 745 mg, 3.61 mmol) and DMAP (0.1 equiv, 40 mg, 0.32 mmol), and the reaction mixture was stirred at ambient temperature for 4 h, upon which completion was confirmed by LC–MS analysis. The reaction was quenched by addition of EtOAc (200 mL) and extracted with sat. aqueous NH_4Cl (3 × 200 mL). The organic layer was dried over $MgSO_4$, filtered, and concentrated under reduced pressure to afford Boc-Trp-ODMB as a white solid (1.46 g, 3.21 mmol), which was subsequently re-dissolved in dioxane (30 mL), followed by addition of glyoxylic acid (1.3 equiv, 384 mg, 4.18 mmol) and (4-(3-methoxy-3-oxopropyl)phenyl)boronic acid (1.3 equiv, 870 mg, 4.18 mmol). The reaction mixture was heated up to 80°C and stirred for 16 h, upon which completion was confirmed by LC–MS analysis. The reaction was cooled down and quenched by addition of EtOAc (200 mL) and extracted with sat. aqueous NH_4Cl (3 × 200 mL) and brine (2 × 200 mL). The organic layer was dried over $MgSO_4$, filtered, and concentrated under reduced pressure. Crude product was purified by column chromatography (CH_2Cl_2/MeOH 20:1 to 4:1, v/v) to afford **S19** as a brown solid (937 mg, 1.39 mmol, 42% over 2 steps).

For **S20**: **S19** (1 equiv, 637 mg, 0.94 mmol) was then dissolved in DMF (10 mL) and NH_2-PEG_3-N_3 (1.5 equiv, 280 µL, 1.41 mmol), HATU (1.5 equiv, 535 mg, 1.5 mmol) and DIPEA (3 equiv, 467 µL, 2.83 mmol) were added. The reaction mixture was stirred at ambient temperature for 16 hours, upon which completion was confirmed by LC–MS analysis. The reaction was cooled down and quenched by addition of EtOAc (200 mL) and extracted with sat. aqueous NH_4Cl (3 × 200 mL). The organic layer was dried over $MgSO_4$, filtered, concentrated under reduced pressure to dryness and re-dissolved in CH_2Cl_2 (20 mL). Trifluoroacetic acid (20 mL) has been added and the reaction mixture has been stirred at ambient temperature for 2 hours. Once the LC-MS analysis confirmed full conversion to the deprotected product, the reaction mixture was blowed down under a stream of nitrogen and re-dissolved in DMF (20 mL). To a stirred solution, DIPEA has been added until the pH of the reaction mixture has been adjusted to pH 7–8, using a pH paper (usually 10–12 mL). Then, Fmoc-Osu (1.5 equiv, 475 mg, 1.41 mmol) was added and the reaction mixture was stirred for 16 hours, after which completion was confirmed by LC–MS analysis. The reaction was quenched by addition of EtOAc (400 mL) and extracted with brine (5 × 300 mL), and sat. aqueous NH_4Cl (5 × 300 mL). The organic layer was dried over $MgSO_4$, filtered, and concentrated under reduced pressure. Crude product was purified by column chromatography (CH_2Cl_2/MeOH 20:1 to 4:1, v/v) to afford pure **S20** as a brown solid (644 mg, 0.76 mmol, 80% yield over 2 steps).

UV LCMS R_t = 2.88 min (5–95% B over 5 min)

Purity: >95%

LRMS calcd. for $C_{46}H_{49}N_6O_{10}^-$ *m/z* 845.3516 [M-H]$^-$, found 845.71.

^{1}H NMR (700 MHz, DMSO) δ 8.60 (t, *J* = 5.7 Hz, 1H), 7.88 (d, *J* = 7.6 Hz, 2H), 7.67 (ddd, *J* = 28.4, 14.8, 7.3 Hz, 3H), 7.60 (dd, *J* = 8.0, 2.6 Hz, 1H), 7.42 – 7.38 (m, 3H), 7.33 – 7.24 (m, 3H), 7.19 – 7.08 (m, 6H), 7.05 (t, *J* = 7.4 Hz, 1H), 6.30 (app d, *J* = 5.6 Hz, 1H), 4.25 – 4.11 (m, 4H), 3.58 – 3.52 (m, 7H, *overlap with solvent*), 3.52 – 3.48 (m, 7fH, *overlap with solvent*), 3.36 – 3.34 (m, 3H), 3.31 – 3.22 (m, 3H), 3.17 (ddd, *J* = 15.2, 9.7, 6.0 Hz, 1H), 3.01 (ddd, *J* = 15.0, 9.8, 6.1 Hz, 1H), 2.73 (dt, *J* = 16.3, 7.7 Hz, 2H), 2.53 (app ft, *J* = 7.7 Hz, 1H) ppm.

^{13}C NMR (176 MHz, DMSO) δ 173.6, 172.5, 168.3, 156.0, 143.8, 143.7, 143.7, 140.7, 140.6, 140.4, 140.3, 136.4, 136.4, 134.7, 134.7, 128.4, 128.3, 127.6, 127.6, 127.6, 127.1, 126.0, 125.7, 125.4, 125.3, 125.3, 125.3, 121.3, 120.1, 119.2, 119.2, 118.6, 118.5, 110.4, 109.8, 69.7, 69.7, 69.6, 69.6, 69.2, 68.8, 65.7, 60.8, 60.7, 54.9, 54.8, 51.3, 51.3, 50.0, 46.6, 46.6, 38.8, 34.5, 34.5, 29.7, 26.9 ppm.

1.3.14. Synthesis of GLP1-PROTAC 1

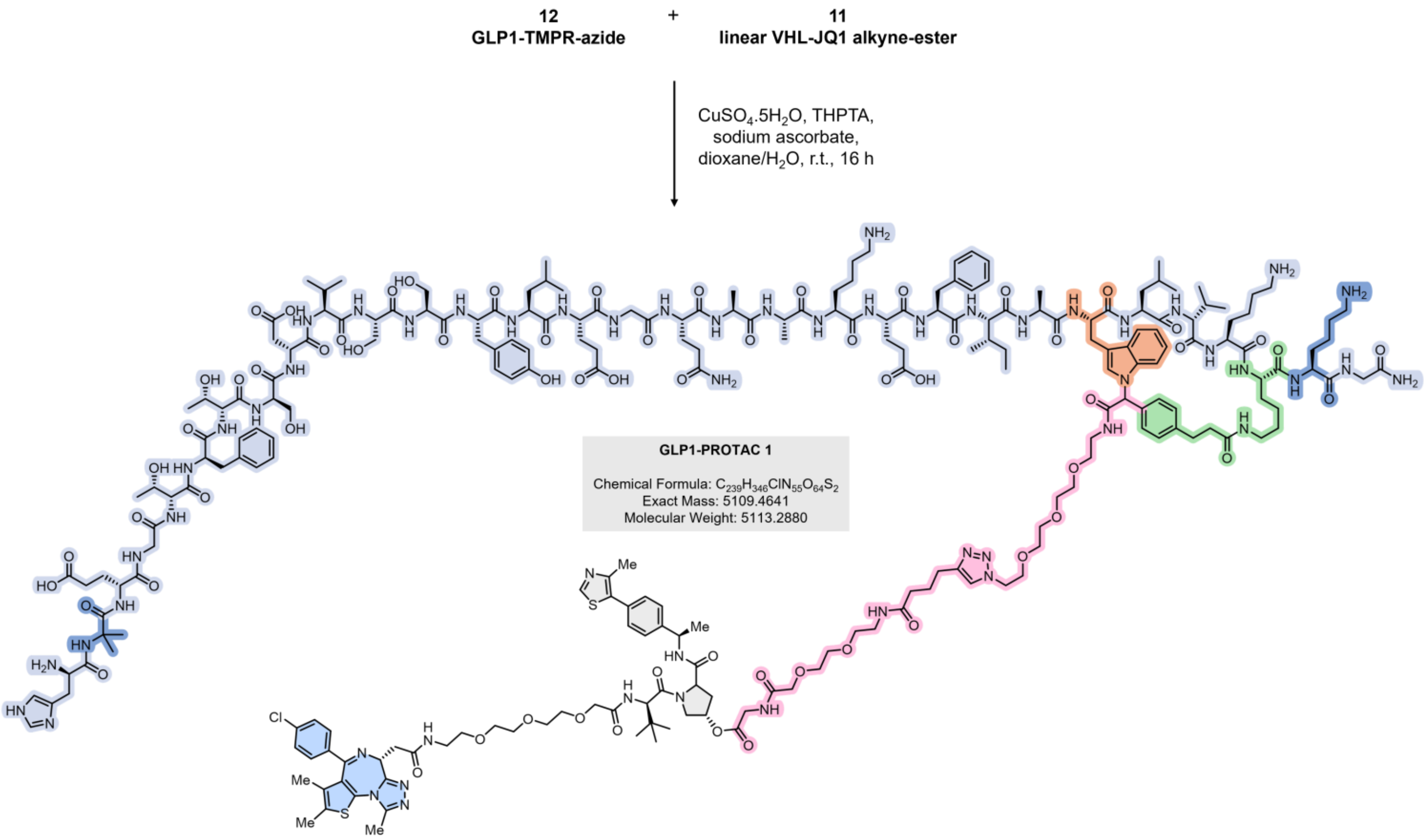

To a stirred solution of **12** (1 equiv, 10 mg, 0.0026 mmol) in dioxane/H_2O (1:1, 2 mL, v/v) was added **11** (1 equiv, 3.5 mg, 0.0026 mmol), $CuSO_4.5H_2O$ (8 equiv, 5 mg, 0.020 mmol), THPTA (4 equiv, 5 mg, 0.011 mmol) and sodium ascorbate (10 equiv, 5 mg, 0.025 mmol). The reaction mixture was stirred at ambient temperature for 16 h, after which completion was confirmed by LC–MS analysis. The crude product was evaporated to dryness, dissolved in MeCN/H_2O (1:1) and purified by preparative HPLC (20–95% B), affording compound **GLP1-PROTAC 1** as an off-white solid (3.23 mg, 0.00063 mmol, 24% yield).

Analytical HPLC R_t = 8.97 min (20–80% B over 18 min)

Purity: >95%

HRMS calcd. for $C_{239}H_{347}ClN_{55}O_{64}S_2^+$ *m/z* 5110.4713 [M+H]$^+$, found 1023.4992 [M/5+H]$^+$

1.3.15. Synthesis of GLP1-PROTAC 2

12 **GLP1-TMPR-azide** + **14** **branched VHL-JQ1 alkyne-ester**

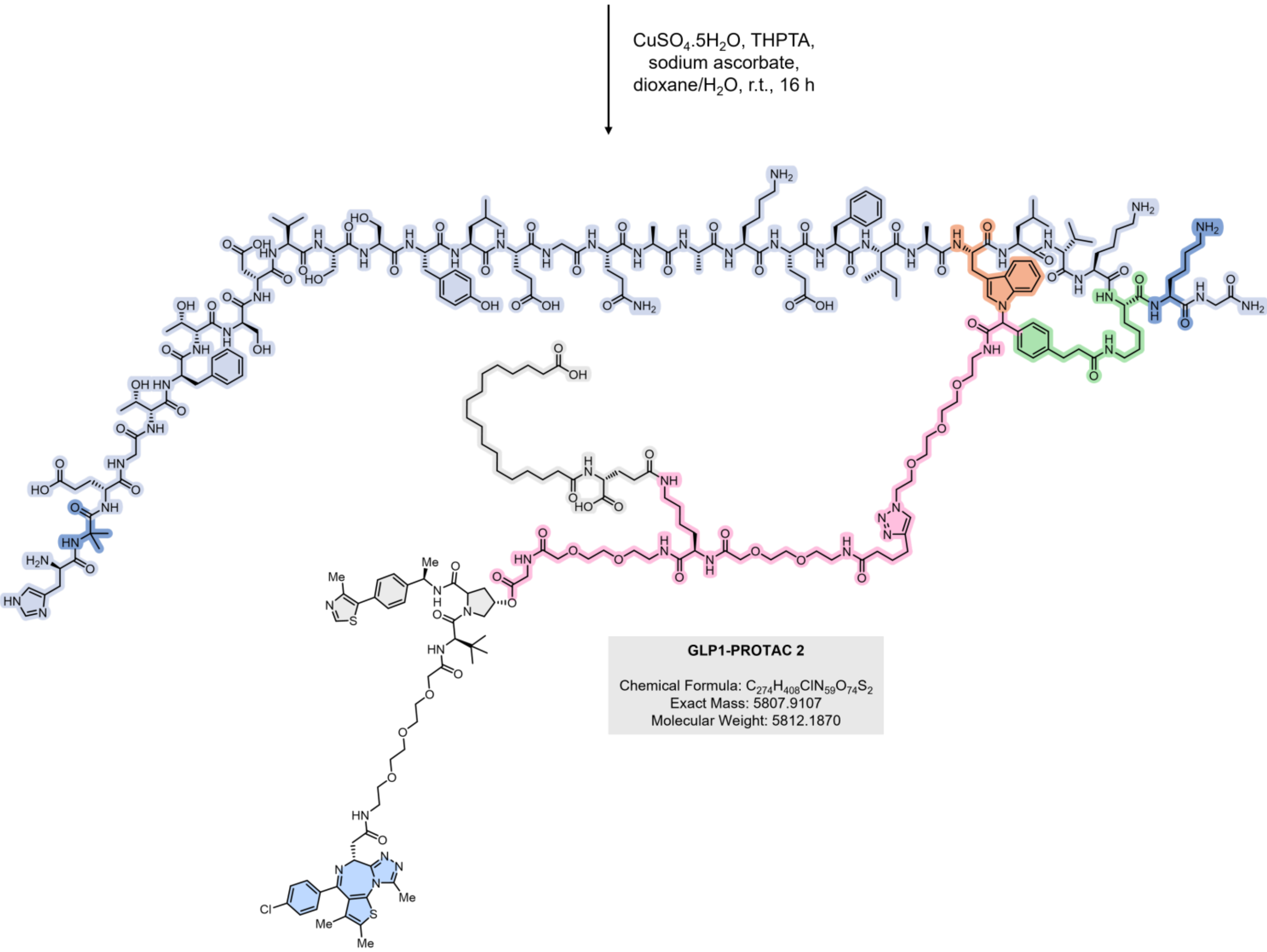

To a stirred solution of **12** (1 equiv, 9 mg, 0.0024 mmol) in dioxane/H_2O (1:1, 2 mL, v/v) was added **14** (1 equiv, 5 mg, 0.0024 mmol), $CuSO_4.5H_2O$ (8 equiv, 5 mg, 0.019 mmol), THPTA (4 equiv, 4 mg, 0.0096 mmol) and sodium ascorbate (10 equiv, 5 mg, 0.024 mmol). The reaction mixture was stirred at ambient temperature for 16 h, after which completion was confirmed by LC–MS analysis. The crude product was evaporated to dryness, dissolved in MeCN/H_2O (1:1) and purified by preparative HPLC (50–100% B), affording compound **GLP1-PROTAC 2** as an off-white solid (1.23 mg, 0.00021 mmol, 9% yield).

UV LCMS R_t = 2.32 min (5–95% B over 5 min)
Purity: >95%
LRMS calcd. for $C_{274}H_{410}ClN_{59}O_{74}S_2^{2+}$ *m/z* 5809.9252 [M+2H]$^{2+}$, found 1163.55 [M/5+2H]$^{2+}$

2. Analytical and UV LCMS spectra

- ***Analytical HPLC (at 210 nm) and MS traces of peptide hGLP-1***

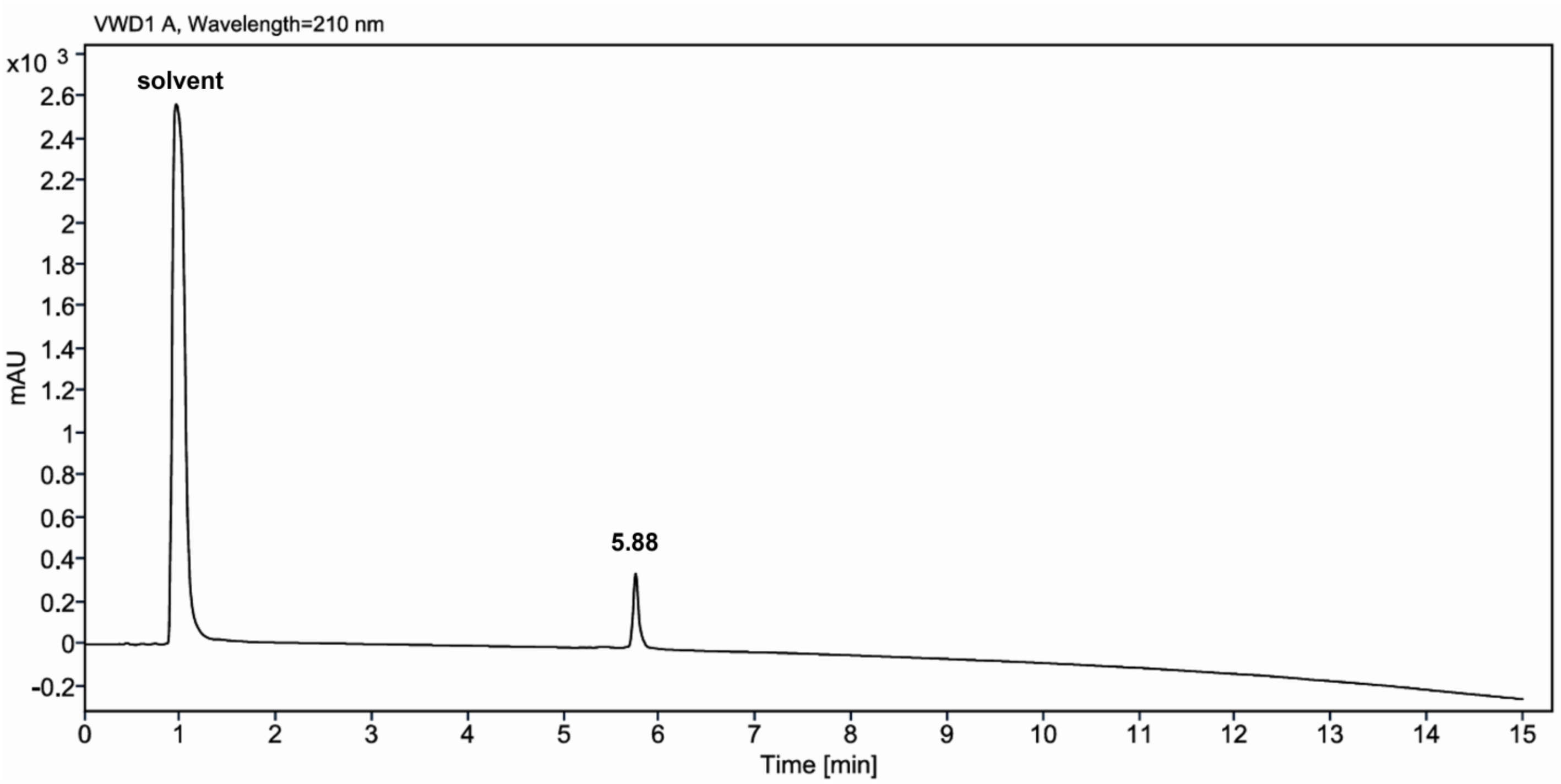

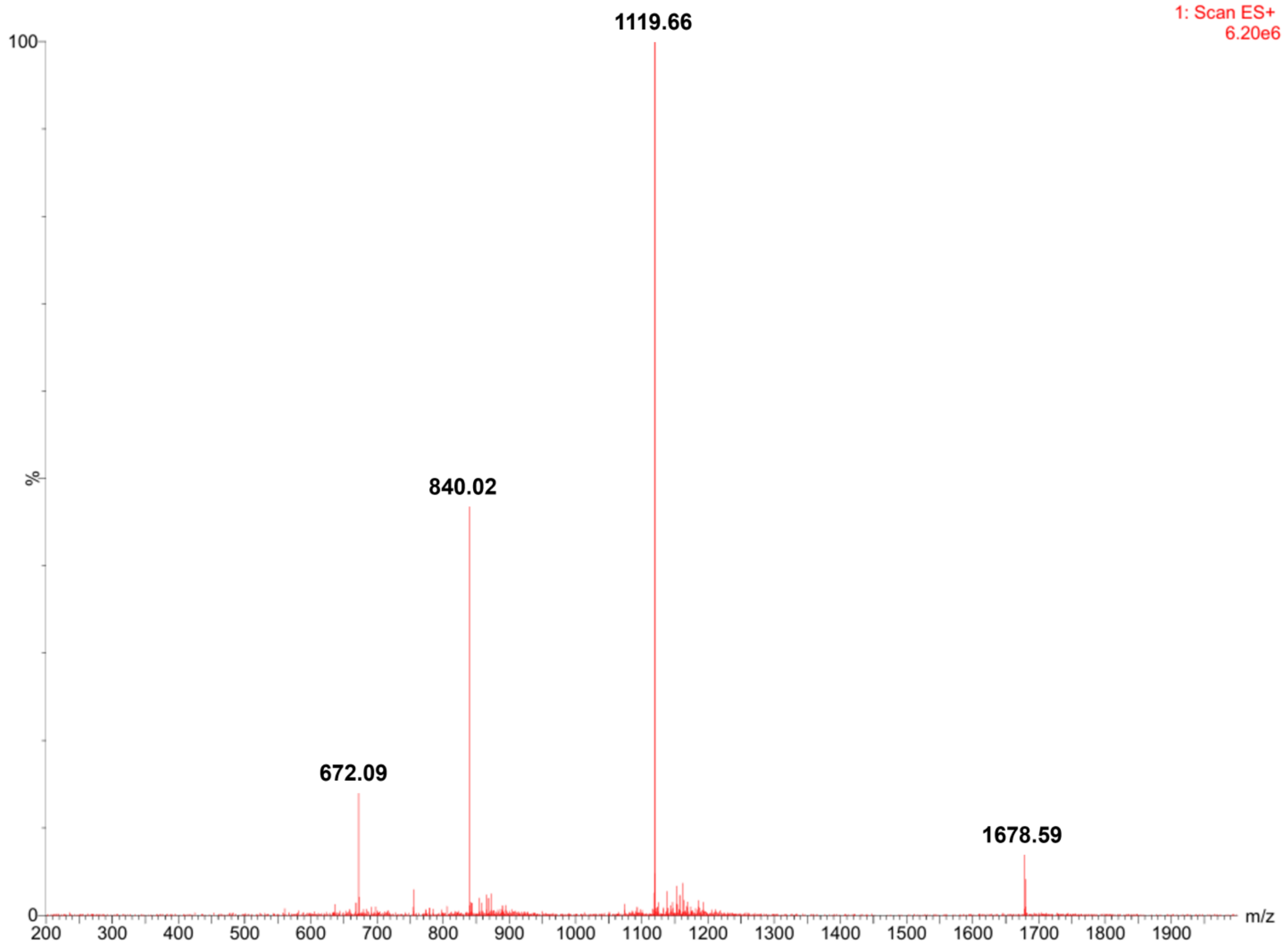

- ***Analytical HPLC (at 210 nm) and MS traces of peptide hGLP-1 R36K***

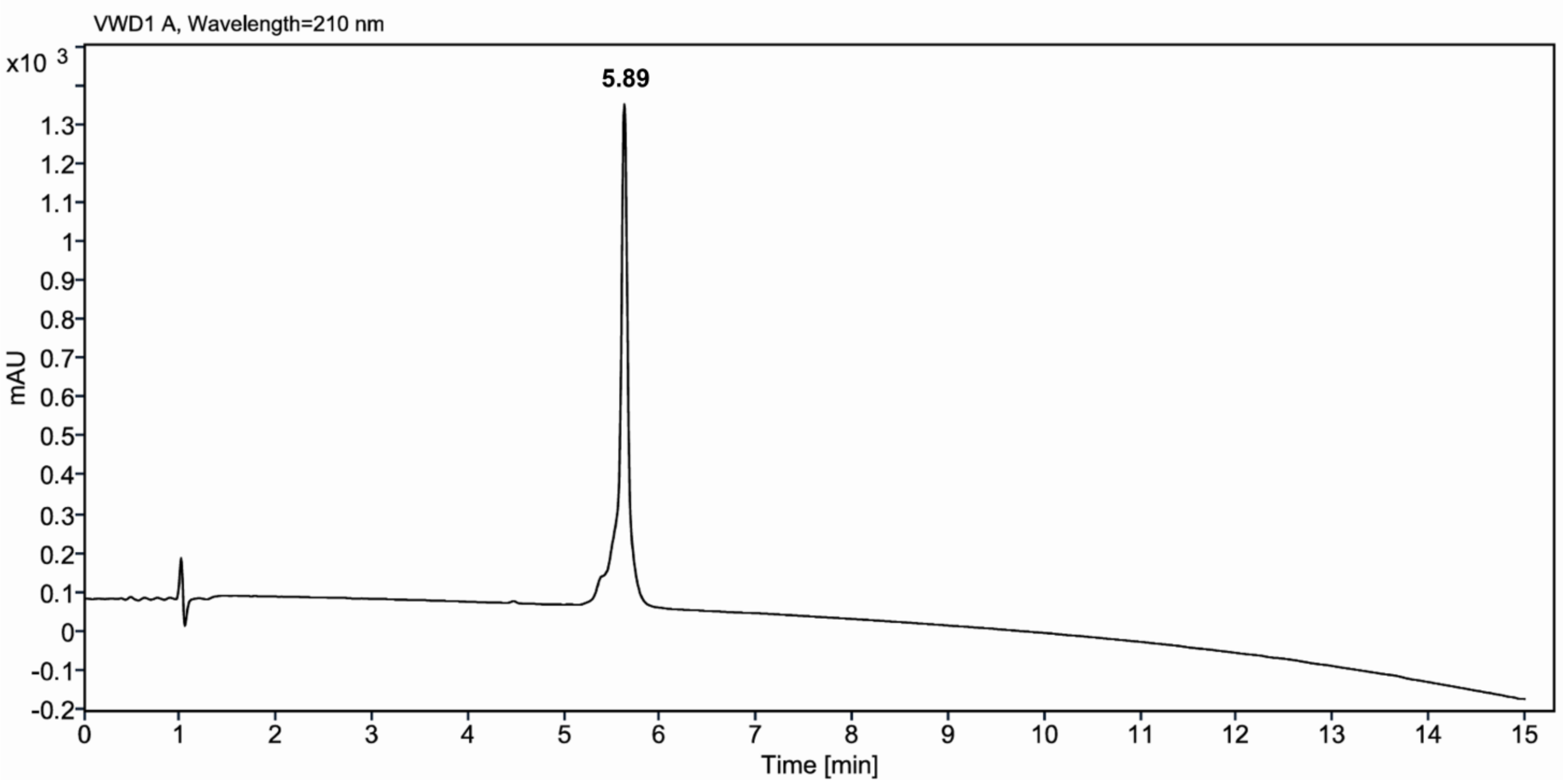

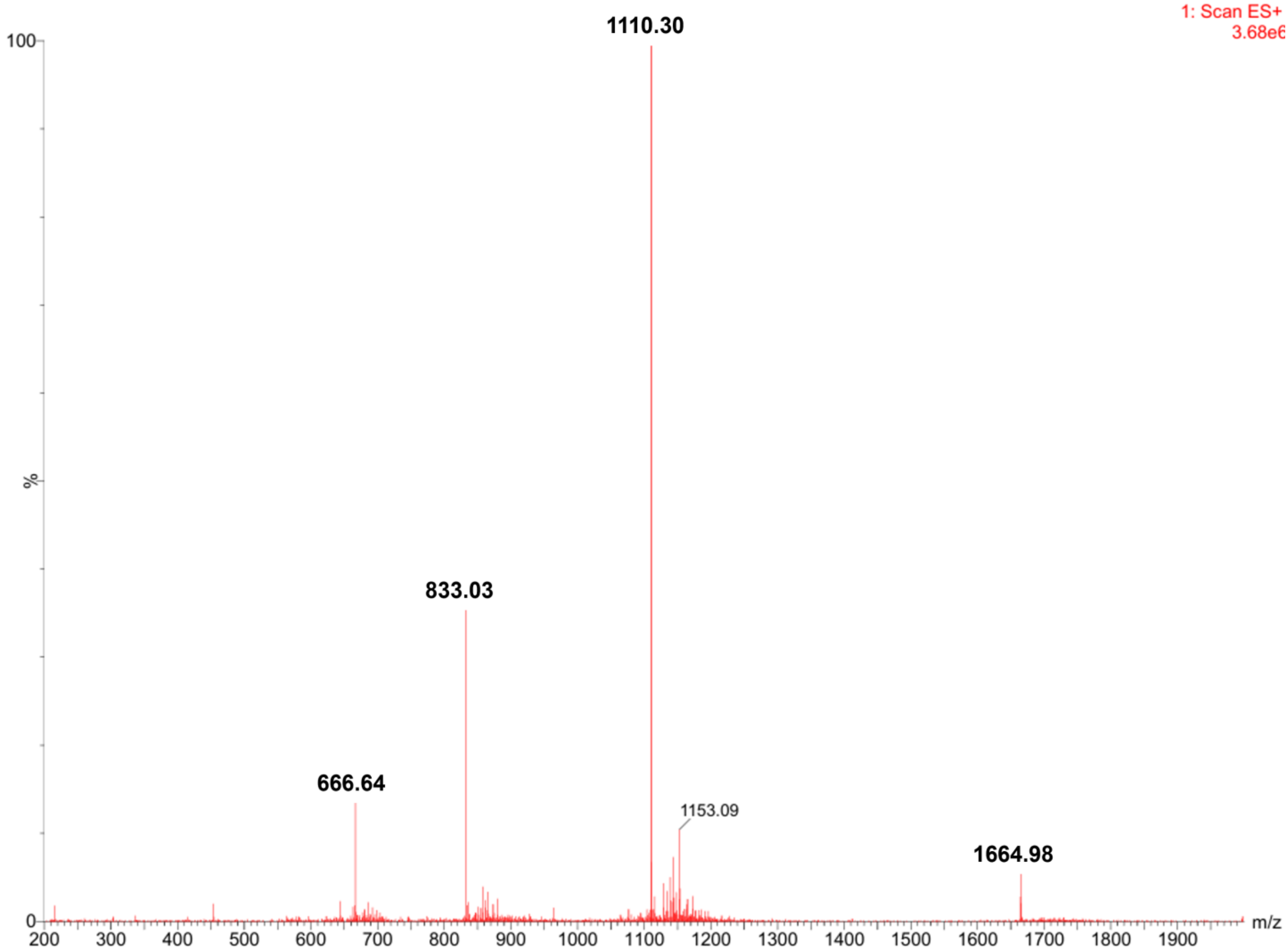

- ***Analytical HPLC (at 210 nm) and MS traces of peptide hGLP-1 A8Aib***

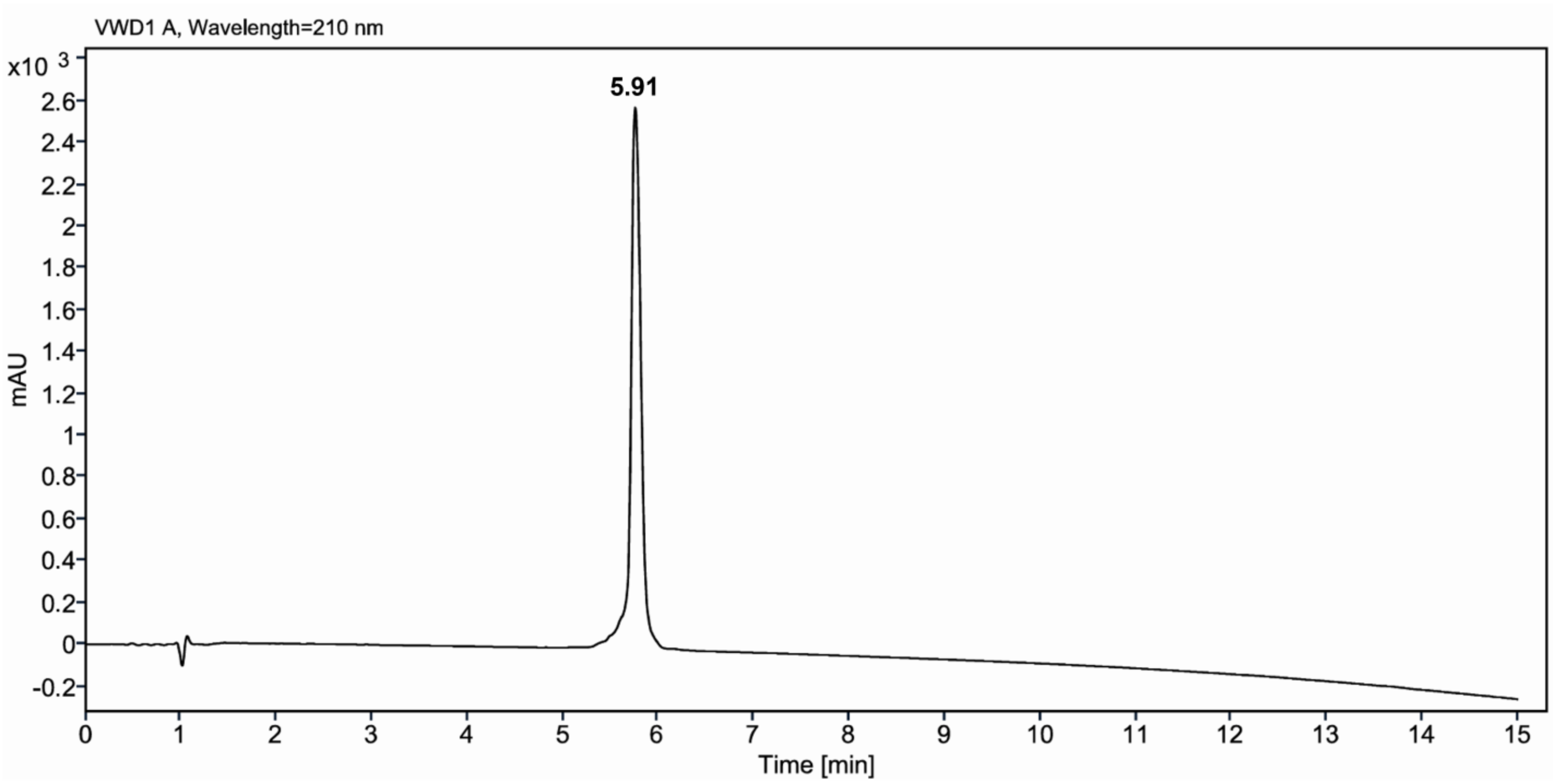

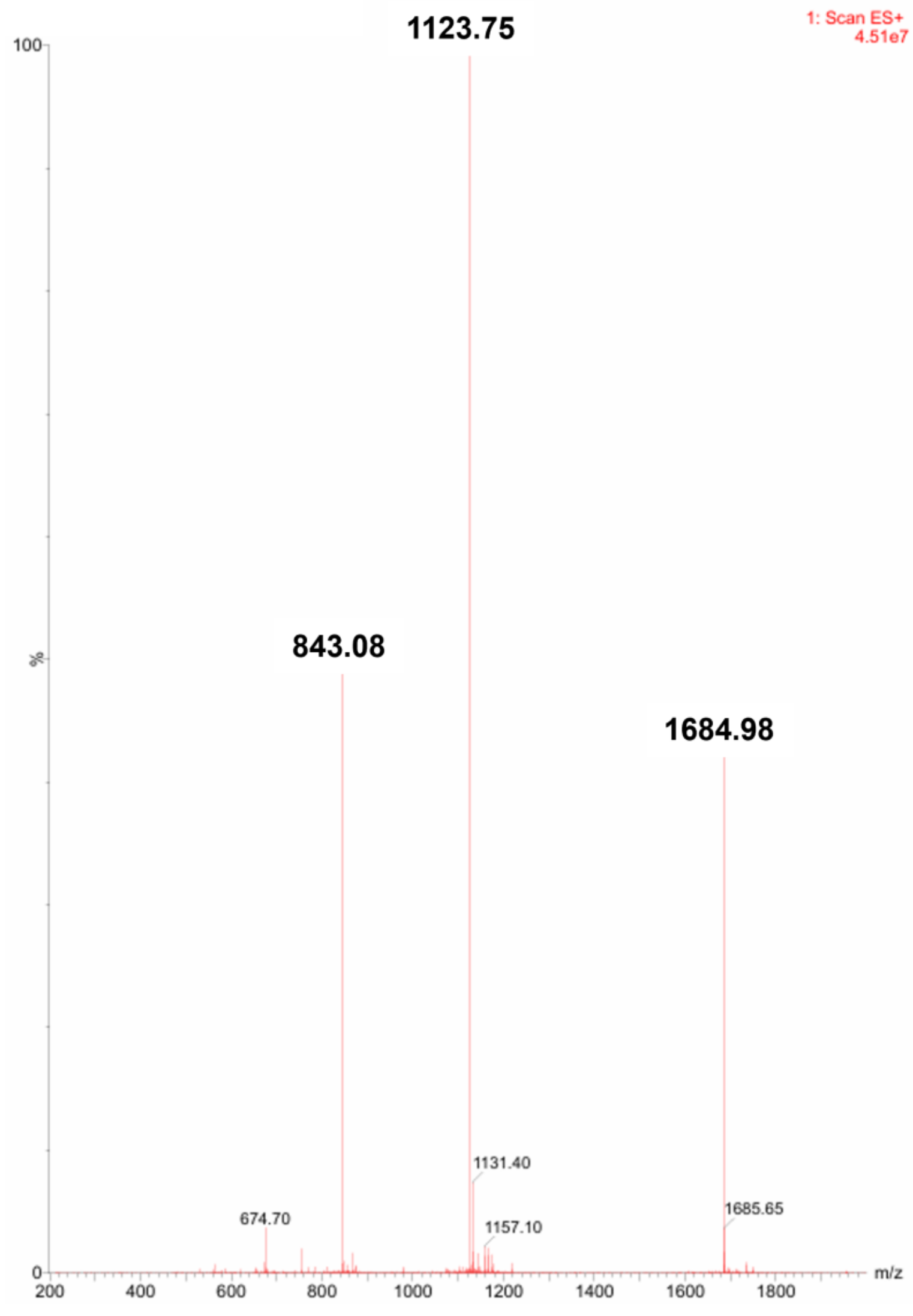

- ***Analytical HPLC (at 220 nm) and MS traces of peptide 1***

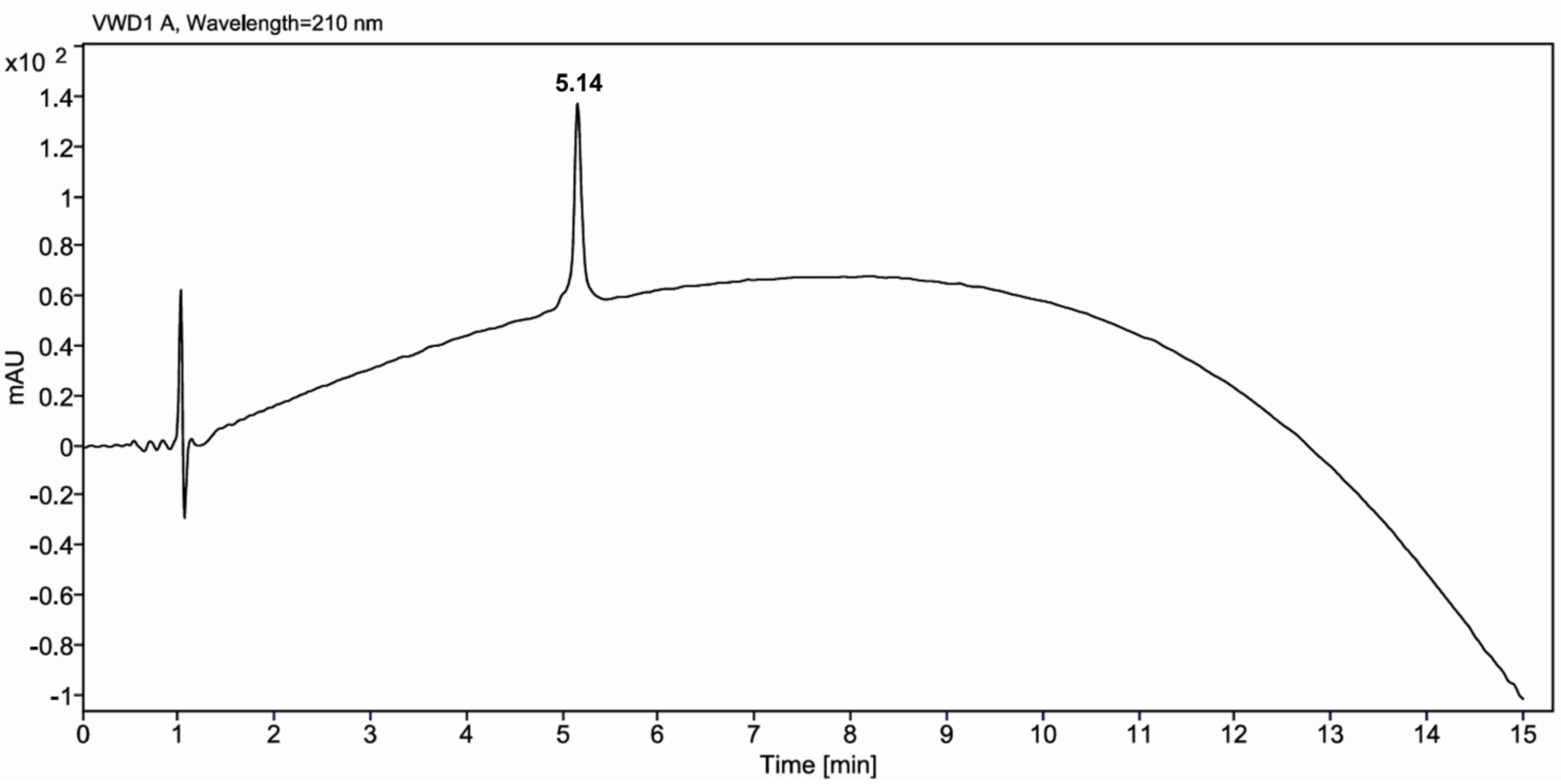

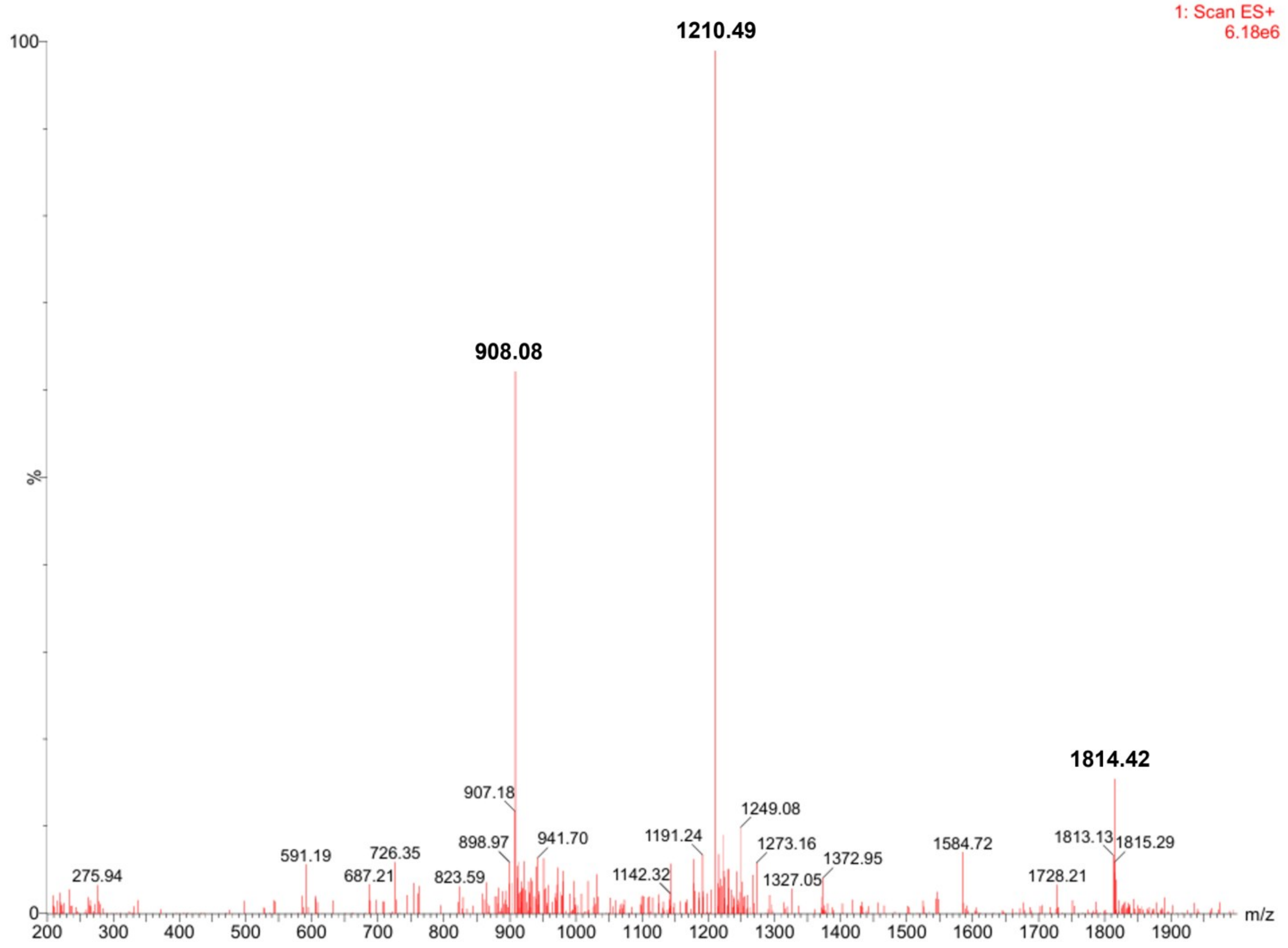

- ***Analytical HPLC (at 220 nm) and MS traces of peptide 2***

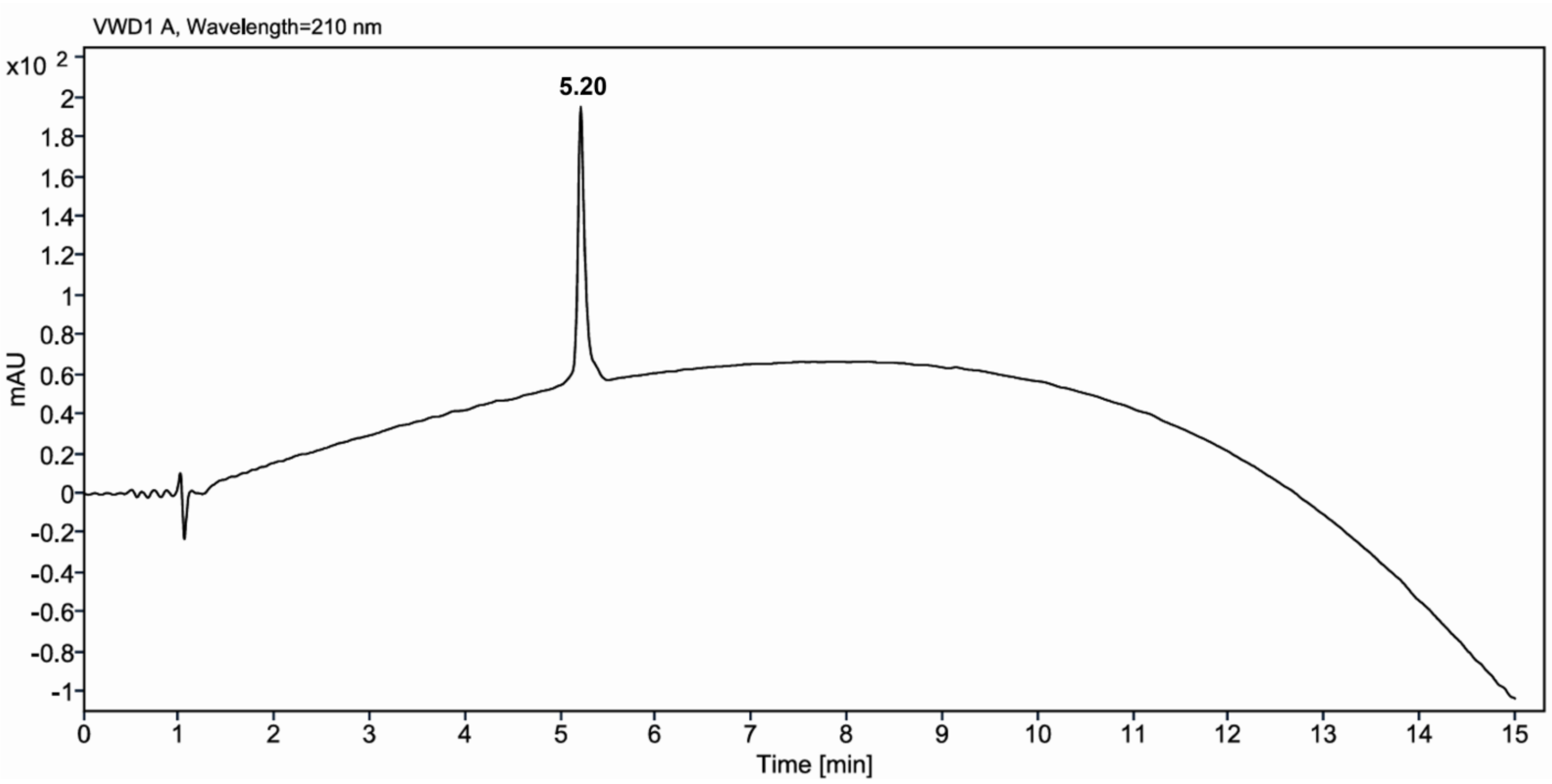

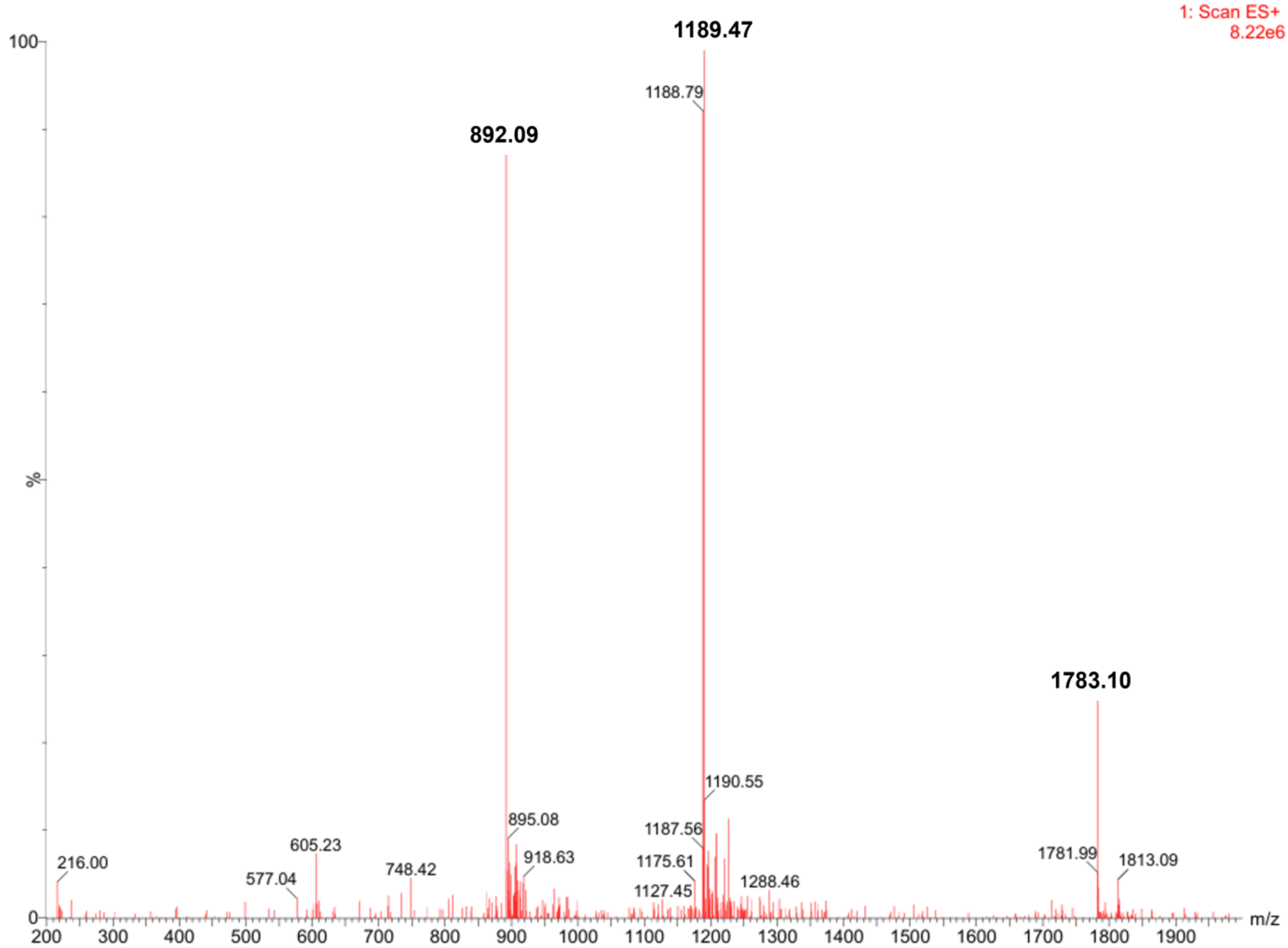

- ***Analytical HPLC (at 210 nm) and MS traces of peptide 3***

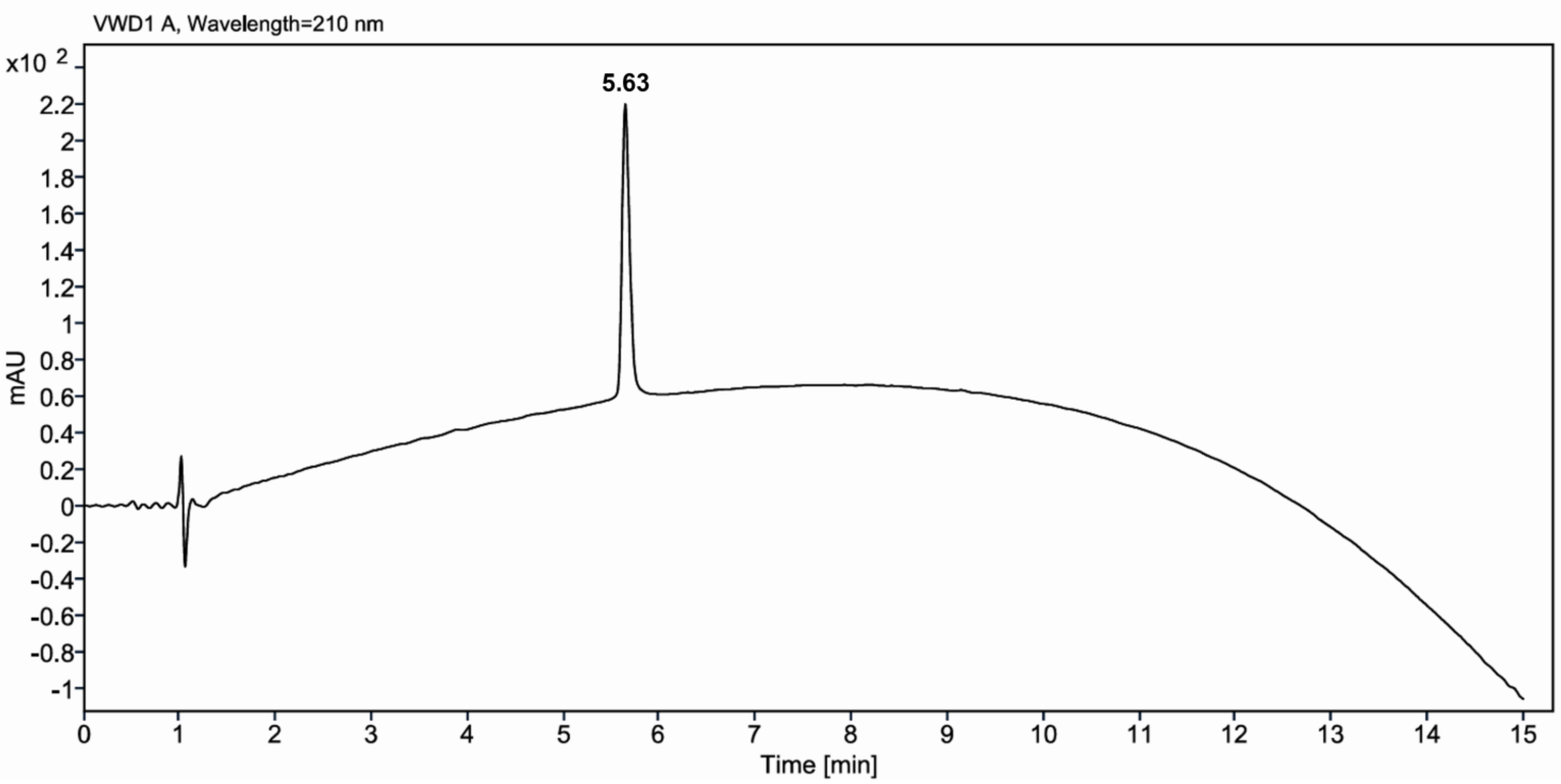

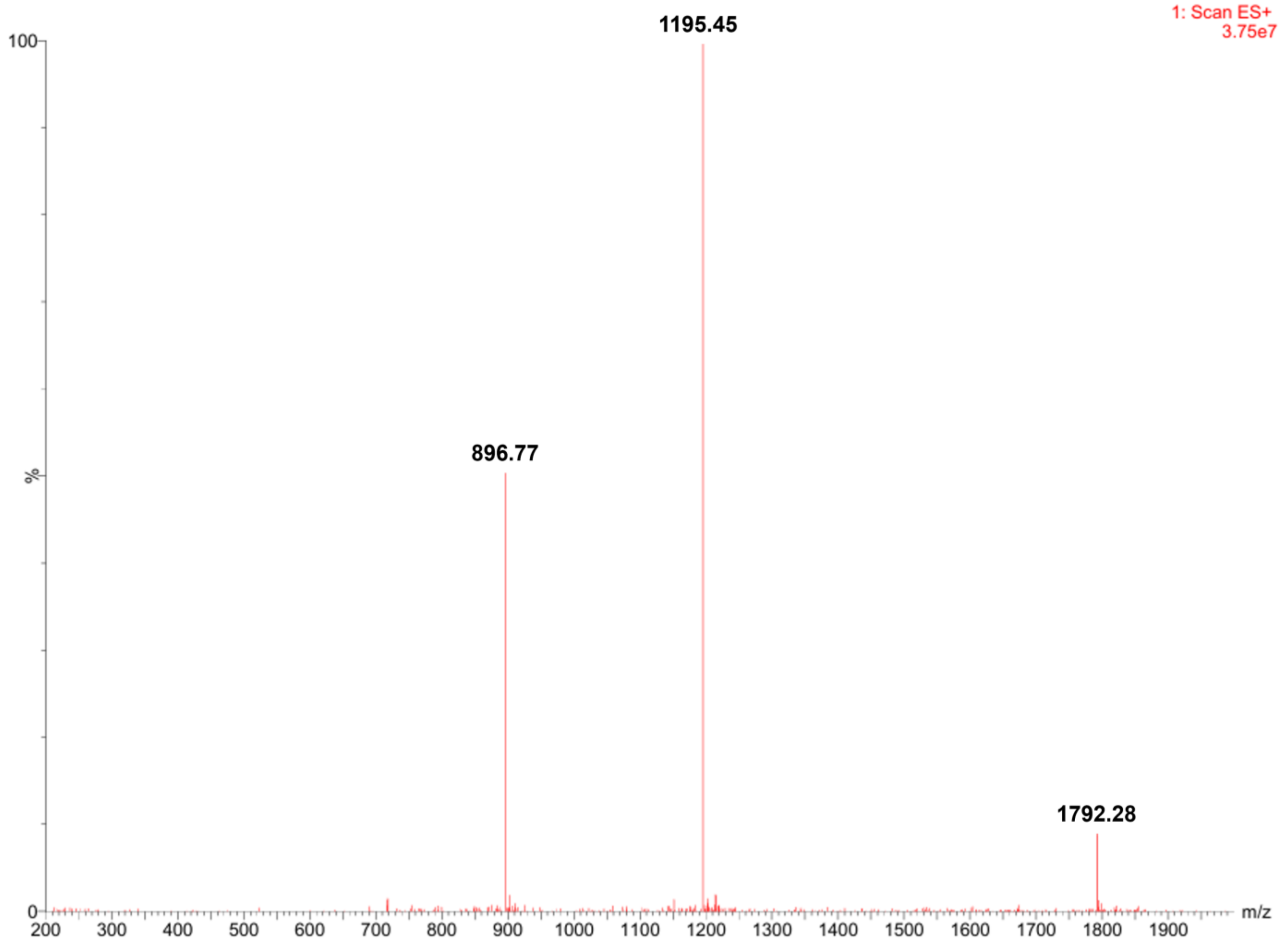

- ***Analytical HPLC (at 210 nm) and MS traces of peptide 4***

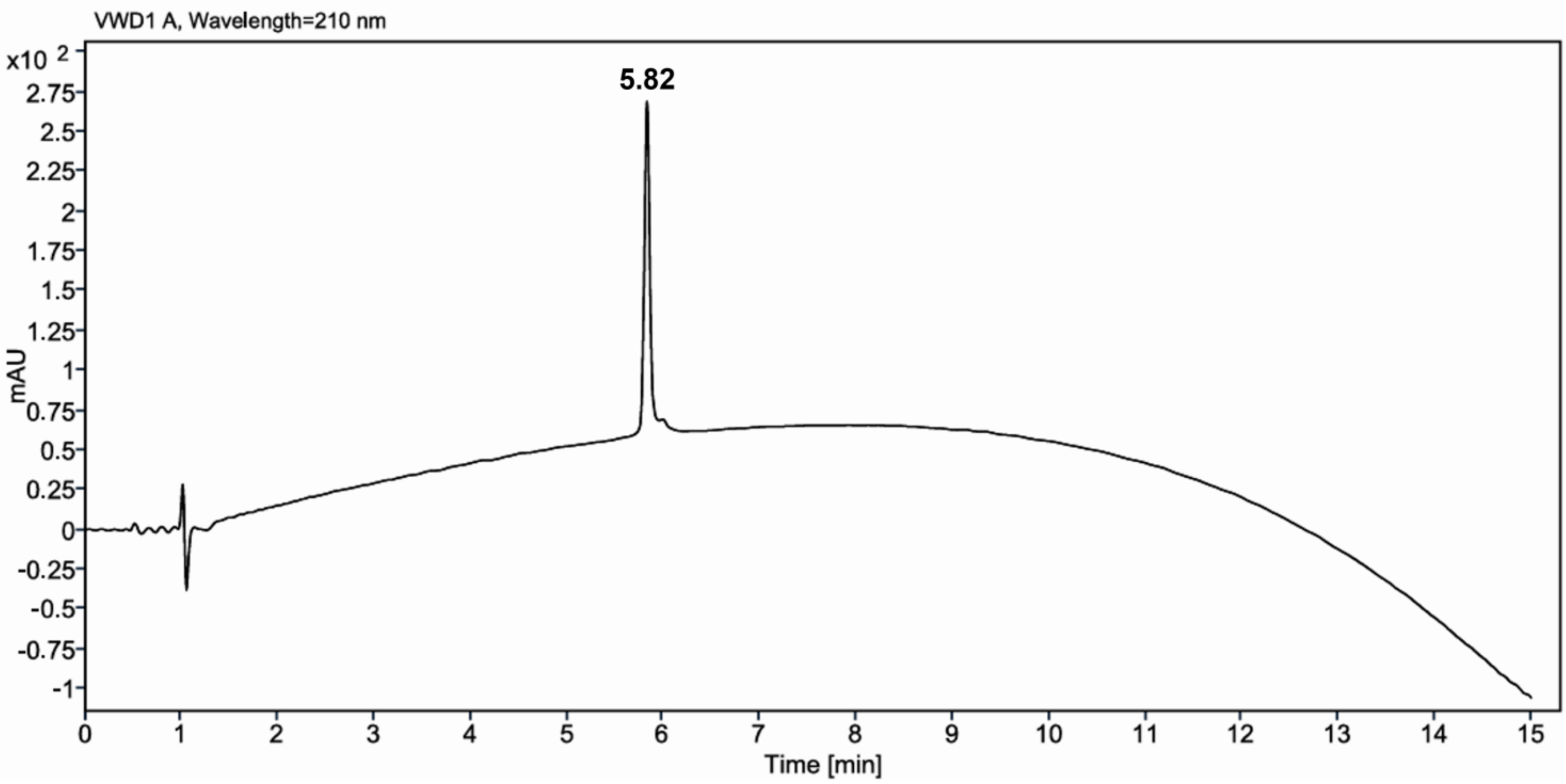

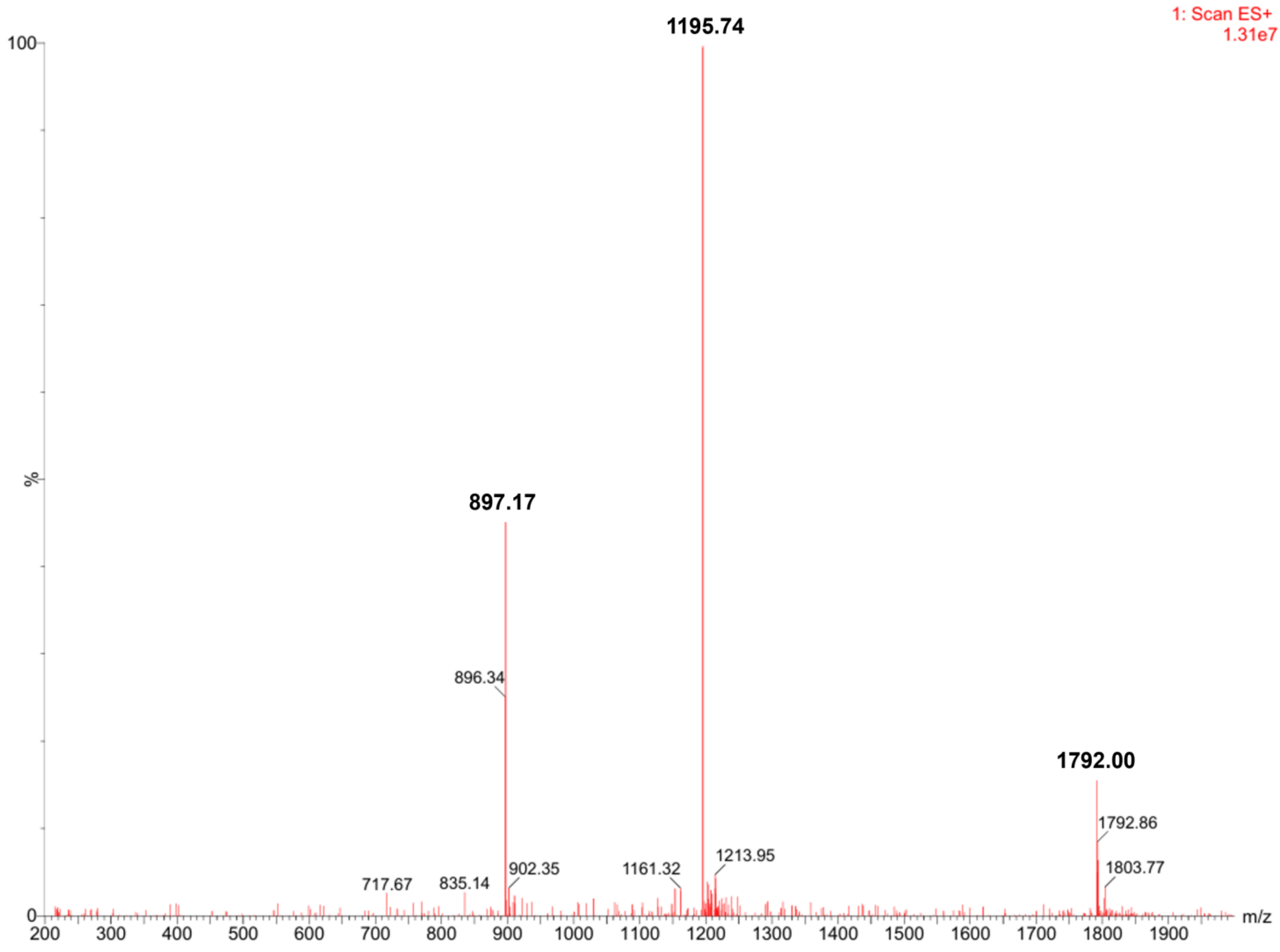

- ***Analytical HPLC (at 210 nm) and MS traces of peptide 5***

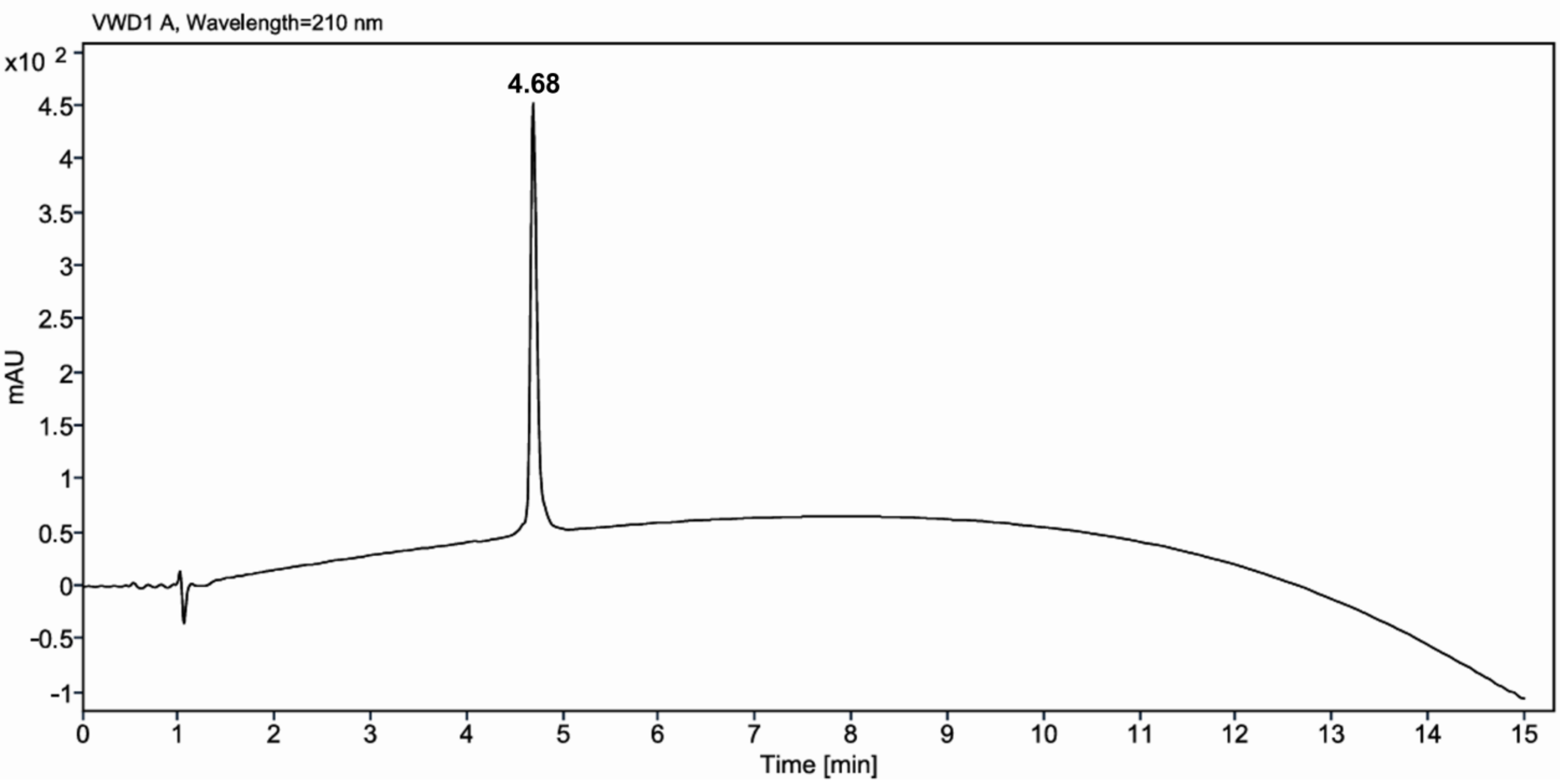

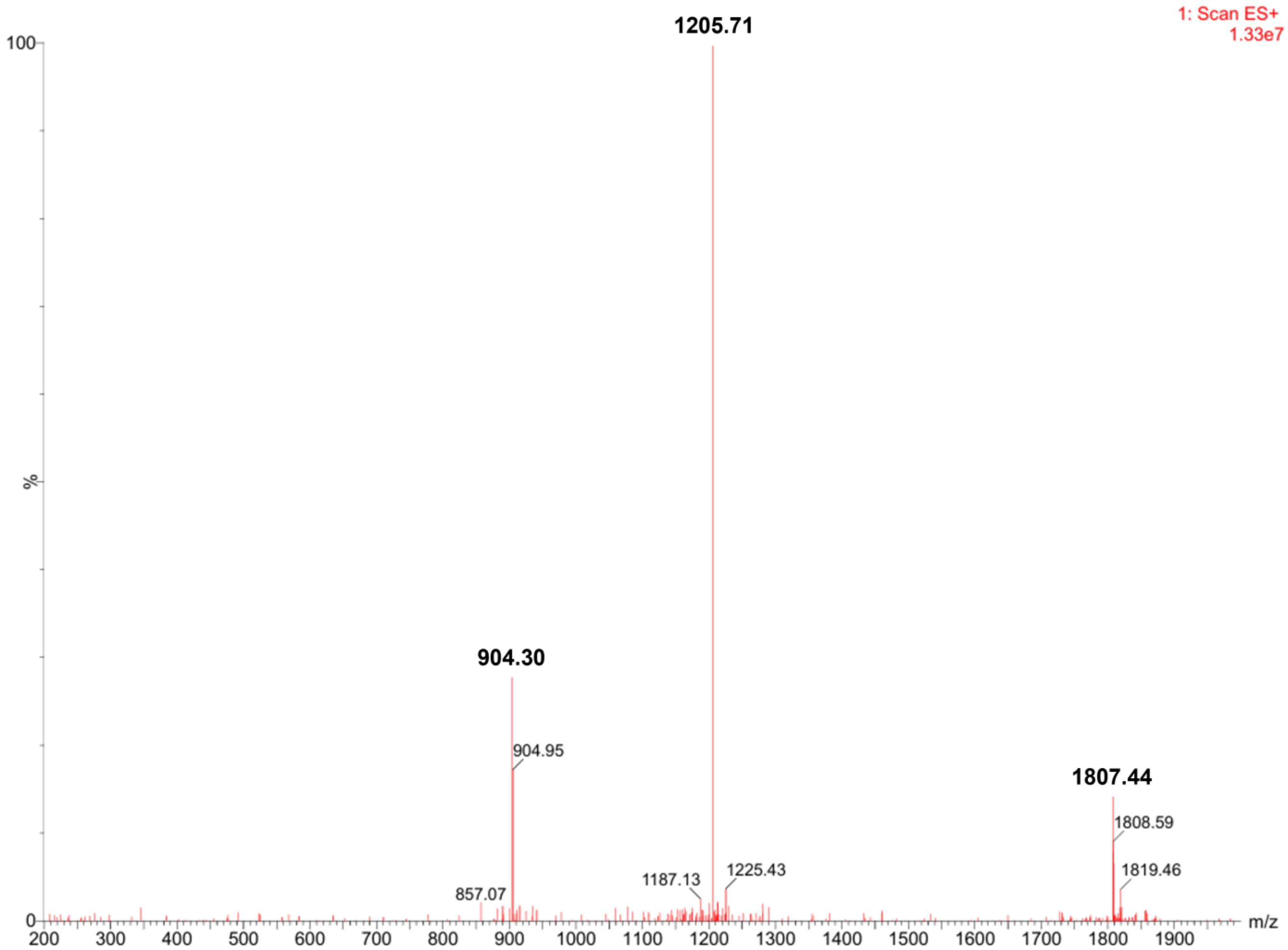

- ***Analytical HPLC (at 210 nm) and MS traces of peptide 6***

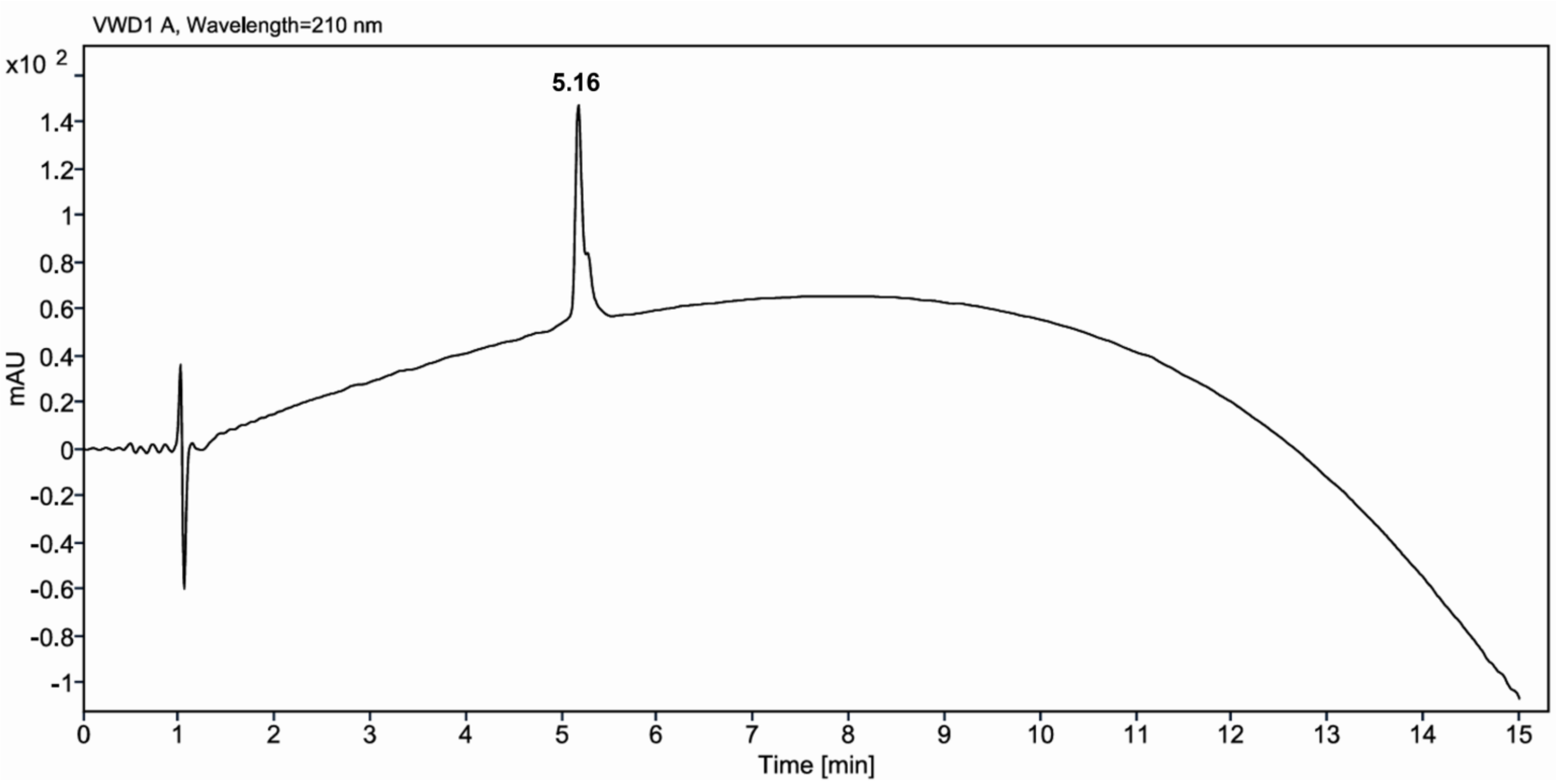

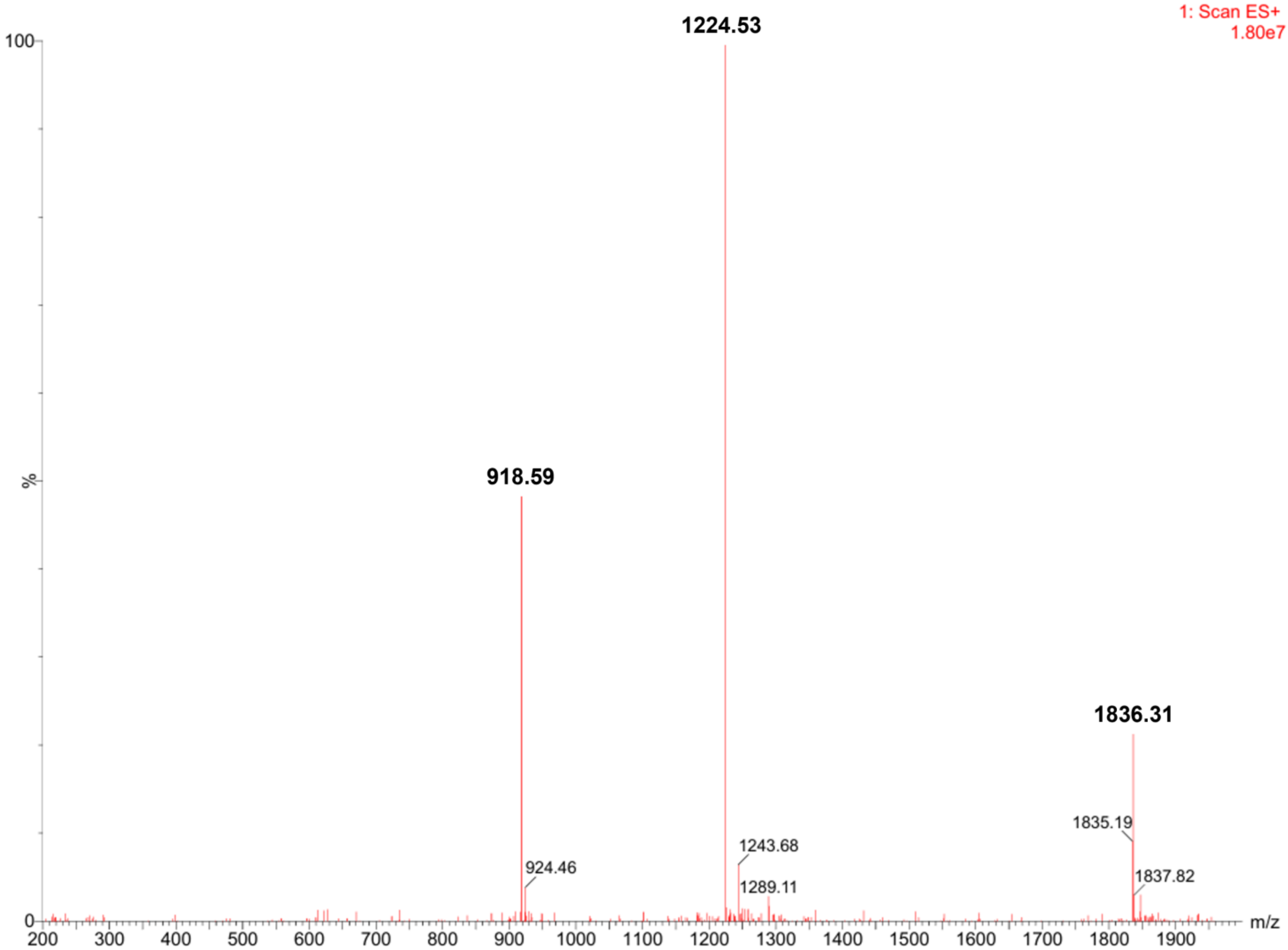

- ***Analytical HPLC (at 210 nm) and MS traces of peptide 7***

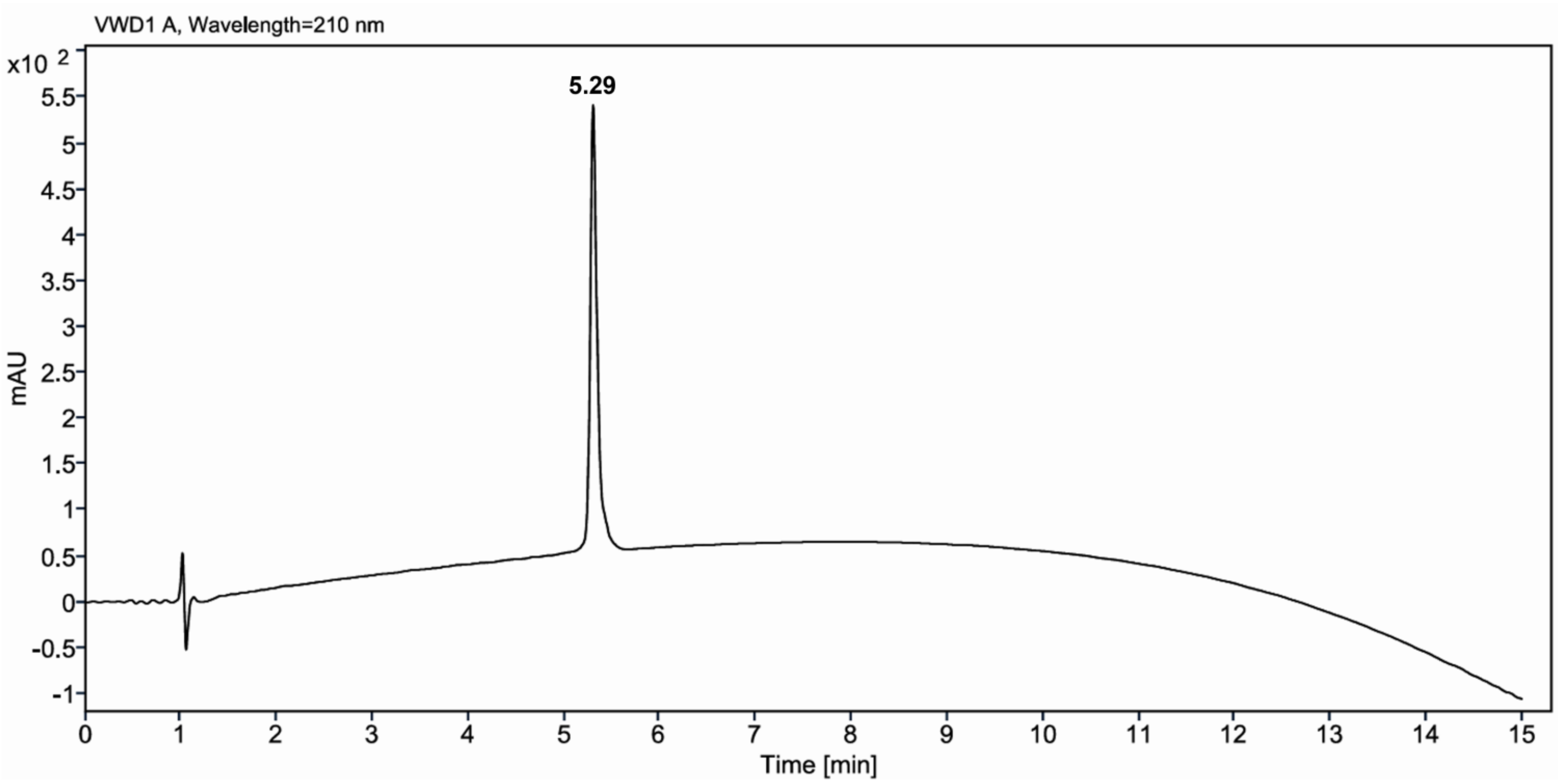

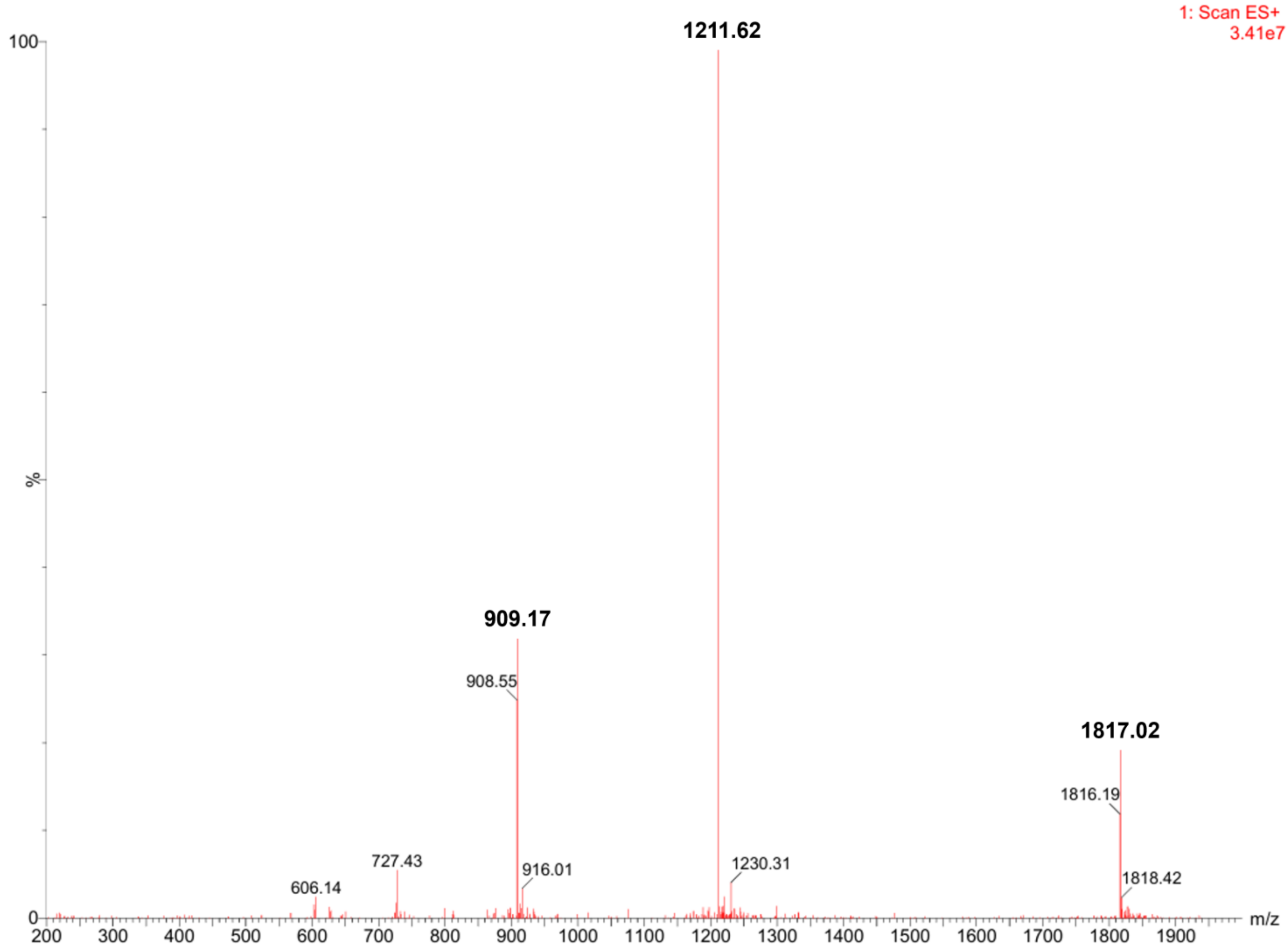

- ***Analytical HPLC (at 210 nm) and MS traces of peptide 8***

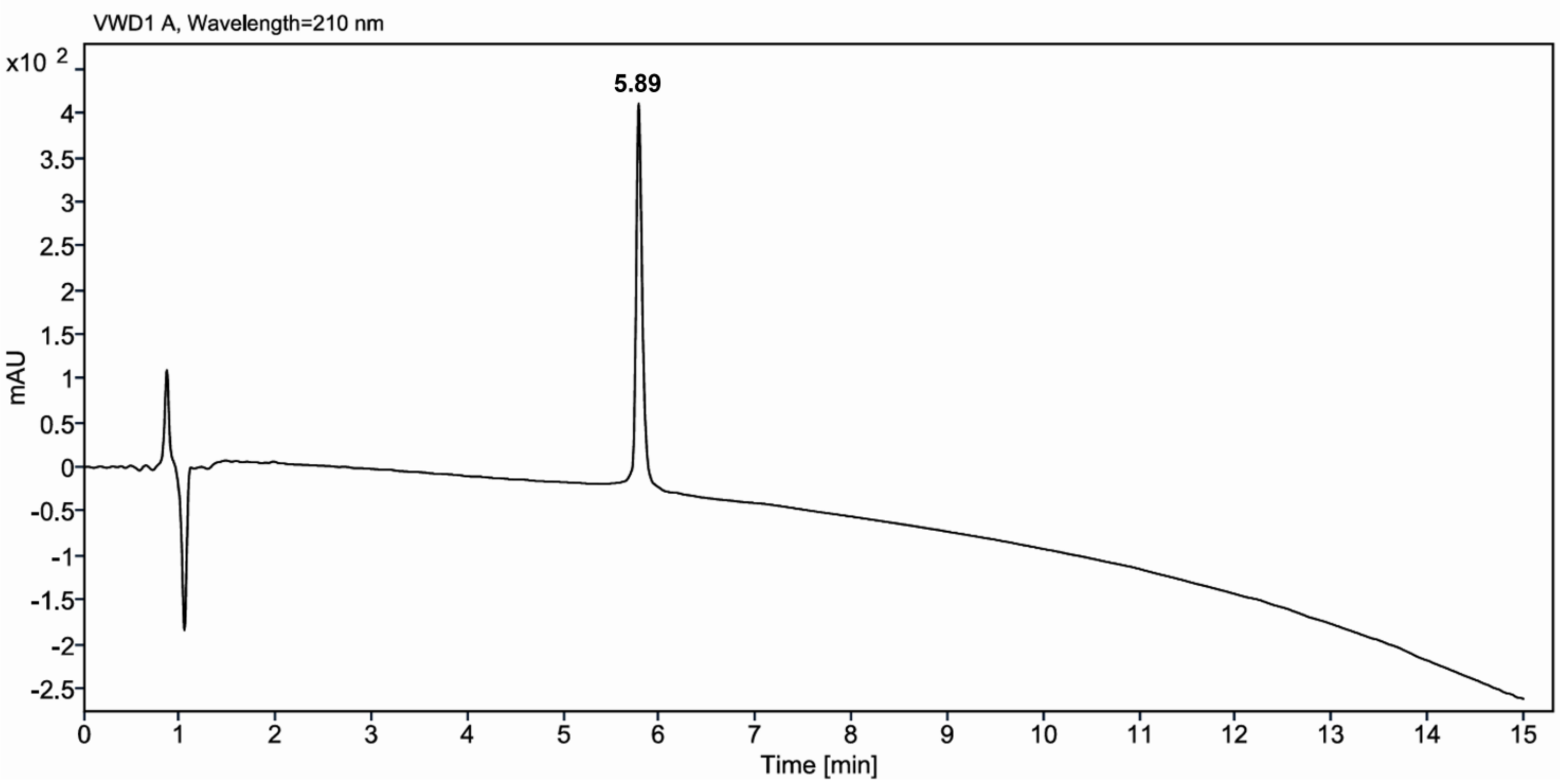

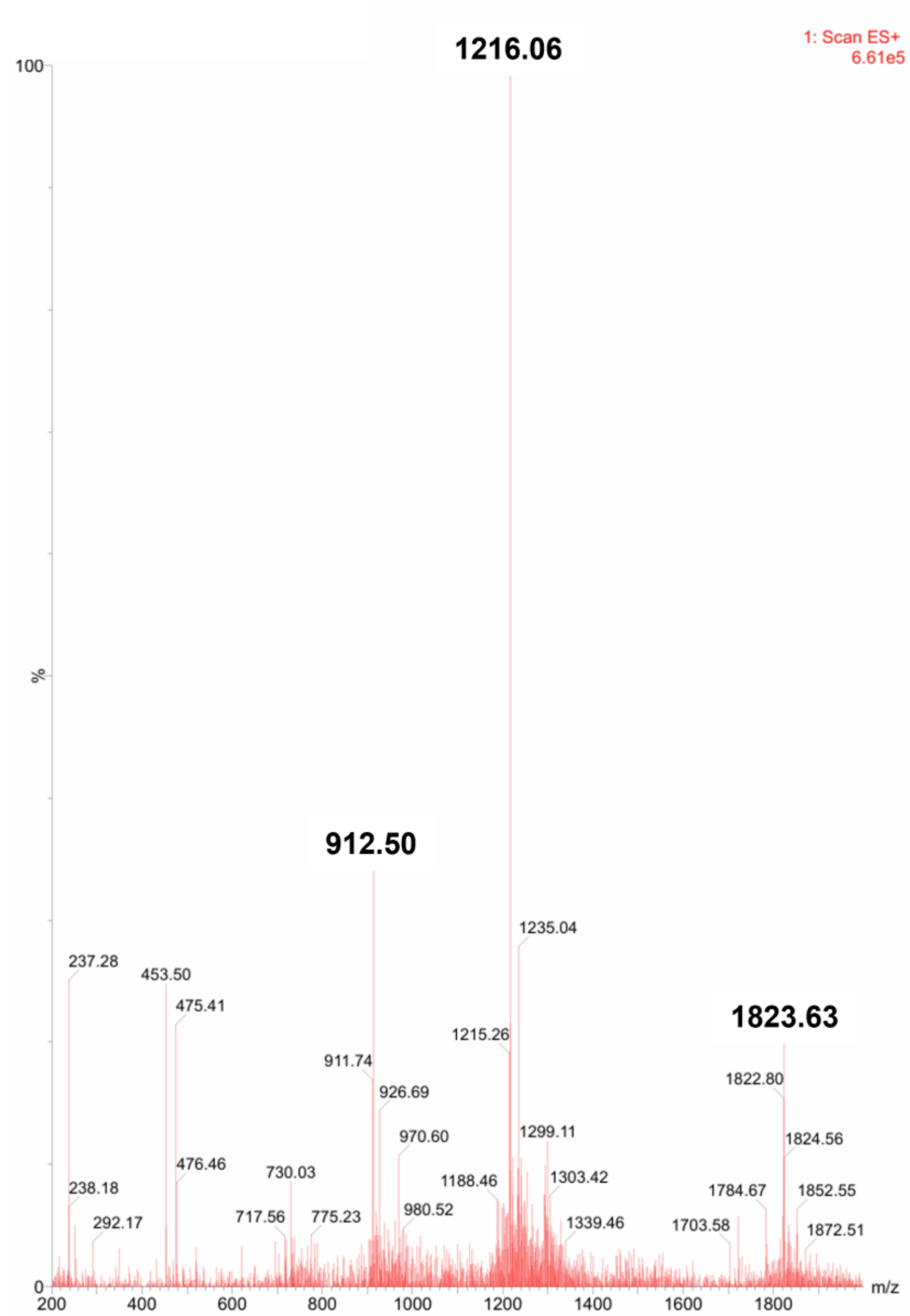

- ***Analytical HPLC (at 280 nm) and MS traces of 9***

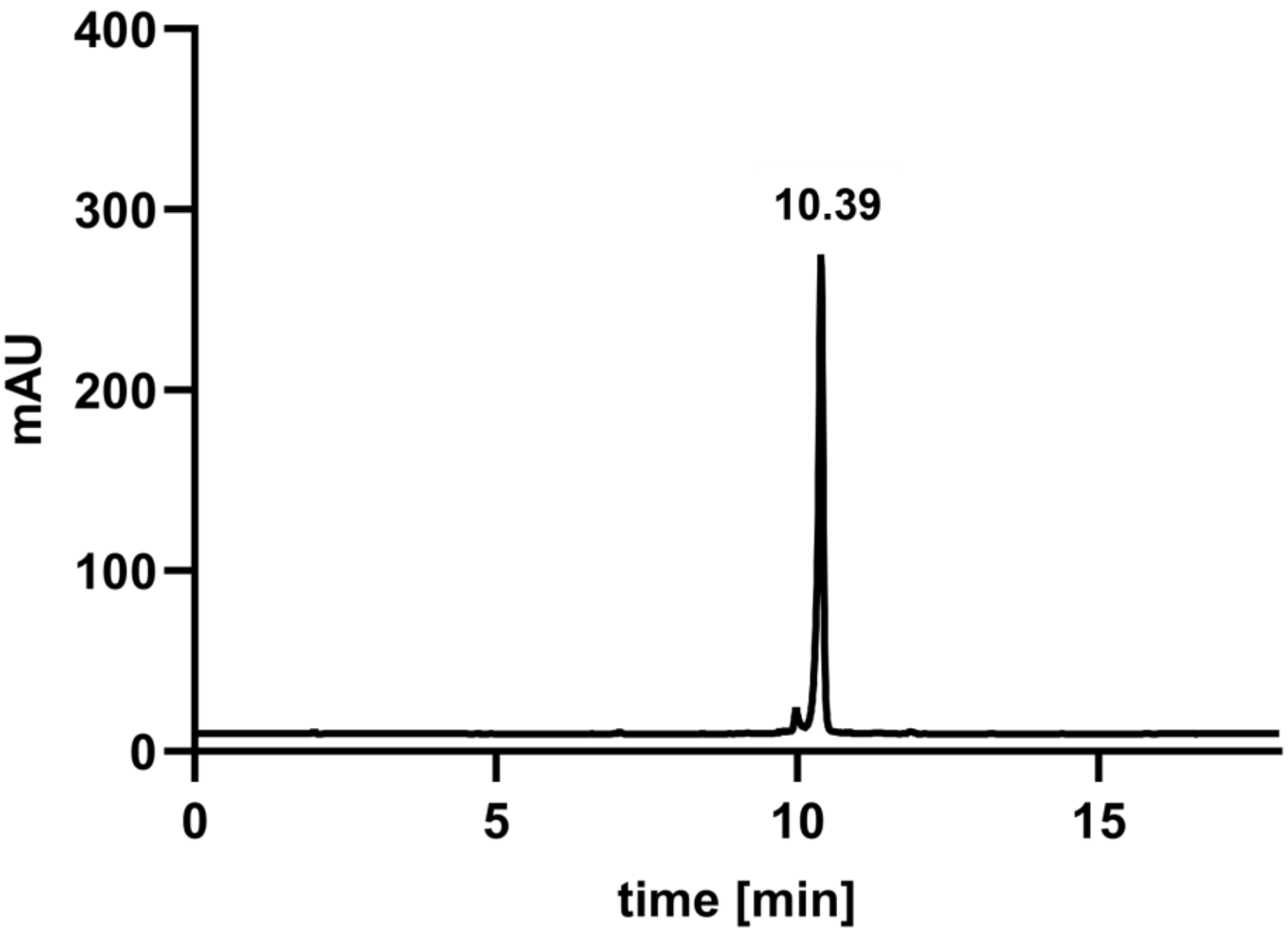

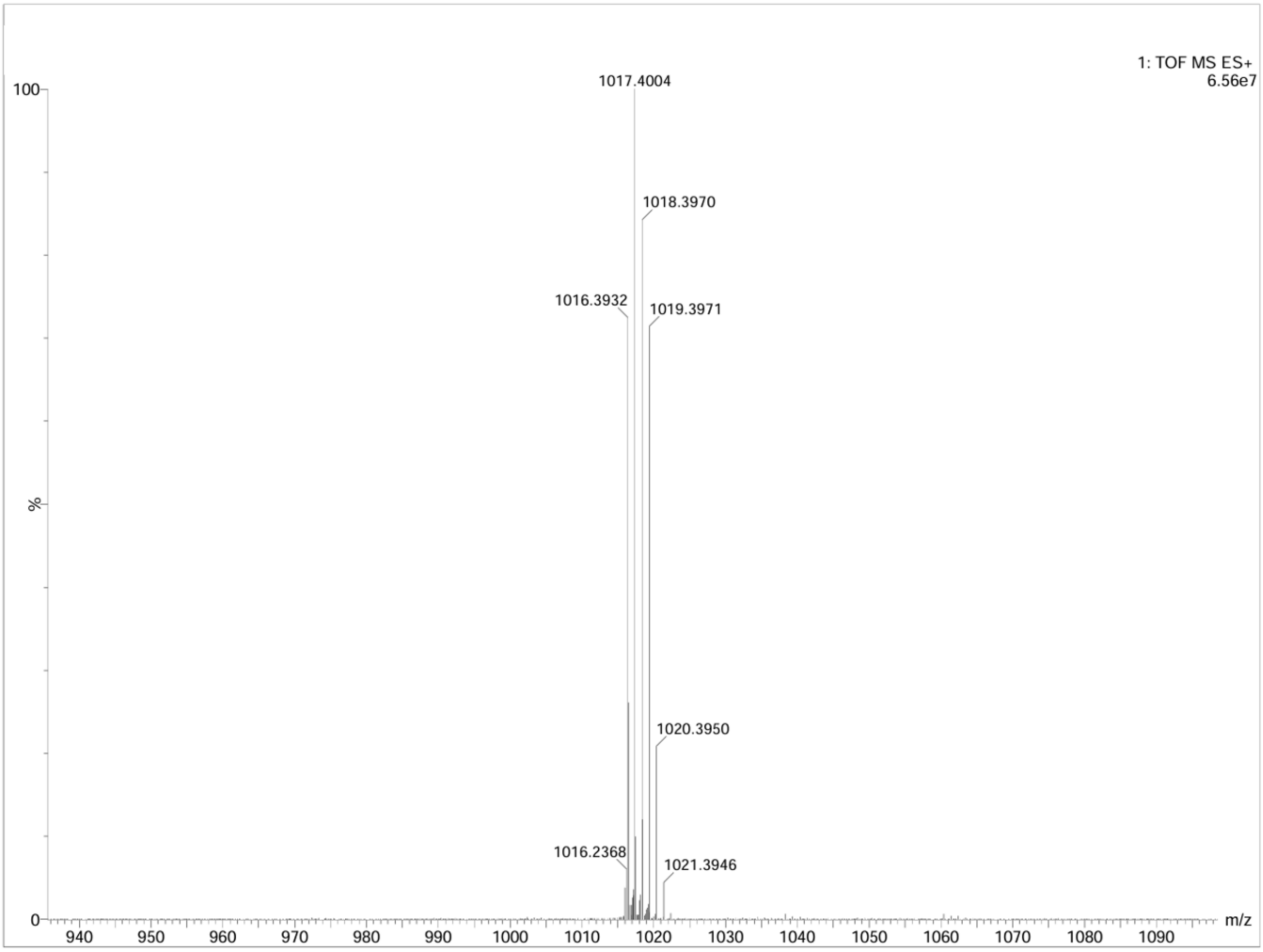

▪ ***Analytical HPLC (at 280 nm) and MS traces of 11***

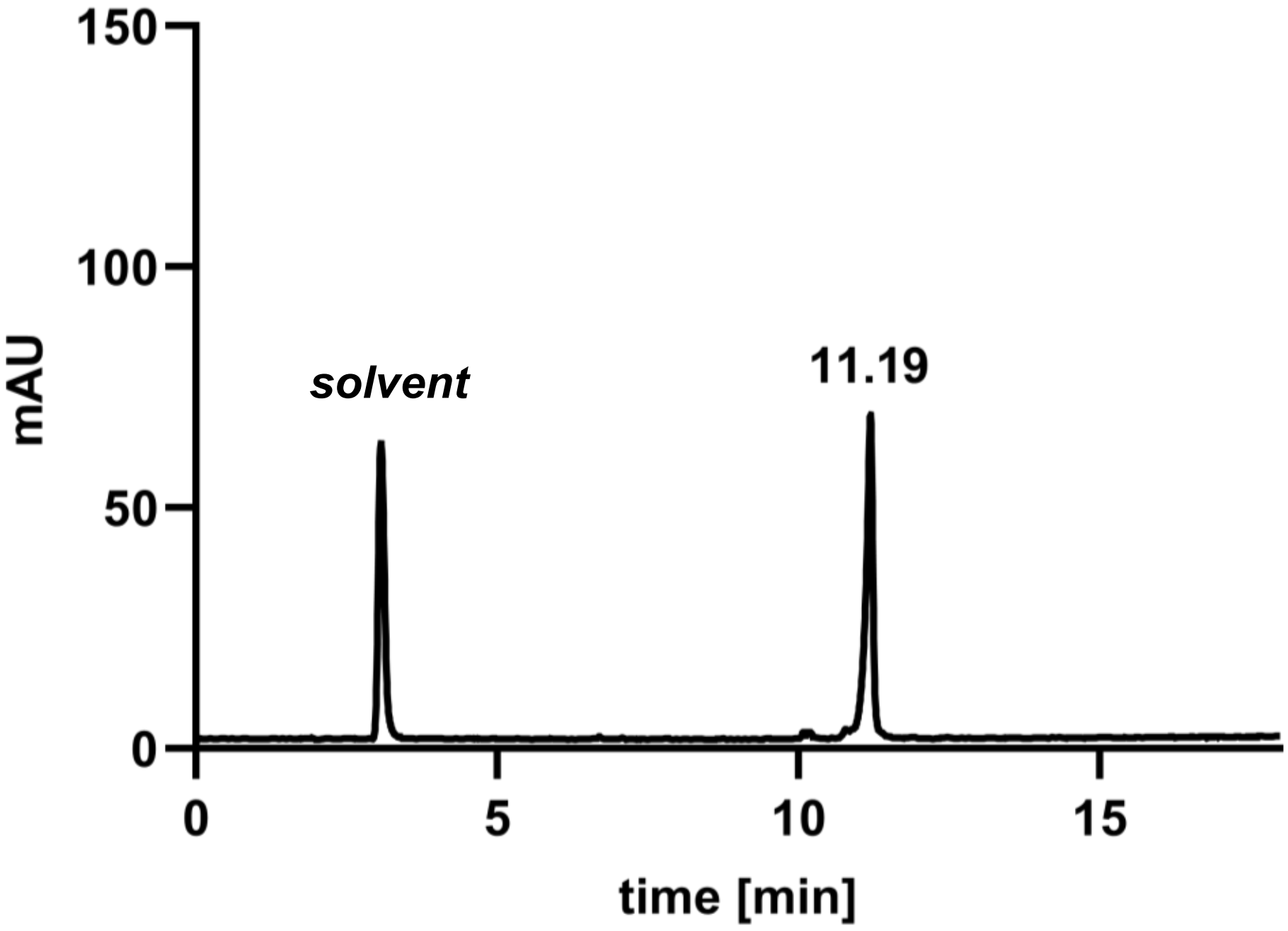

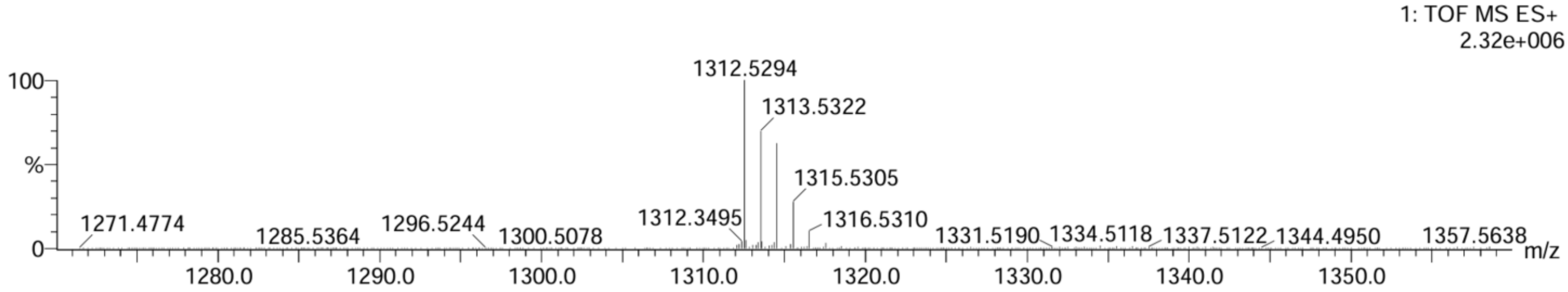

- ***Analytical HPLC (at 254 nm) and MS traces of 14***

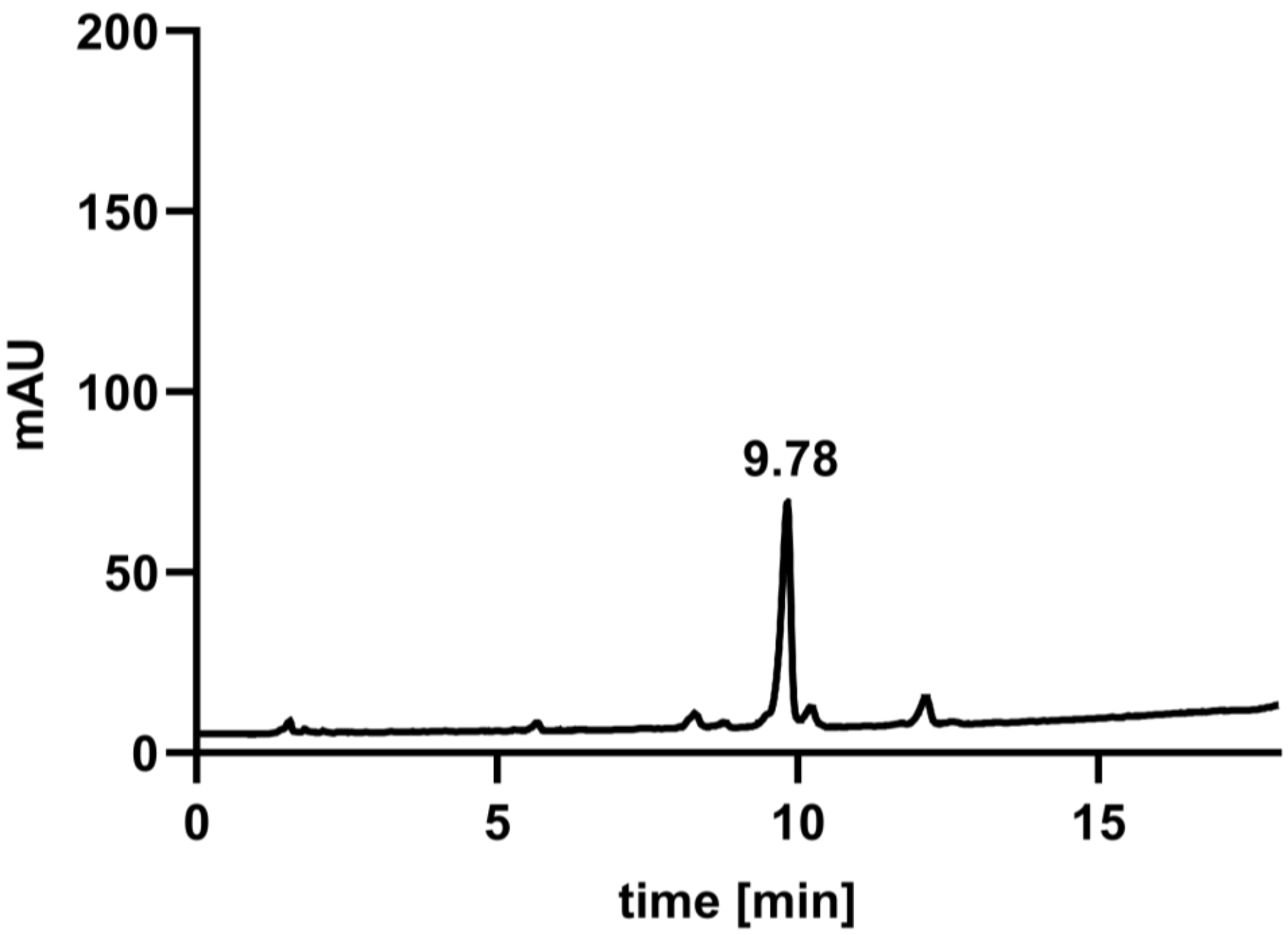

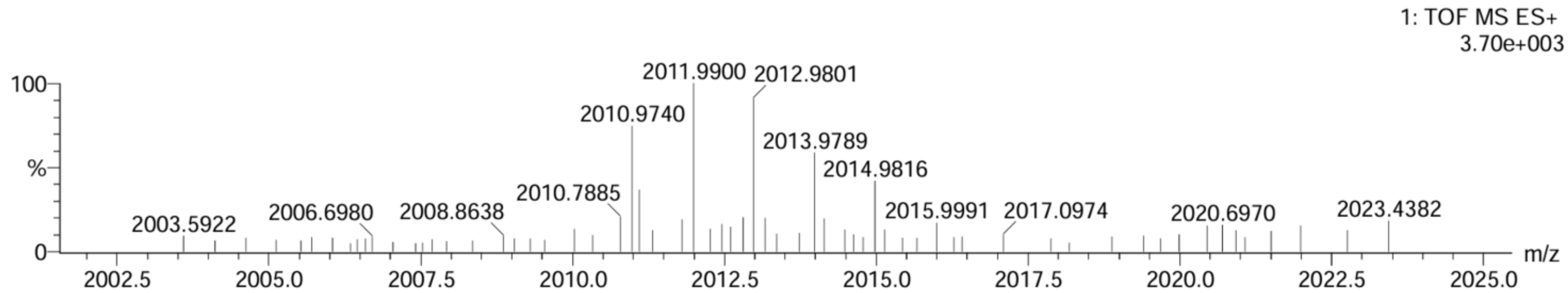

- ***Analytical HPLC (at 280 nm) and MS traces of 12***

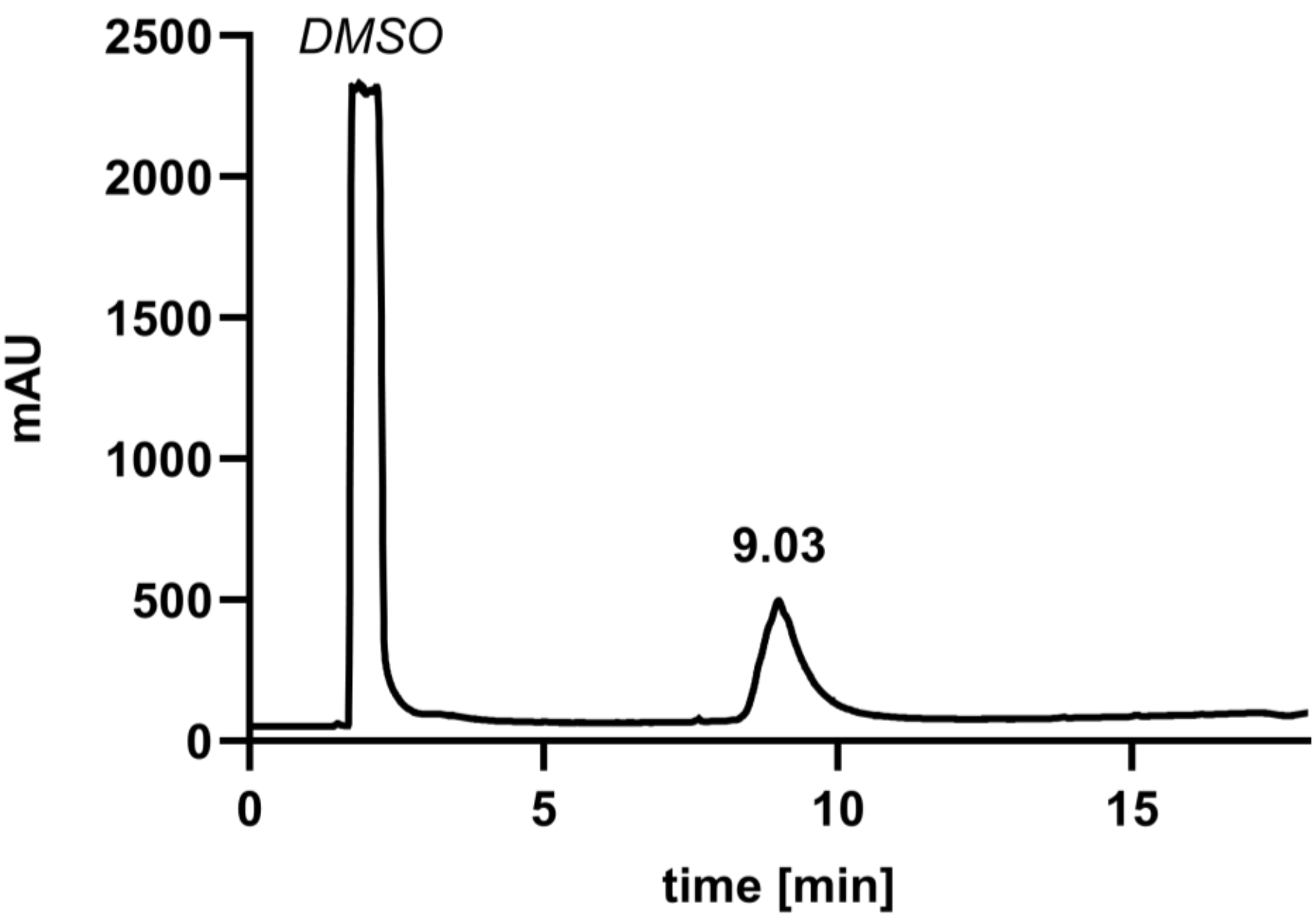

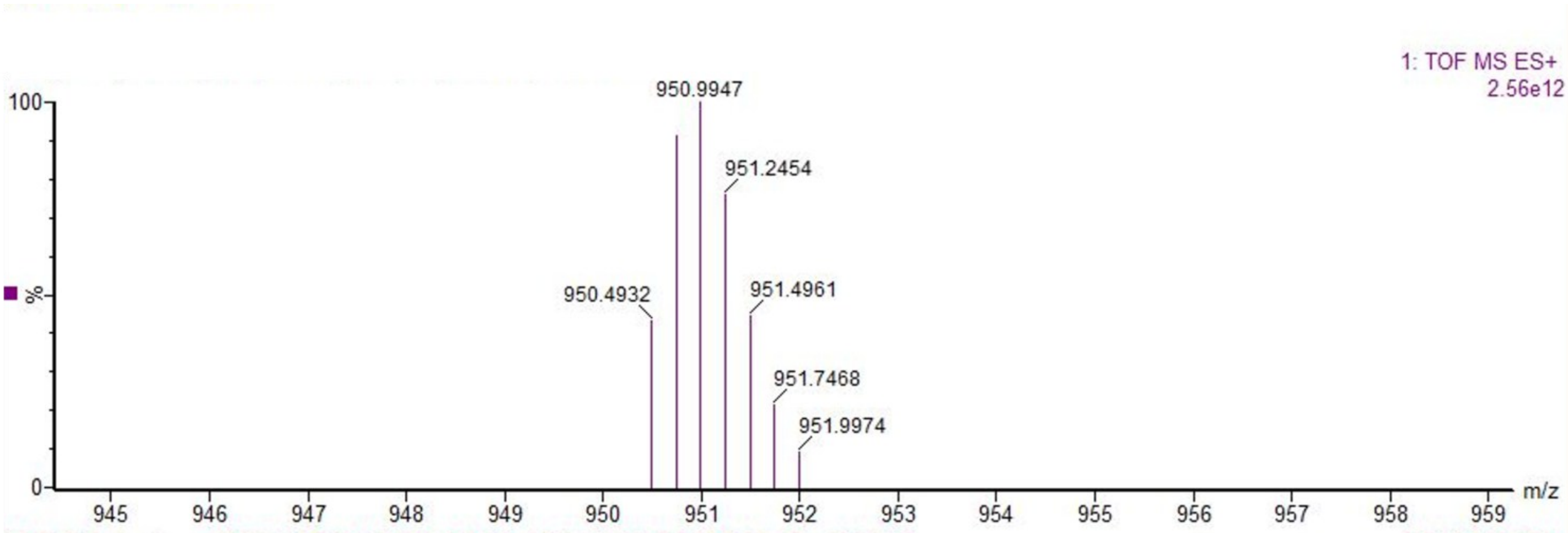

- ***Analytical HPLC (at 254 nm) and MS traces of GLP1-PROTAC 1***

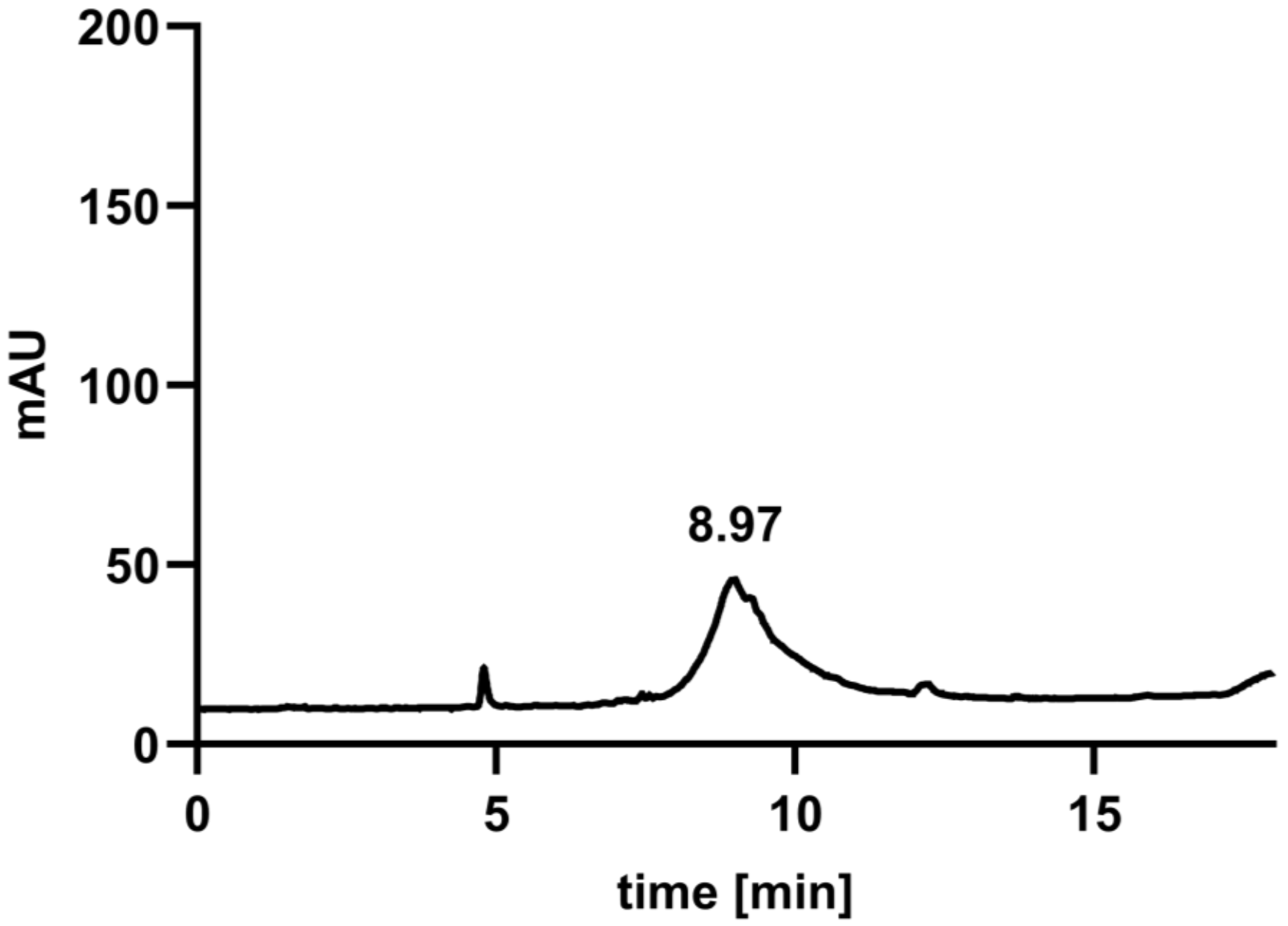

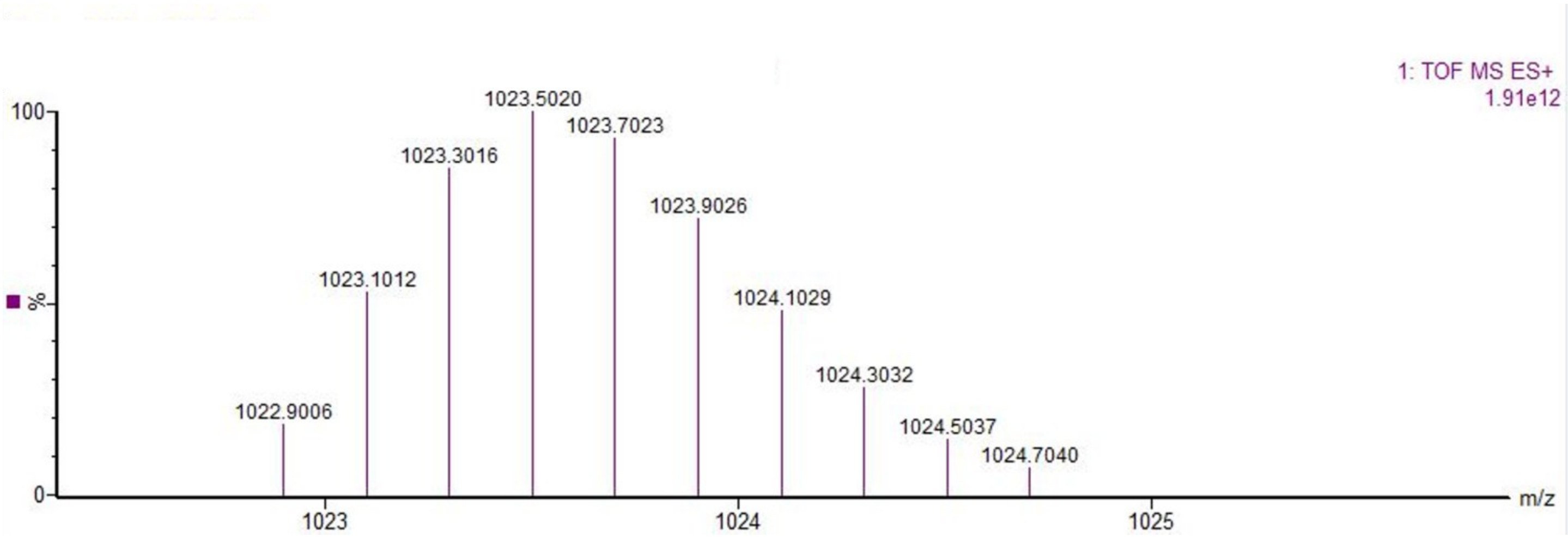

- ***UV LCMS (at 280 nm) of crude compounds S10–S12***

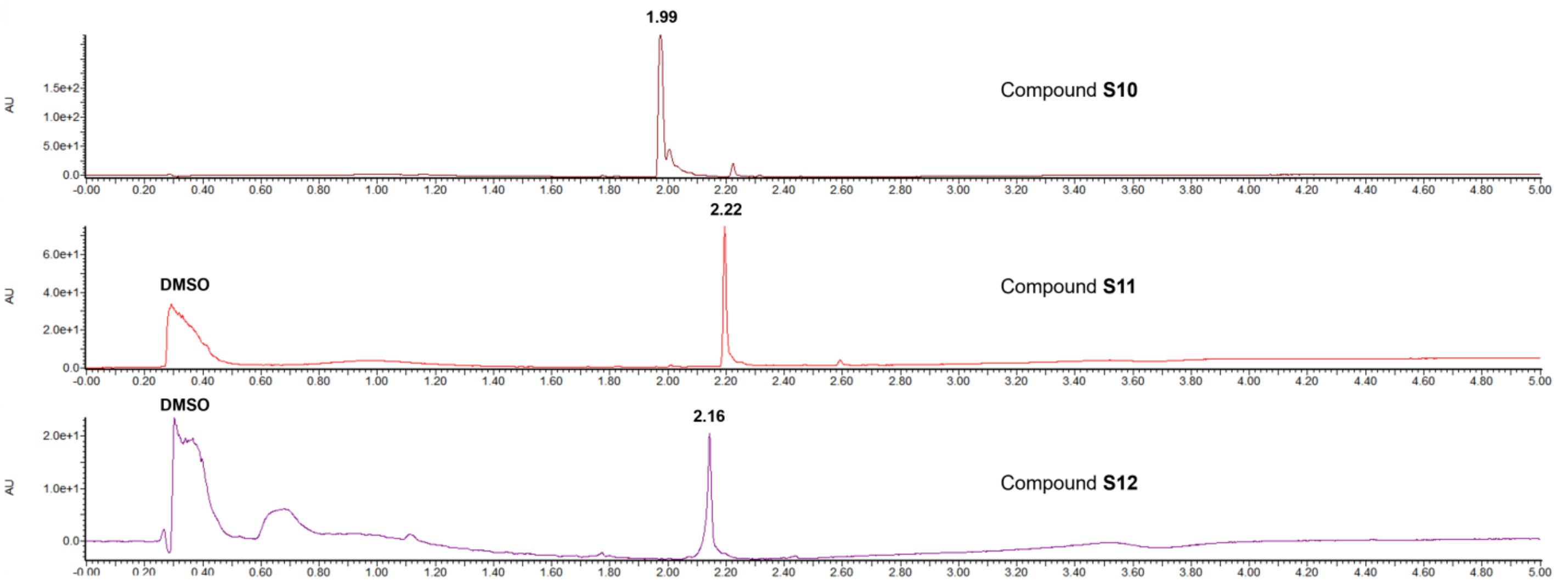

- ***UV LCMS (at 299 nm) and MS traces of S20***

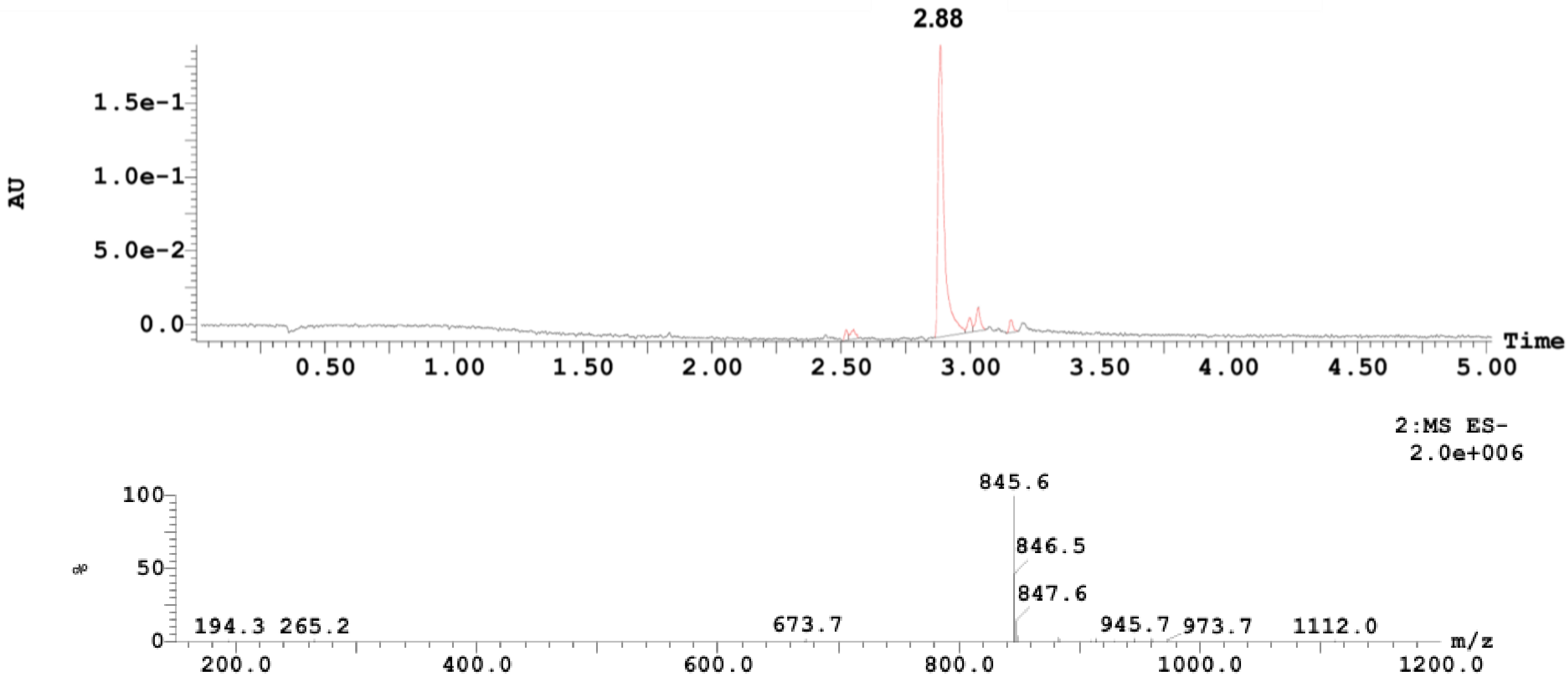

- ***UV LCMS (at 254 nm) and MS traces of GLP1-PROTAC 2***

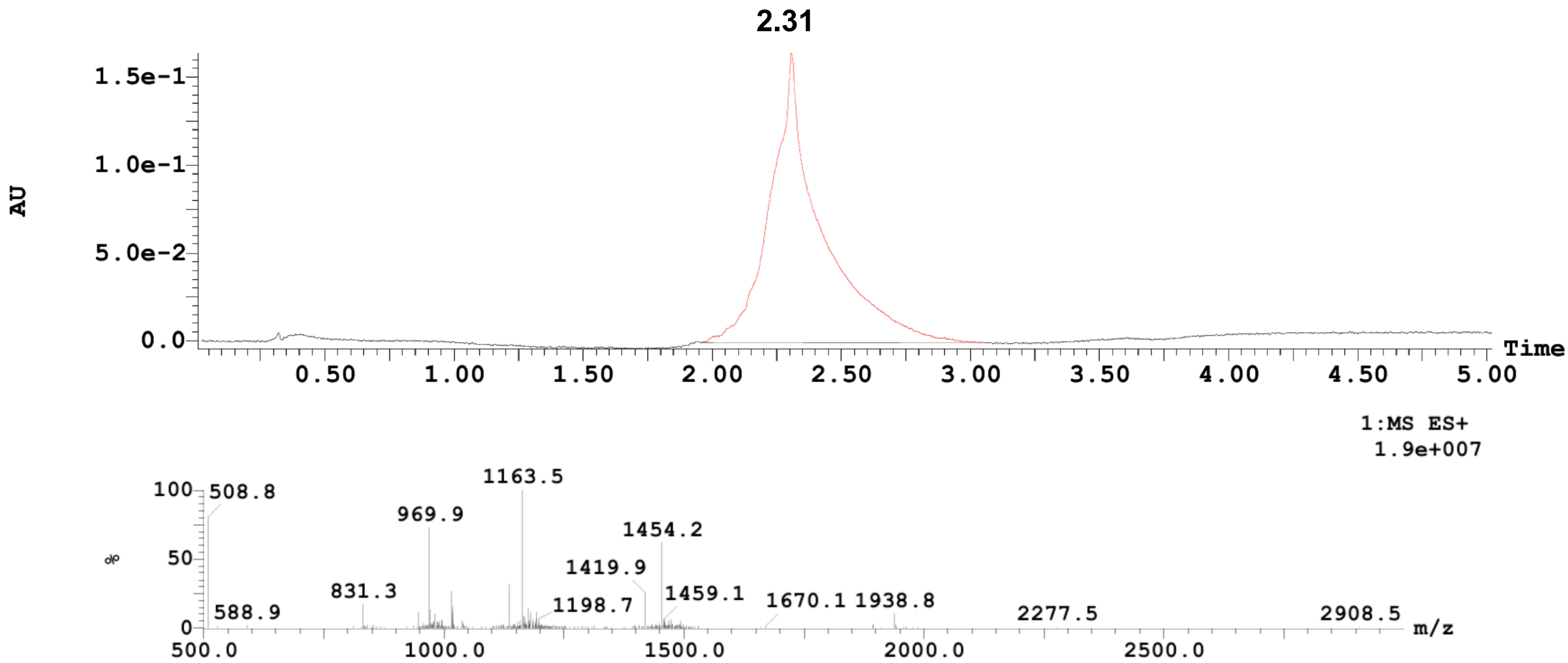

3. Biophysical and stability data

For PBS stability test, 10 mM stock solution of **11** in DMSO (6.56 mg per 500 μL of DMSO) has been prepared. Into the plastic Eppendorf tube, 95 μL of 1X PBS buffer (pH = 7.4) has been added, followed by addition of 5 μL of stock solution of **11**. The Eppendorf tube has been allocated into the thermal shaker with constant temperature kept at 37 °C. At defined intervals (24h, 48h), 50 μL of the reaction mixture was taken and quenched with 50 μL of a mixture of ethanol/MeCN (1:1 v/v). Caffeine was added as an internal standard (5 μL of a 5 mM stock solution in MeCN/H_2O 1:1) and reaction mixture was analysed using UV-LCMS. Percentage of intact **11** was monitored over 48 hours (calculated as the ratio of the peak area corresponding to the intact **11** to the area of the peak of caffeine). Data points represent two individual analytical measurements from a single experiment. Line is included to guide the eye. No statistical comparisons were performed.

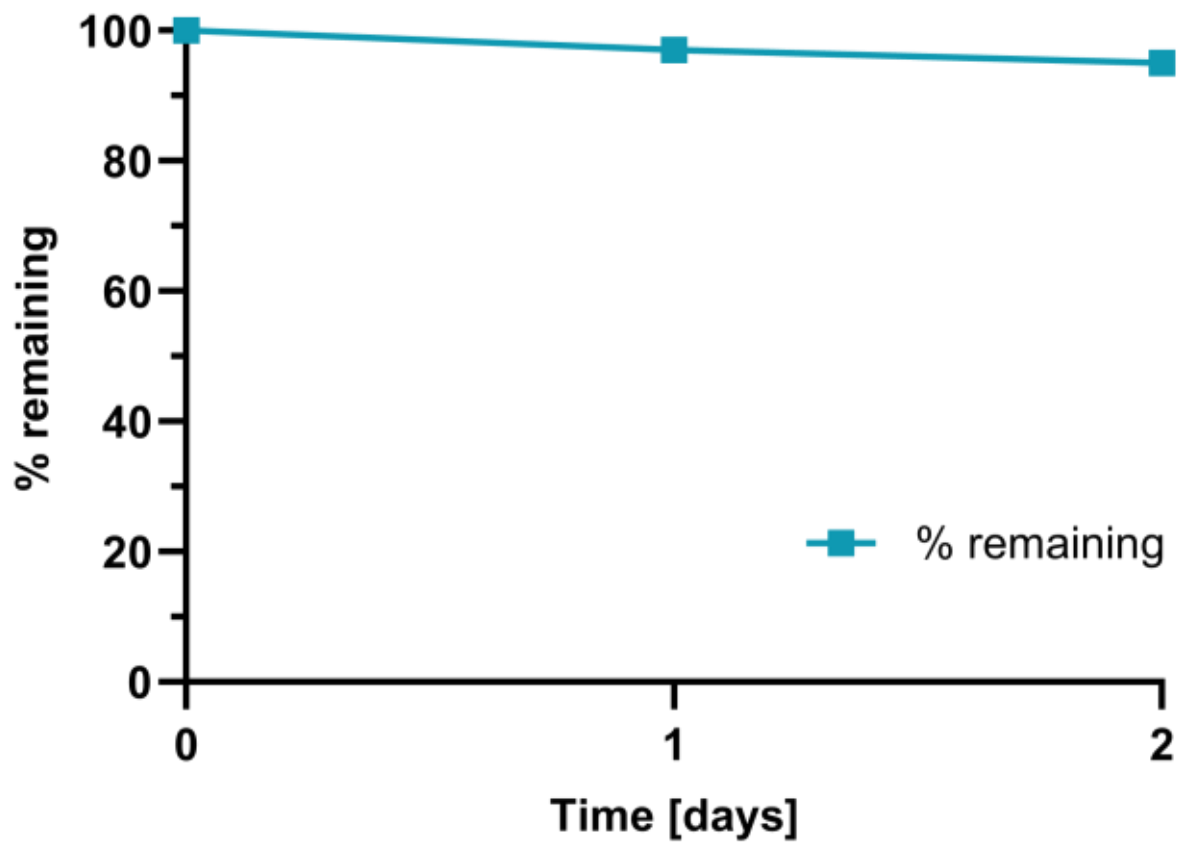

For esterase stability test, 10 mM stock solution of **11** in DMSO (6.56 mg per 500 μL of DMSO) has been prepared. Into the plastic Eppendorf tubes, 50 μL of pancreatin enzyme (25 mg in 1 mL 1X PBS buffer, pH = 7.4), 50 μL of PBS 1X and 5 μL 10 mM stock solution of **11** have been added. The Eppendorf tube has been allocated into the thermal shaker with constant temperature kept at 37 °C. At defined intervals (1h, 4h, 6h and 24h), the reaction mixture was taken and quenched with 200 μL of a mixture of ethanol/MeCN (1:1 v/v). Caffeine was added as an internal standard (5 μL of a 5 mM stock solution in MeCN/H_2O 1:1 v/v) and reaction mixture was analysed using analytical HPLC eluting with a linear gradient system 5 – 95% B (solvent A: 0.05% (v/v) TFA in H_2O, solvent B: 0.05% (v/v) TFA in MeCN) over 18 minutes, at a flow rate of 1 mL/min (5 μL injection volume). Percentage of **11** vs **9** was monitored over 24 hours (calculated as the ratio of the peak area corresponding to the intact **11** and newly formed **9** to the area of the peak of caffeine). Data points represent two individual analytical measurements from a single experiment. Lines are included to guide the eye. No statistical comparisons were performed.

For serum stability test of the TMPR-stapled peptide **8** and its linear precursor **S8** in human serum, 750 μL of PBS buffer supplemented with 250 μL of human serum (25% v/v) was allocated into an Eppendorf tube and temperature kept at 37 °C for 15 minutes before commencing the experiment. 20 μL of the peptide from 10 mM stock solution in DMSO was added. Caffeine was added as an internal standard (20 μL of a 10 mg/mL solution in distilled water). At defined intervals 50 μL of the reaction mixture was taken and quenched with 100 μL of a mixture of ethanol/DMSO (1:1 v/v). The suspension was spun at 8000 g for 4 minutes. 100 μL of the supernatant

was analysed using analytical HPLC eluting at a flow rate of 1 mL/min (80 µL injection volume). Percentage of intact starting peptide was monitored over 24 h (calculated as the ratio of the peak area corresponding to the intact peptide to the area of the peak of caffeine). Data points represent mean percentage of intact peptide remaining. Data are presented as mean ± SD from three independent serum-stability experiments (n = 3). Error bars represent SD. No statistical comparisons were performed.

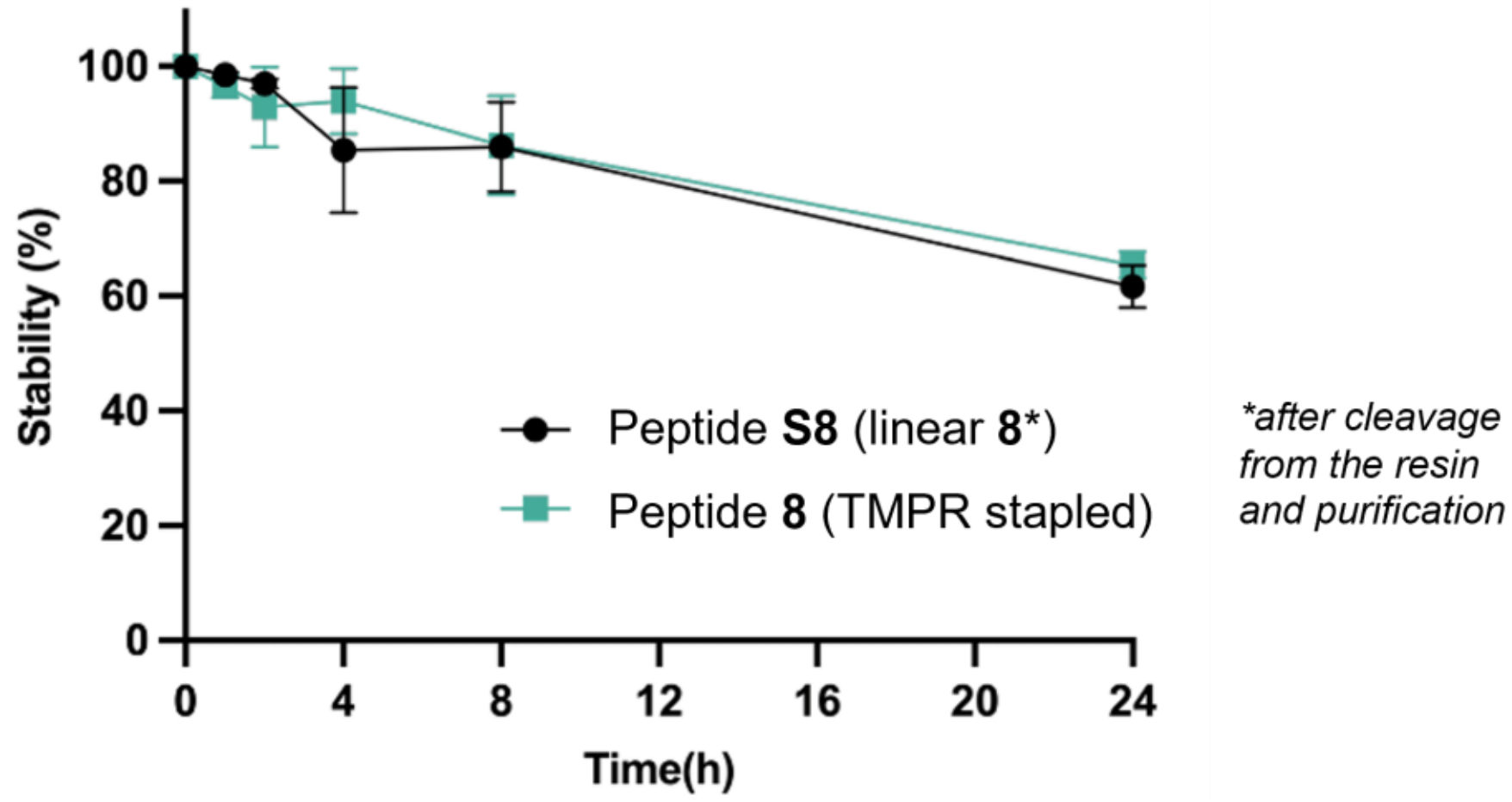

- ***Graphical representation of the cleavage sites in the parent hGLP-1 (7-37) peptide rationalising the observed stability as several protease-sensitive positions remain outside the stapled region of peptide 8***

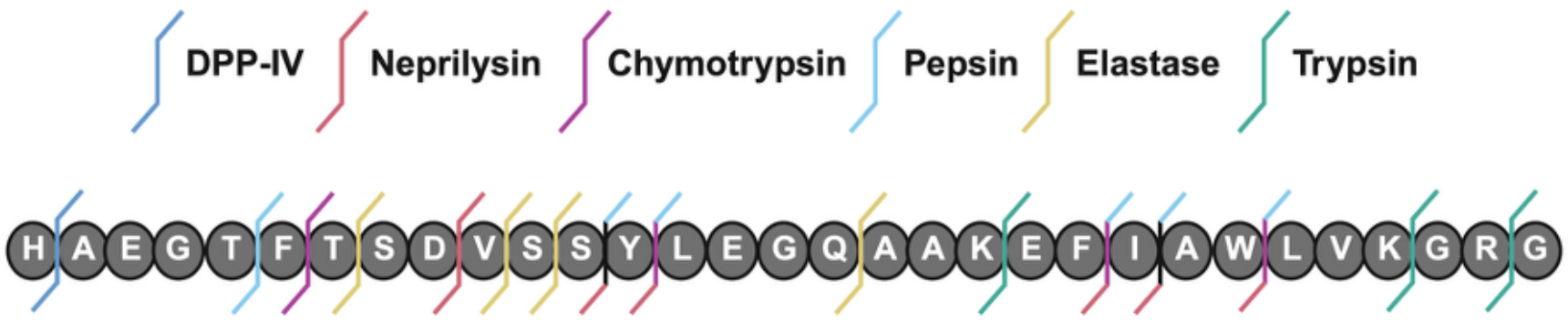

Circular dichroism spectra were obtained on an Aviv Model 410 CD spectrometer at 25 °C using a 1 mm path length, scanning between 250 and 195 nm. Peptides were dissolved in distilled water without adjusting of pH. **Helical content** has been calculated using Bestsel software as follows:

- ***Peptide hGLP-1***

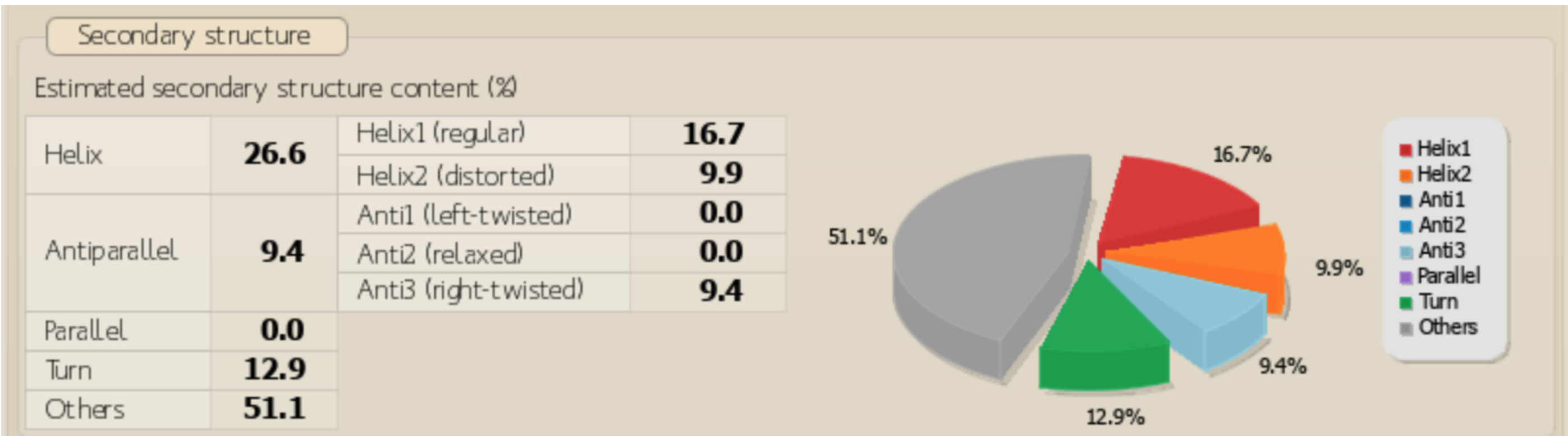
Secondary structure

Estimated secondary structure content (%)

Helix	26.6	Helix1 (regular)	16.7
		Helix2 (distorted)	9.9
Antiparallel	9.4	Anti1 (left-twisted)	0.0
		Anti2 (relaxed)	0.0
		Anti3 (right-twisted)	9.4
Parallel	0.0		
Turn	12.9		
Others	51.1		

▪ ***Peptide hGLP-1 A8Aib***

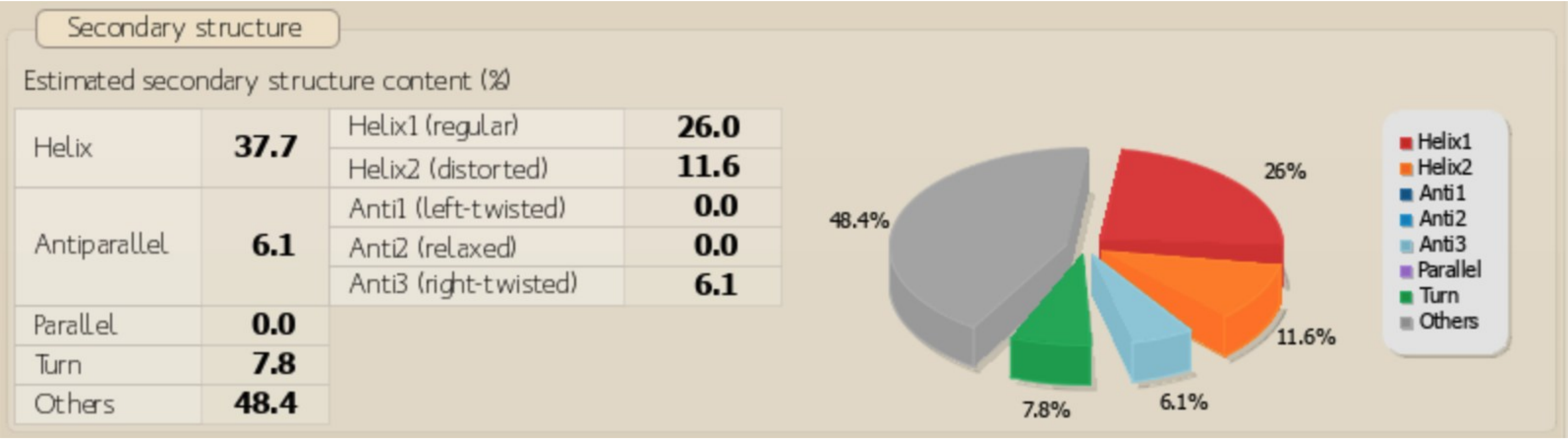
Secondary structure

Estimated secondary structure content (%)

Helix	37.7	Helix1 (regular)	26.0
		Helix2 (distorted)	11.6
Antiparallel	6.1	Anti1 (left-twisted)	0.0
		Anti2 (relaxed)	0.0
		Anti3 (right-twisted)	6.1
Parallel	0.0		
Turn	7.8		
Others	48.4		

▪ ***Peptide 8***

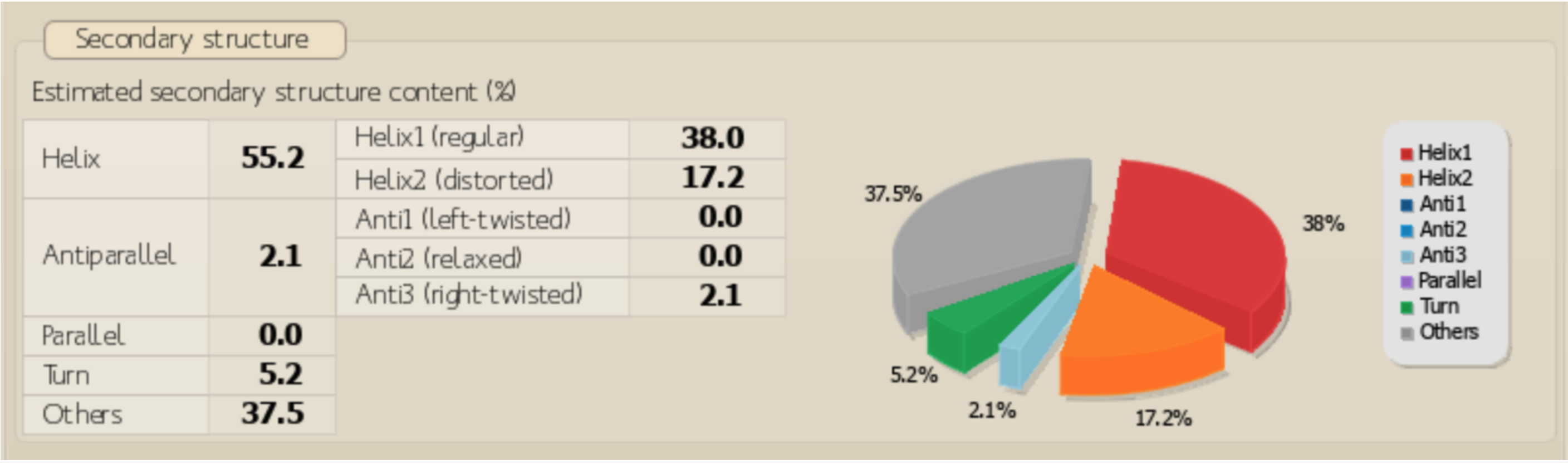
Secondary structure

Estimated secondary structure content (%)

Helix	55.2	Helix1 (regular)	38.0
		Helix2 (distorted)	17.2
Antiparallel	2.1	Anti1 (left-twisted)	0.0
		Anti2 (relaxed)	0.0
		Anti3 (right-twisted)	2.1
Parallel	0.0		
Turn	5.2		
Others	37.5		

4. Biological Evaluation of Compounds 1–8 and GLP1-PROTACs

4.1. Cell Culture

CHO-GLP1R, CHO-GCGR, and CHO-GIPR cells were cultured at 37˚C, 95% humidity, 5% CO_2 in DMEM /F-12 (Gibco, #311320033) supplemented with 10% FBS (Gibco, #A5256701), 400 µg/mL Hygromycin B (Gibco, # 10687010), and 500 µg/mL Geneticin (Gibco, #10131035). Cells were cryopreserved at a density of 1×10^6 cells/mL in medium supplemented with 10% DMSO.

4.2. cAMP accumulation

Compound activity levels were assessed by quantifying intracellular cAMP levels following receptor activation. cAMP accumulation was measured with the HTRF cAMP Gs Dynamic Detection kit (Revvity, 62AM4PEB) in CHO cells stably overexpressing human GLP-1R, GCGR, or GIPR. The assay relies on a Förster resonance energy transfer (FRET) signal generated by the binding of a monoclonal cAMP Europium Cryptate-labelled antibody (Europium donor) to a d2-labeled cAMP (red acceptor). Upon receptor activation, endogenous cAMP is generated and binds to the antibody, resulting in a loss of signal. First, incubation buffer was prepared from HBSS (Gibco, #14025092) supplemented with 20 mM HEPES (pH 7.4) (Gibco, #15630080), 0.1% BSA (Sigma-Aldrich, #A9418), and 0.5 mM IBMX (Sigma-Aldrich, #410957). Next, compounds **1–8** and their respective controls were diluted in incubation buffer and dispensed onto a white 384-well low-volume plate (Greiner, #4513) using a Labcyte ECHO (non-acoustic dispenser) for a final well volume of 5 µL. Afterwards, CHO-GLP1R, CHO-GCGR, or CHO-GIPR cell vials were defrosted and their medium removed. 5 µL per well of cell suspension

containing either 1000 CHO-GLP1R, CHO-GCGR or 500 CHO-GIPR cells were dispensed into the 384-well low-volume plate prepared earlier. The test compounds and cells were then incubated for 30 minutes at room temperature. The assay was performed according to the manufacturer's protocol with an incubation period of 60 minutes. A Pherastar Plus (BMG) plate reader was used to measure acceptor (665 nm) and donor (620 nm) emissions. To analyse the data, the ratio of 665 nm/620 nm * 10^4 was calculated for each well. Afterwards, the delta F (%) was calculated using the formula: Delta F (%) = (Ratio sample – Ratio Negative Control)/Ratio Negative Control × 100. The negative control wells, representing basal cAMP levels, contained cells, incubation buffer, monoclonal cAMP Europium Cryptate-labelled antibody, and lysis buffer. The resulting data was analysed with a four-parameter logistical analysis in Prism and the EC_{50} values for each sample were calculated.

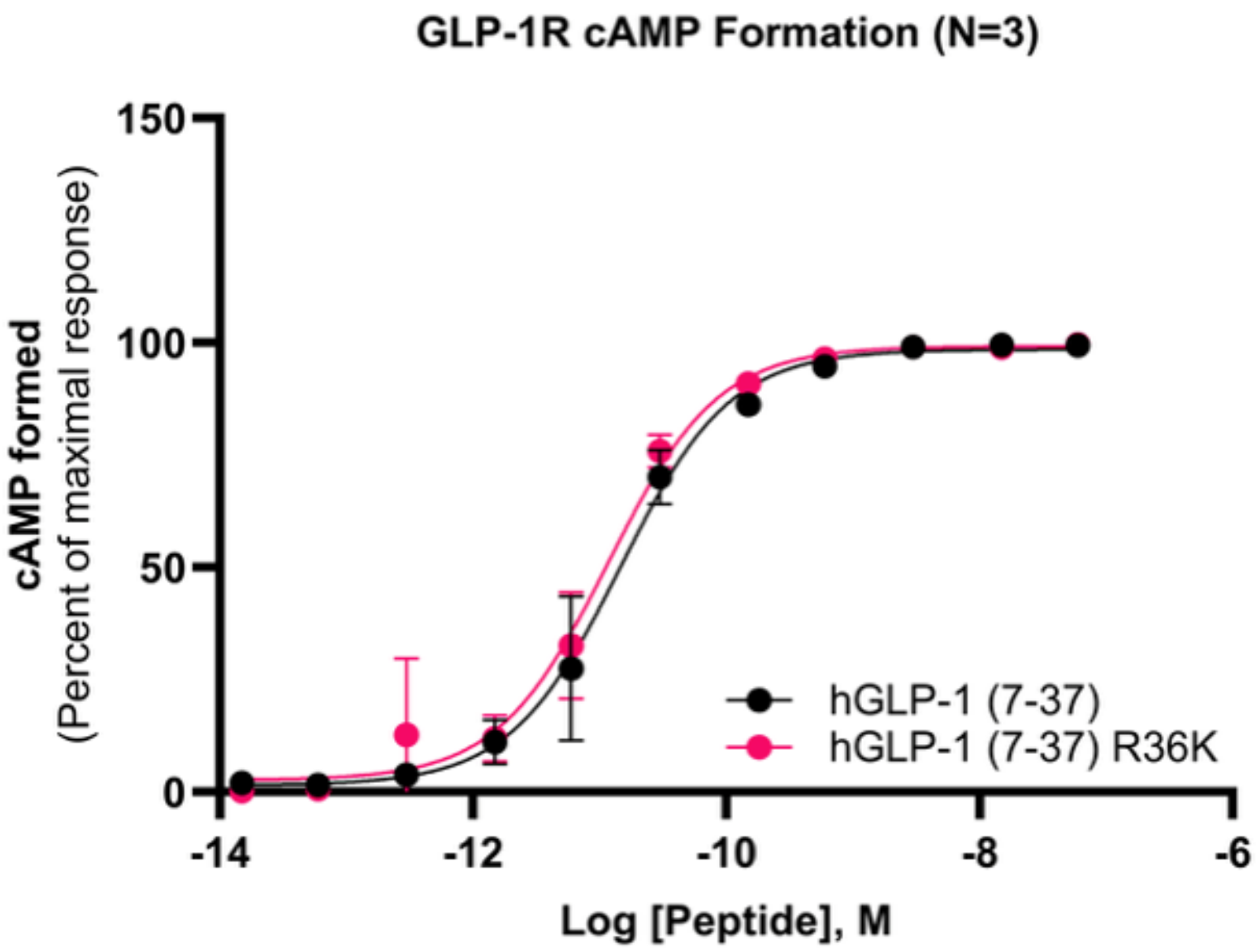

Figure S1. Concentration–response curves for hGLP-1(7–37) and hGLP-1(7–37) R36K in the GLP-1R-mediated cAMP accumulation assay. Data points represent mean cAMP formation normalised to the maximal response of **hGLP-1(7–37).** Data are presented as mean ± SD from three independent experiments (n = 3), each performed in technical triplicate. Curves were fitted by nonlinear regression using a four-parameter logistic model. No statistical comparisons were performed. cAMP = 3′,5′-cyclic adenosine monophosphate; GLP-1R = glucagon-like peptide-1 receptor.

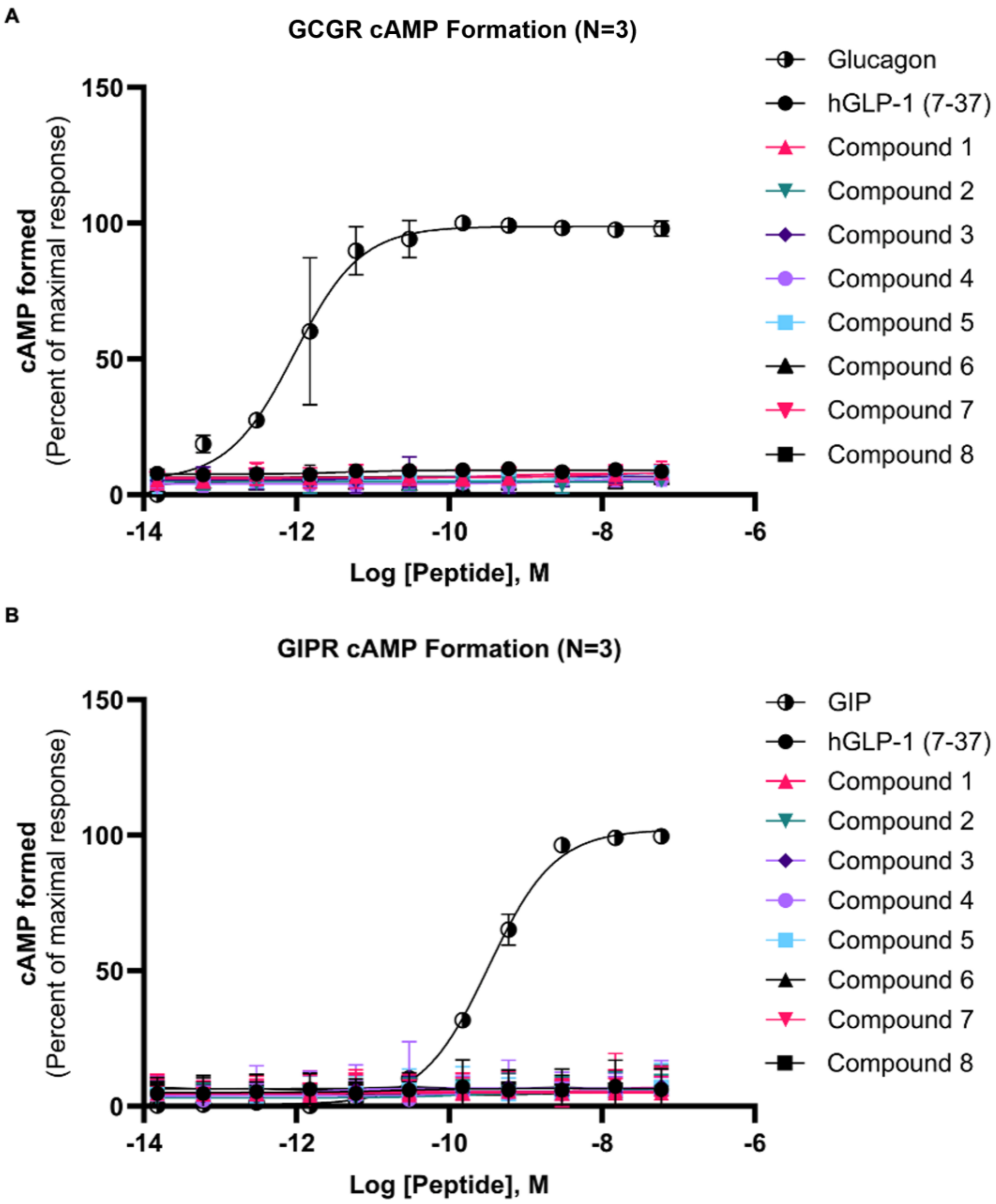

Figure S2. Peptides 1–8 maintain selectivity for GLP-1R and do not activate GCGR and GIPR. A) GCGR-mediated cAMP production; human glucagon served as a positive control. **B)** GIPR mediated cAMP production; human GIP served as positive control. Data points represent mean cAMP formation normalised to the maximal response of the respective positive control. Data are presented as mean ± SD from three independent experiments (n = 3), each performed in technical triplicate. Curves were fitted by nonlinear regression using a four-parameter logistic model where applicable. No statistical comparisons were performed. *Abbreviations:* cAMP = 3′,5′-cyclic adenosine monophosphate; GCGR = glucagon receptor; GIPR = glucose-dependent insulinotropic polypeptide receptor

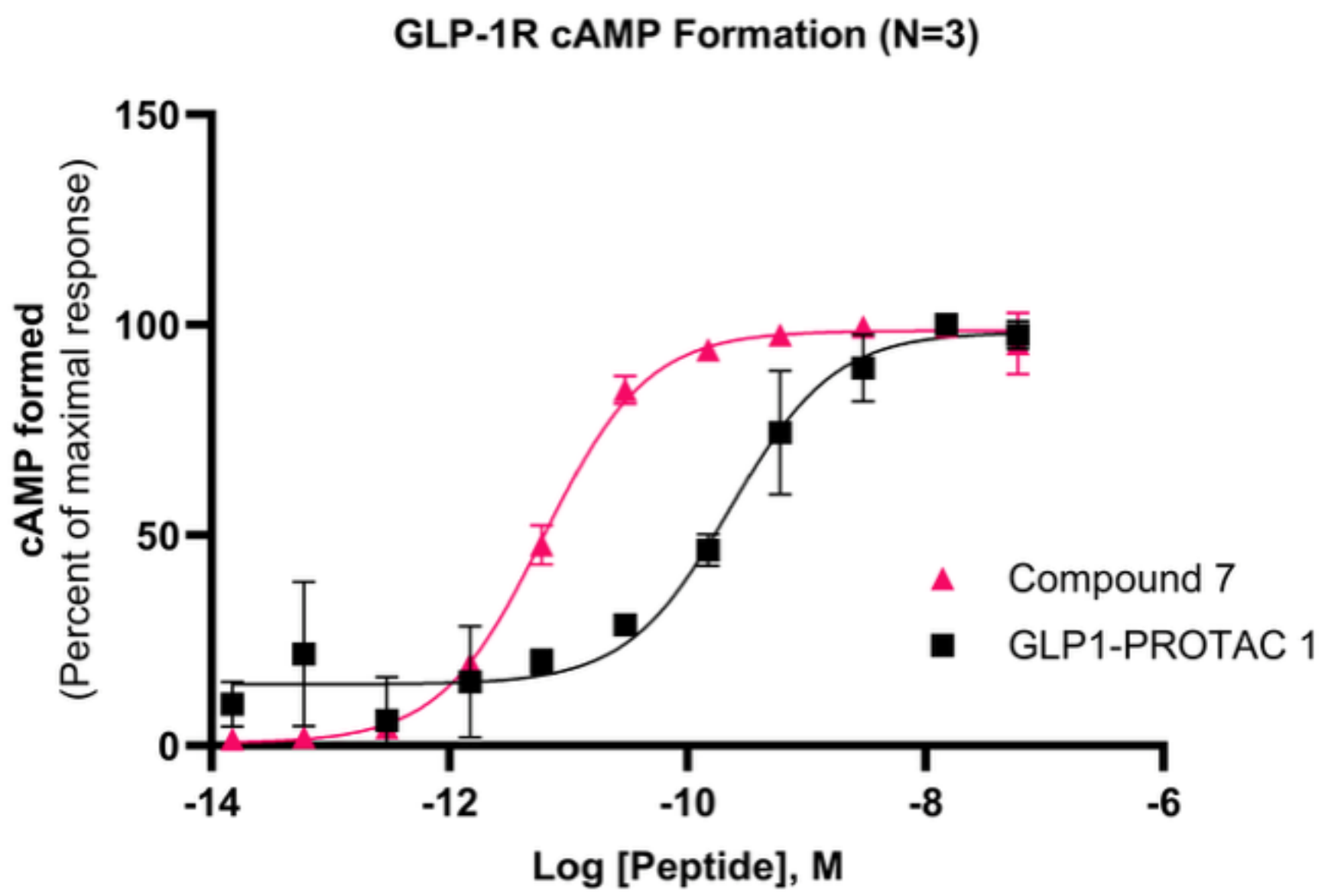

Figure S3. GLP1-PROTAC 1 retains potent GLP-1R activity. GLP1-PROTAC 1 (EC_{50} = 223 pM) exhibited a modest reduction in potency relative to compound 7 but remained highly active at GLP-1R. Data points represent mean cAMP formation normalised to the maximal response of compound **7**. Data are presented as mean ± SD from three independent experiments (n = 3), each performed in technical triplicate. Curves were fitted by nonlinear regression using a four-parameter logistic model. No statistical comparisons were performed. *Abbreviations:* EC_{50} = half-maximal effective concentration; cAMP = 3′,5′-cyclic adenosine monophosphate; GLP-1R = glucagon-like peptide-1 receptor.

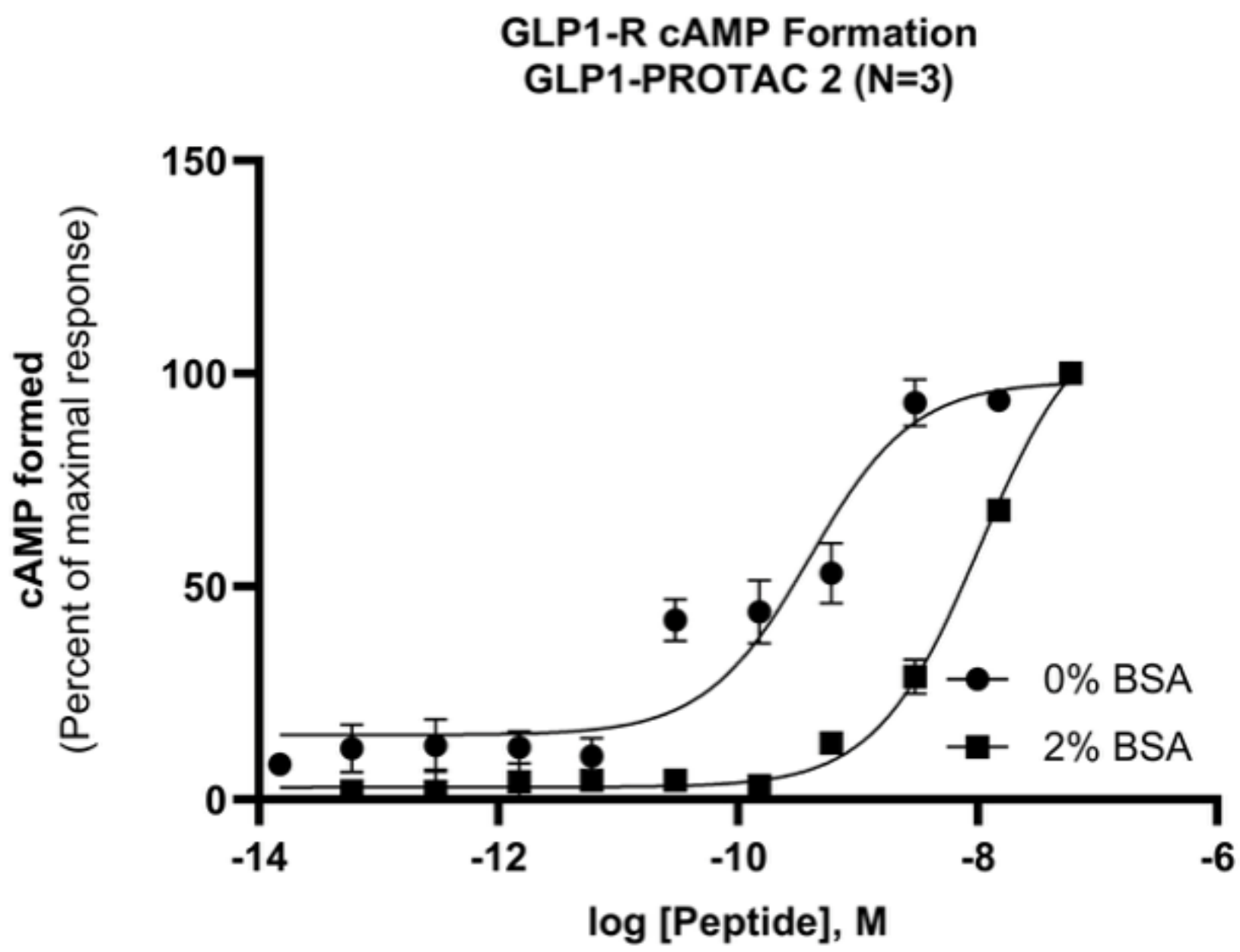

Figure S4. GLP1-PROTAC 2 binds albumin. Concentration–response curves for **GLP1-PROTAC 2** in the GLP-1R-mediated cAMP accumulation assay in the absence or presence of 2% BSA. Addition of 2% BSA reduced the apparent potency of **GLP1-PROTAC 2** from 391 pM to 1004 pM, consistent with a lower free compound concentration due to albumin binding. Data points represent mean cAMP formation normalised to the maximal **GLP1-PROTAC 2** response. Data are presented as mean ± SD from three independent experiments (n = 3),

each performed in technical triplicate. Curves were fitted by nonlinear regression using a four-parameter logistic model. No statistical comparisons were performed. *Abbreviations:* BSA = Bovine Serum Albumin; cAMP = 3′,5′-cyclic adenosine monophosphate; GLP-1R = glucagon-like peptide-1 receptor;

4.3. Western Blot

To assess the ability of **GLP1-PROTAC 1** and **GLP1-PROTAC 2** to induce BRD4 degradation, Western blot analysis was performed. Chinese hamster ovary (CHO) cells overexpressing GLP-1R were used as the model system, with wild-type CHO cells serving as a negative control to differentiate specific receptor-mediated internalization from non-specific uptake. CHO-GLP1R and wild-type CHO cells were seeded at a density of 300,000 cells/well in 6-well clear flat bottom plate (Corning, #353046) and incubated for 24 hours without FBS. The cells were then treated with **GLP1-PROTAC 1** and **GLP1-PROTAC 2** at concentrations of 10 µM, 5 µM, 1 µM, 0.5 µM, and 0.1 µM. Following a 24-hour incubation, cells were harvested and spun down at 300 g. The resulting cell pellet was resuspended in 1 mL of PBS, spun down again at 300 g, and the supernatant was aspirated. The pellet was then placed on ice.

For cell lysis, a RIPA buffer (Thermo Scientific, #89900) containing protease inhibitor (Thermo Scientific, #78440) was prepared. Pellets were resuspended in 100-200 µL of the buffer and incubated on ice for 15 minutes. Lysates were then centrifuged at maximum speed for 10 minutes at 4˚C, and the supernatant was transferred to a new tube. Samples containing 30 µg of protein were denatured by boiling at 90˚C for 10 minutes. The denatured samples were loaded onto a NuPAGE™ Tris-Acetate 3-8% gel (Invitrogen™, #WG1601BX10). Electrophoresis was performed at 150V for 80 minutes. A dry transfer was then conducted using an iBlot 3 system (Invitrogen™, #IB31001), which was run for 7 minutes at 25V according to the manufacturer's protocol, with a PVDF membrane (Invitrogen™, #IB34001). Following the transfer, the membrane was incubated with 50 mL of Intercept® (TBS) blocking buffer (LicorBio, #927-60001) for 30 minutes with shaking at room temperature. The membrane was then incubated overnight with a primary antibody, rabbit anti-BRD4 antibody (Abcam, Catalog #ab245285), at a 1:1,000 dilution in Intercept® (TBS) blocking buffer with 0.1% Tween. Mouse anti-Vinculin (Sigma, #V9131) was used as a loading control, at a dilution of 1:10,000. Following this, the membrane was washed three times for 10 minutes each in 1X TBS containing 0.01% Tween. The secondary antibodies, goat anti-mouse (LicorBio, #926-68070) and goat anti-rabbit (LicorBio, #926-32211), were diluted to 1:10,000 in Intercept® (TBS) blocking buffer with 0.1% Tween and incubated with the membrane for 45 minutes at room temperature. The membrane was then washed three times for 5 minutes with 1X TBS with 0.1% Tween, followed by a final wash with 1X TBS only to reduce background signal. Finally, the membrane was imaged using a Li-Cor Odyssey Fc machine and analysed with ImageStudio Lite software.

- ***Uncropped Western Blot of GLP1-PROTAC 1 for Figure 3A***

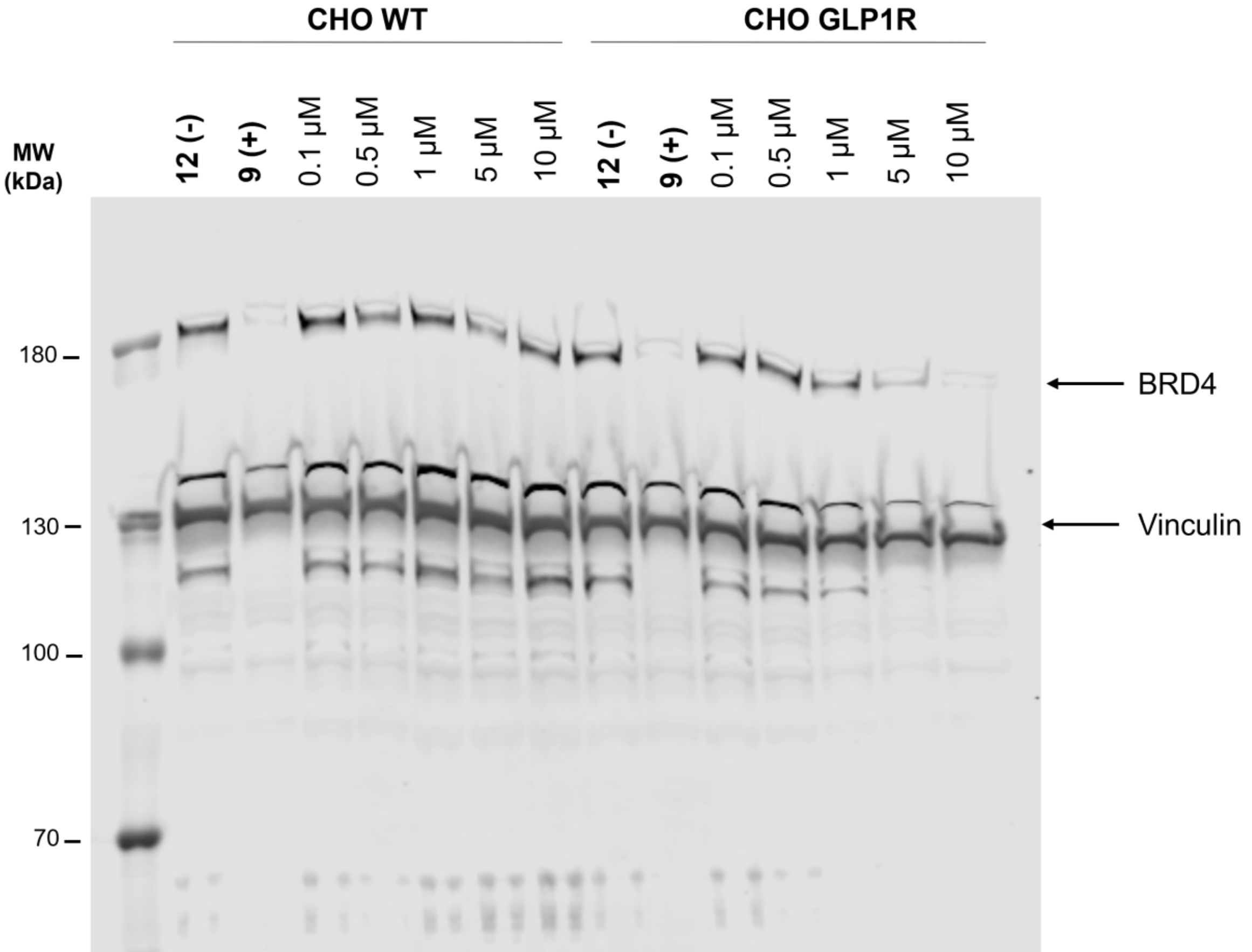

- ***Uncropped Western Blot of GLP1-PROTAC 2 for Figure 3A***

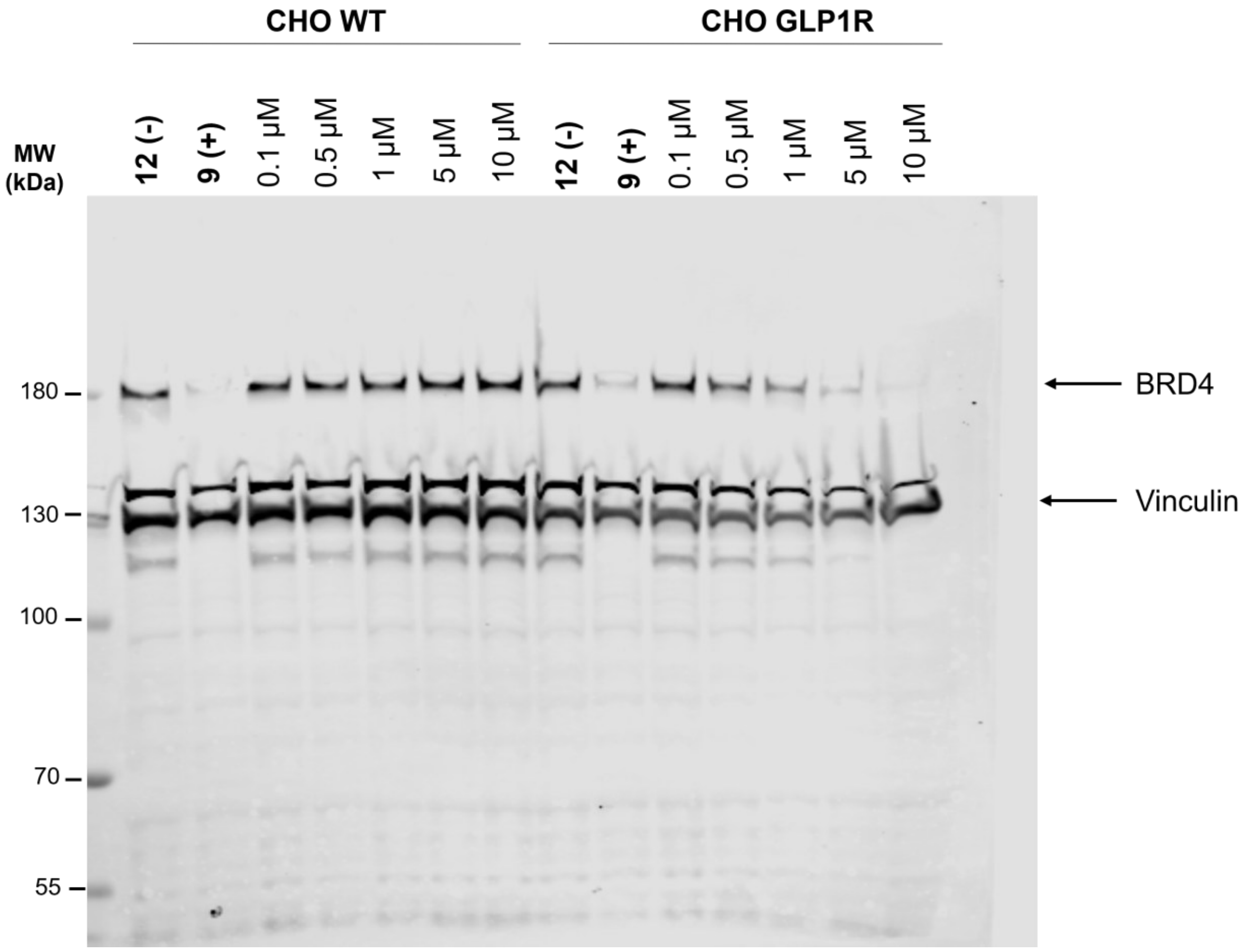

4.4. CellTiter-Glo® 2.0 Cell Viability Assay

The viability of GLP1R-CHO cells in the presence of compounds 1 – 8 was assessed using a Promega CellTiter-Glo® 2.0 cell viability assay (Promega, #G9241). The assay estimates the number of metabolically active cells by quantifying the amount of ATP; it generates a luminescent signal proportional to the ATP present after cell lysis. This reaction is catalysed by the Ultra-Glo™ luciferase, which converts Luciferin to Oxyluciferin in the presence of ATP. GLP1R-CHO cells were seeded into a 384-well white assay plate (Greiner, #781080) at a density of 3,000 cells per well in 30uL of media. A 10-point, 4X dilution series of compounds 1 – 8 was in a 384-well dilution plate (Greiner Bio-One, #781270). Doxorubicin was used as a positive control, 0.1% DMSO was used as a negative control. 10uL of diluted compound was then added to the assay plate, which was spun down at 1200rpm for 5min at room temperature. Following this, the plate was incubated at 37˚C, 5% CO_2 in a humidified incubator for 96 hours. After the incubation period, the CellTiter-Glo® 2.0 assay was carried out according to the manufacturer's protocol. A Pherastar Plus (BMG) plate reader was used to the luminescent signal, using an integration time of 0.25 to 1 seconds per well. The resulting data was analysed with a four-parameter logistical analysis in Prism and the IC_{50} values for each sample were calculated.

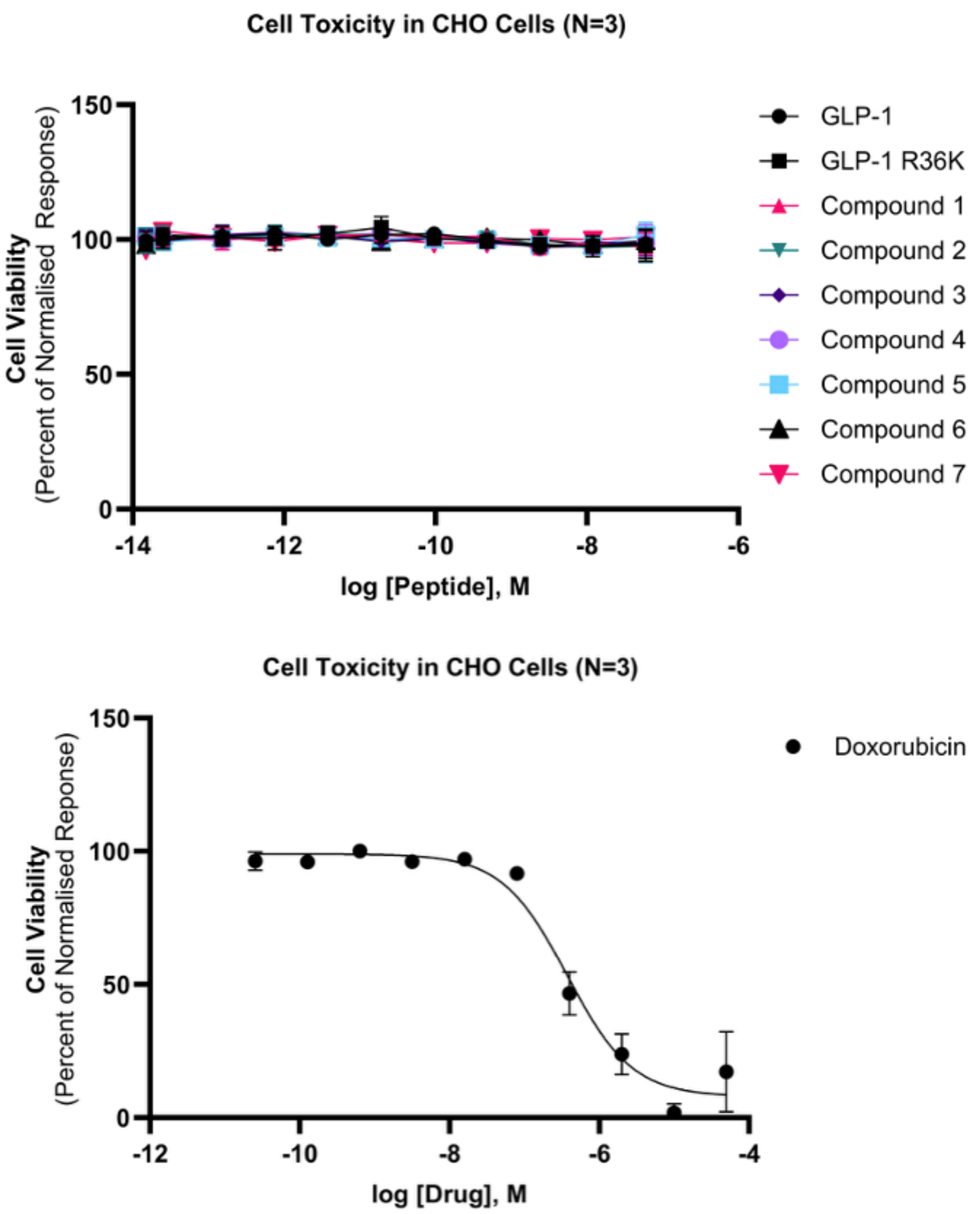

Figure S5. Peptides 1–7 are not cytotoxic to GLP-1R CHO cells. Cell viability of GLP-1R CHO cells after treatment with peptides **1–7**, **hGLP-1 R36K** and **GLP-1**, assessed using the CellTiter-Glo® 2.0 viability assay. GLP-1 was used as a negative control and doxorubicin as a positive cytotoxicity control. Data points represent

mean cell viability normalised to untreated/control cells. Data are presented as mean ± SD from three independent experiments (n = 3), each performed in technical triplicate. Error bars represent SD and may be smaller than the symbols. Curves were fitted by nonlinear regression using a four-parameter logistic model where applicable. No statistical comparisons were performed. *Abbreviations:* R36K = arginine-to-lysine substitution at position 36.

5. NMR spectra

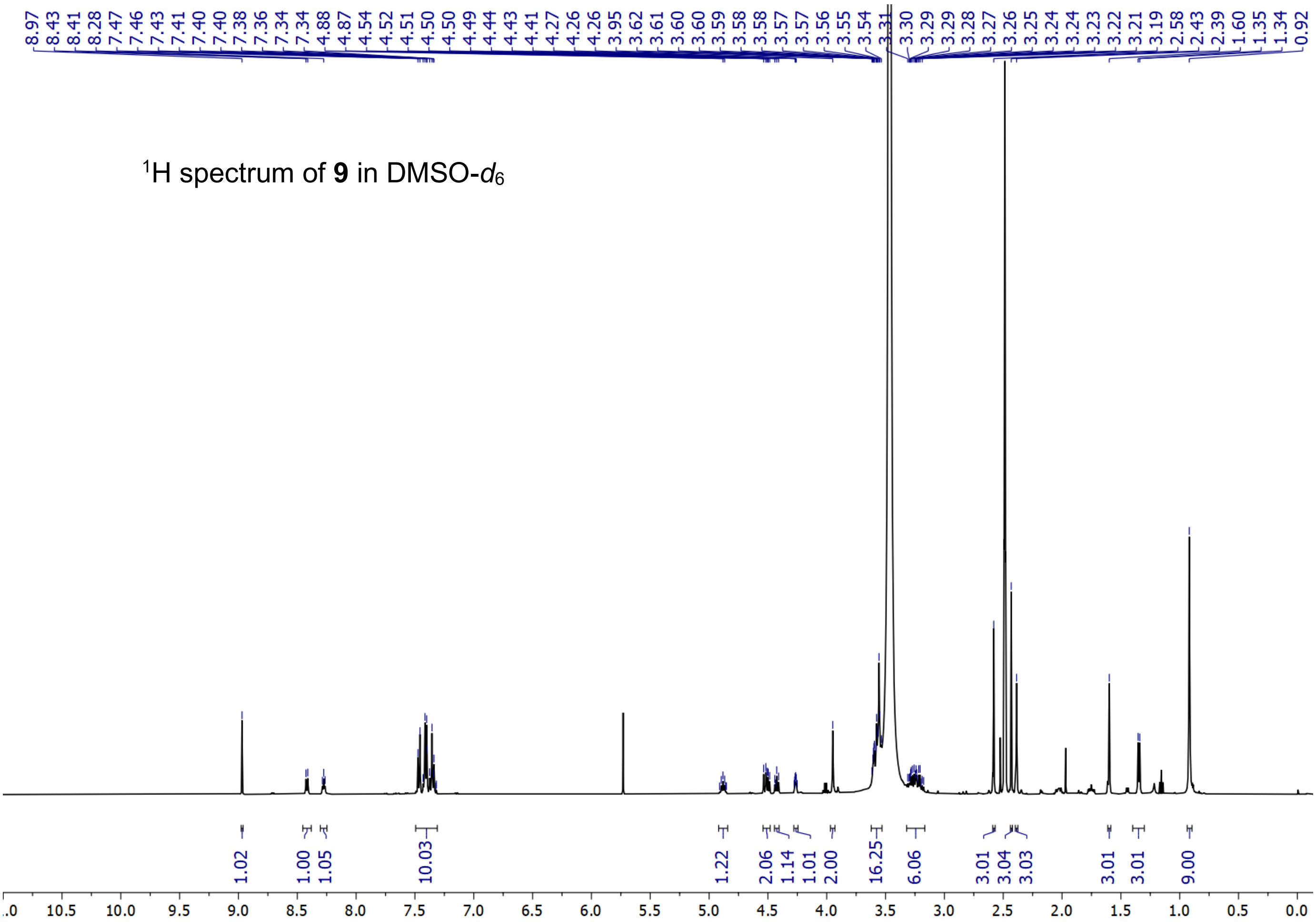

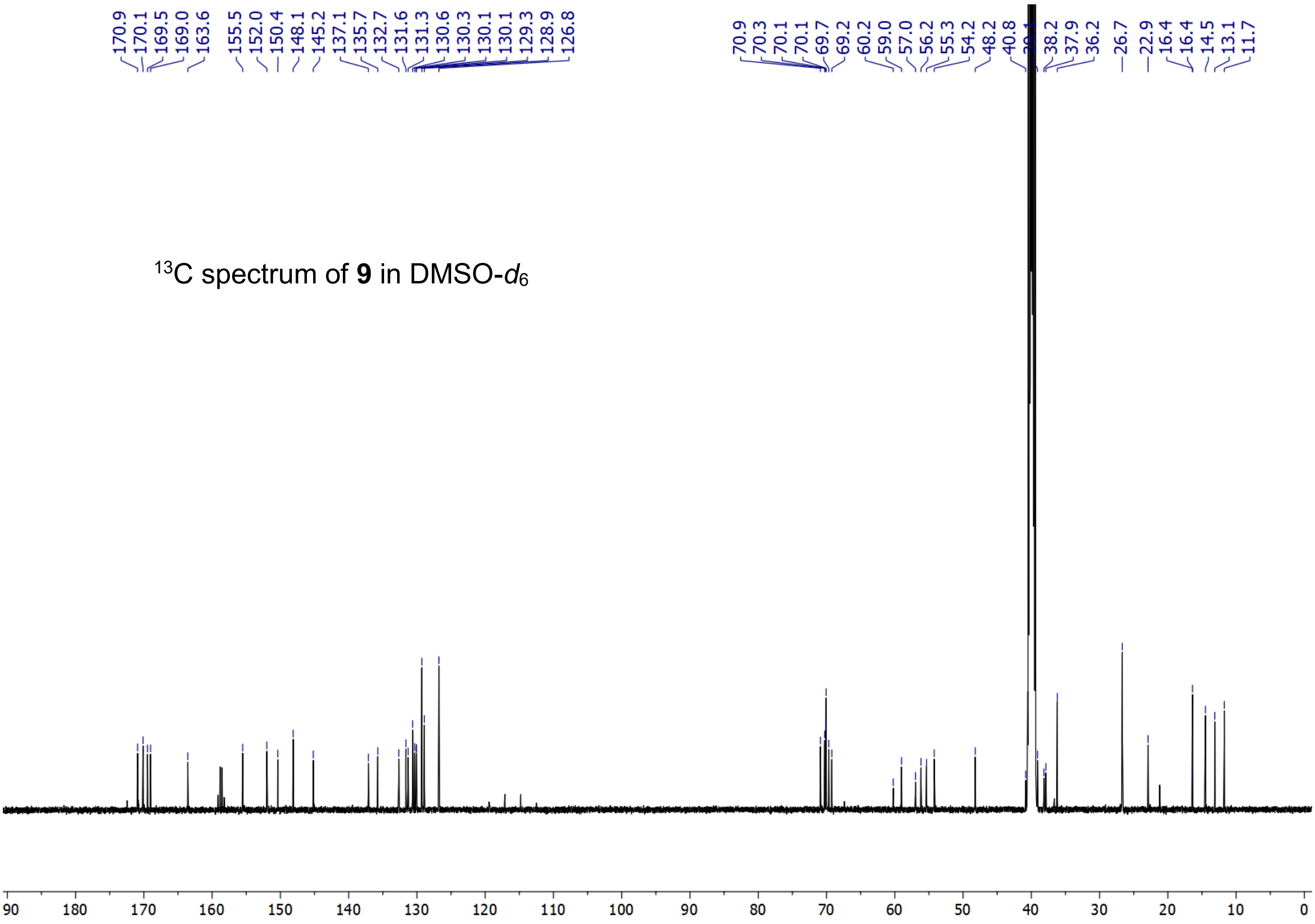

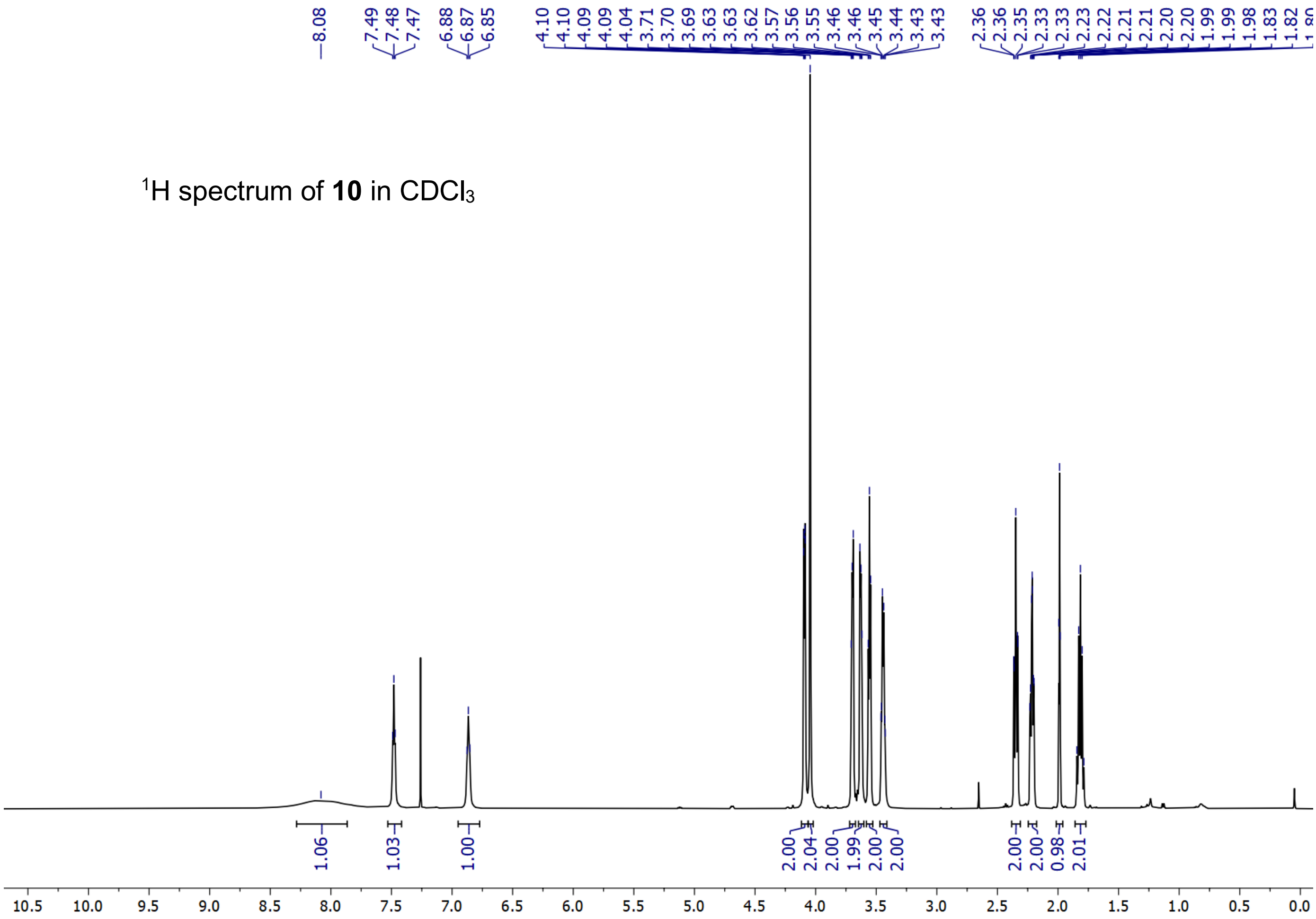

1H spectrum of 10 in CDCl3
8.08
7.49 7.48 7.47
6.88 6.87 6.85
4.10 4.10 4.09 4.09 4.04 3.71 3.70 3.69 3.63 3.63 3.62 3.57 3.56 3.55 3.46 3.45 3.44 3.43 3.43
2.36 2.36 2.35 2.33 2.33 2.23 2.22 2.21 2.21 2.20 2.20 1.99 1.99 1.98 1.83 1.82
1.06 1.03 1.00 2.00 2.04 2.00 1.99 2.00 2.00 2.00 2.00 0.98 2.01
10.5 10.0 9.5 9.0 8.5 8.0 7.5 7.0 6.5 6.0 5.5 5.0 4.5 4.0 3.5 3.0 2.5 2.0 1.5 1.0 0.5 0.0

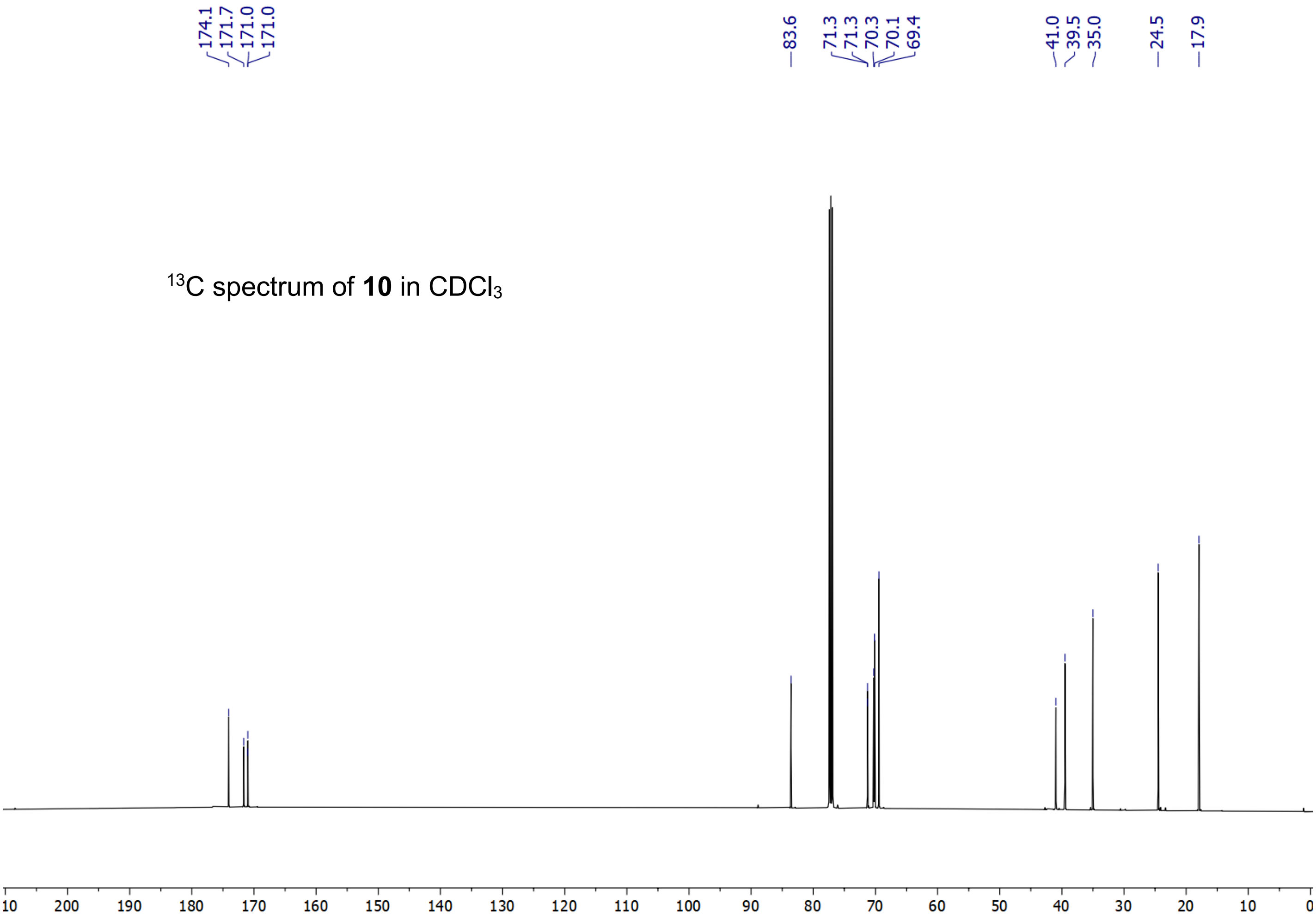

174.1 171.7 171.0 171.0
83.6
71.3 71.3 70.3 70.1 69.4
41.0 39.5 35.0
24.5
17.9
13C spectrum of 10 in CDCl3
10 200 190 180 170 160 150 140 130 120 110 100 90 80 70 60 50 40 30 20 10 0

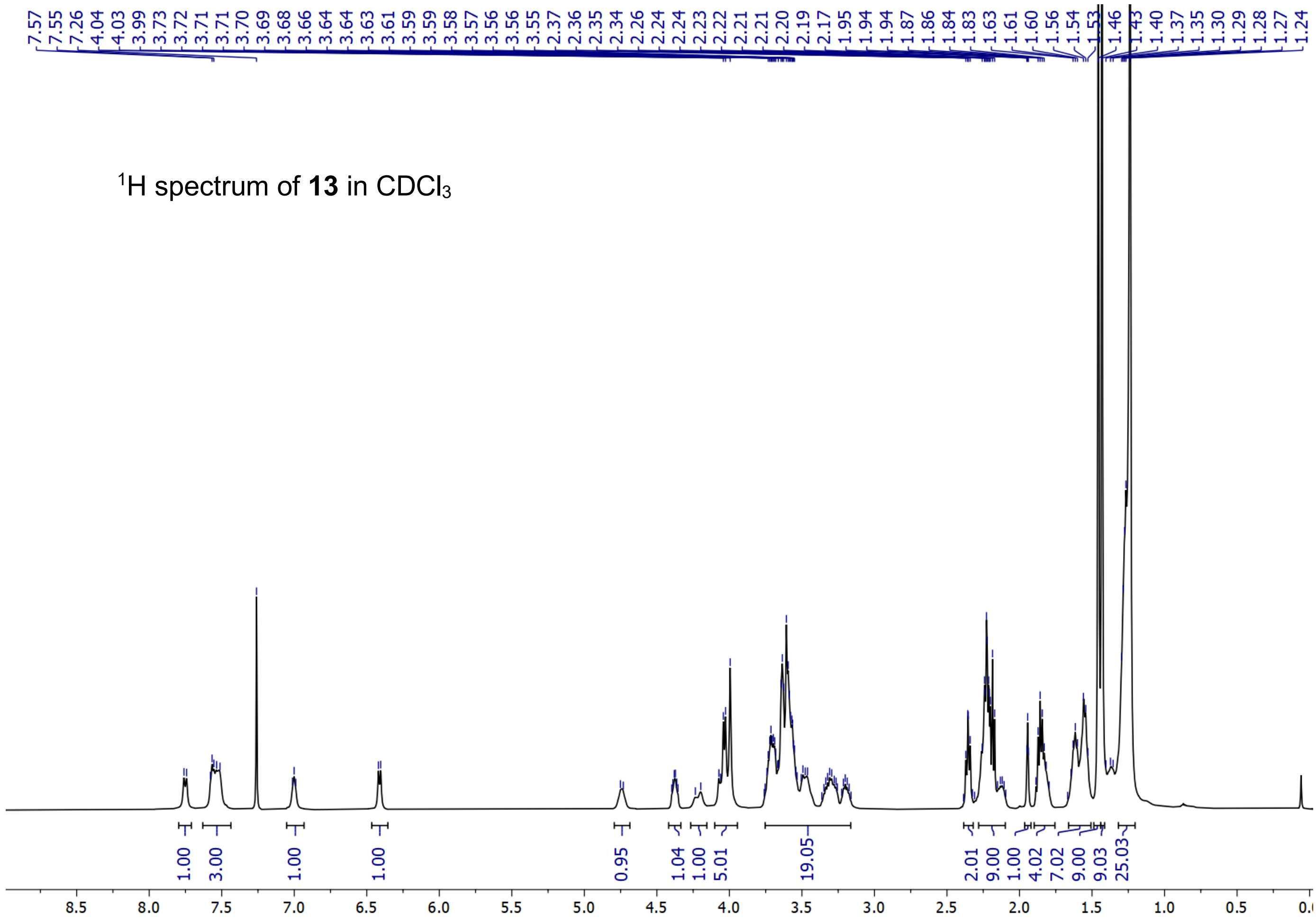

^{1}H spectrum of **13** in $CDCl_3$

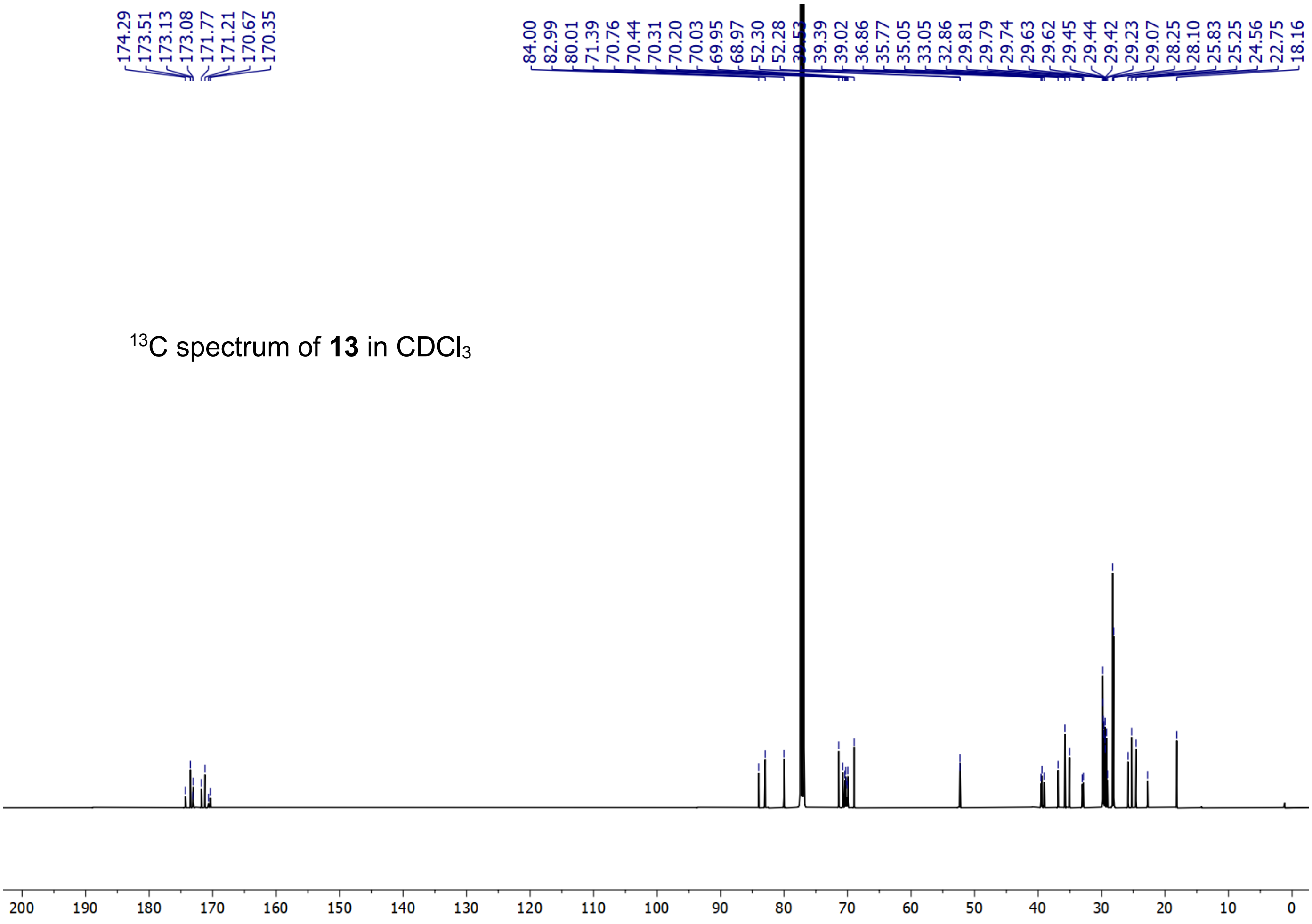

^{13}C spectrum of **13** in $CDCl_3$

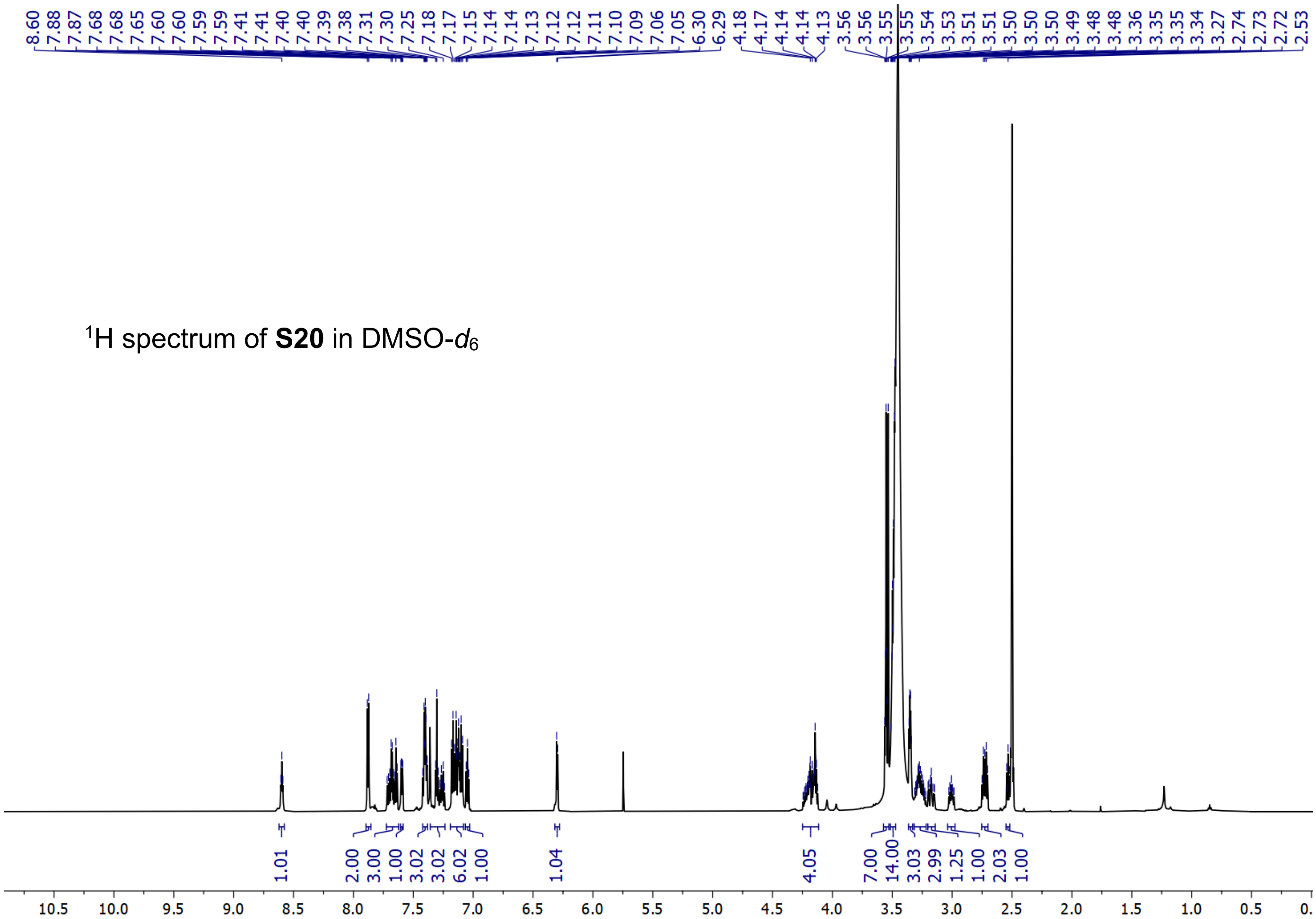

^{1}H spectrum of **S20** in DMSO-d_6

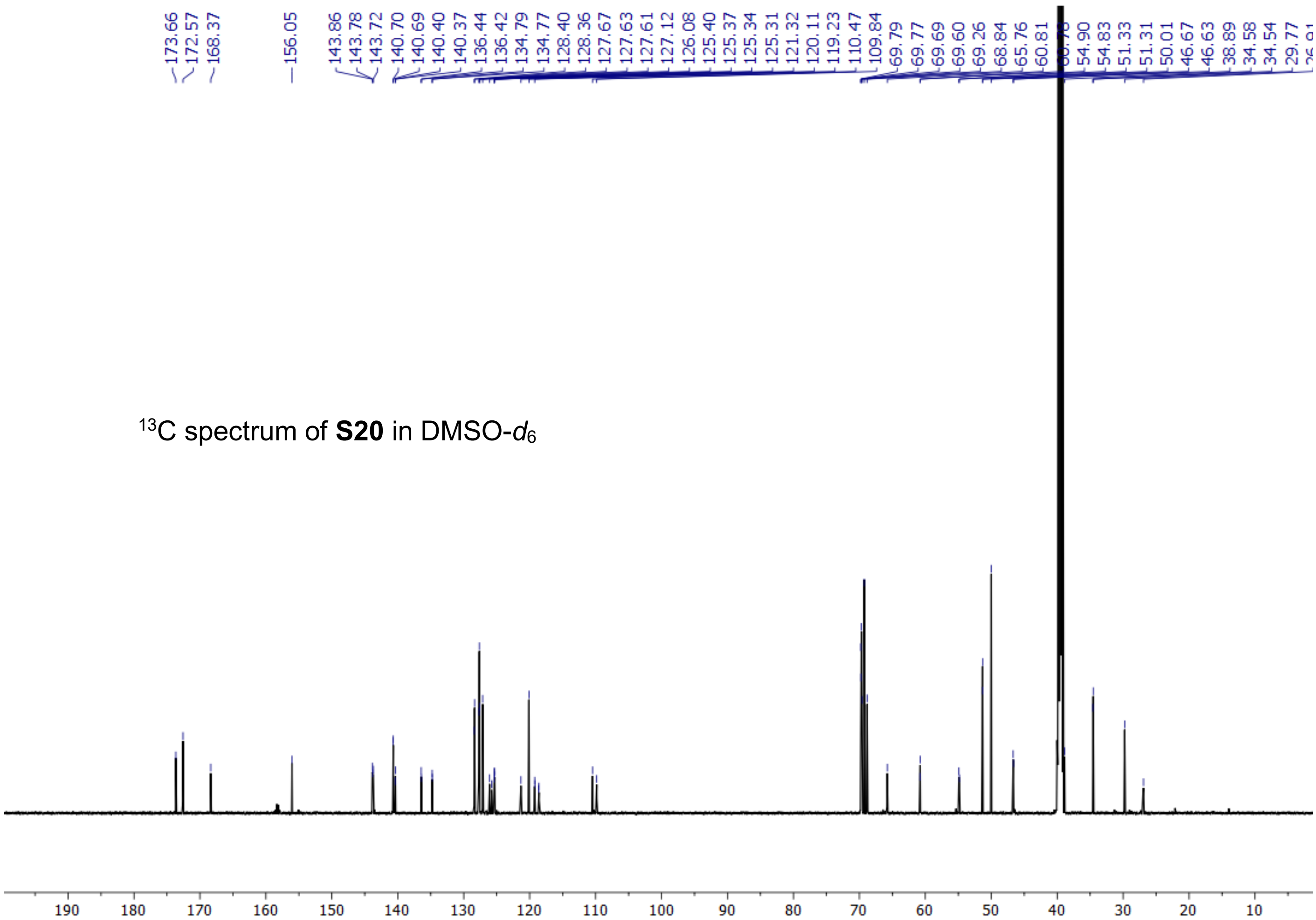

^{13}C spectrum of **S20** in DMSO-d_6
